# Supplementary material for: Cysteine-specific protein multi-functionalization and disulfide bridging using 3-bromo-5-methylene pyrrolones
Source: Nat Commun. 2020 Feb 21;11:1015. doi: 10.1038/s41467-020-14757-4 (PMC7035330; doi:10.1038/s41467-020-14757-4)
Supplement: Supplementary file 1 — Supplementary Information [file 41467_2020_14757_MOESM1_ESM.pdf]

Supplementary Information

Cysteine-Specific Protein Multi-functionalization and Disulfide Bridging Using 3-Bromo-5-Methylene Pyrrolones

By Zhang et al.

Supplementary Methods

General methods and procedures. All chemicals were obtained from commercial supplier and used without further purification. All the dry solvents used for reactions were distilled under argon after drying over an appropriate drying agent. Peptide **19** and endogenous hormone somatostain (SST) were purchased from Shanghai Top-Peptide Biotechnology Co. Ltd. and MedChemExpress. ECL plus Chemiluminescence kit was purchased from Beyotime. Goat Fab anti-human IgG was purchased from SouthernBiotech. Expression and purification of histone H3-V35C was carried out as previously described.[1,2] Cell culture media and fetal bovine serum (FBS) were from Gibco.

The preparative - reverse phase HPLC was conducted on FLEXA HP Series fitted with Venusil prepG C18 column 120 Å 10 μm 21.2 mm x 250 mm (Cat. No.: VX902520-A). The NMR spectra were recorded on Bruker - 400 MHz spectrometers. High resolution mass spectra (HRMS) were recorded on Waters Xevo G2-XS Q-TOF mass spectrometry. Mass spectra were acquired in positive ion mode using a capillary voltage of 3 kV, a sampling cone voltage of 40 V and a source offset voltage of 80 V. The cone gas flow was set up to 50 L/h and desolvation gas flow was 800 L/h. Desolvation temperature and source temperature were set to 400 and 100 °C, respectively. The mass of intact protein was obtained by deconvolution of the raw data using MaxEnt1 tool. Peptide mapping was carried out using MS^E model and the data was analyzed using BiopharmaLynx software.

Reversed-phase (RP) UPLC were run in Waters Acquity® UPLC H-class system with PDA detector. Column I: ACQUITY UPLC BEH C18, 130 Å, 1.7 μm, 2.1 mm X 50 mm. Flow 0.4 mL/min at 35 ºC. Column II: ACQUITY UPLC HSS C18, 300 Å, 1.7 μm, 2.1 mm X 100 mm. Flow 0.4 mL/min at 35 ºC. Column III: ACQUITY UPLC protein BEH C4, 130 Å, 1.7 μm, 2.1 mm X 50 mm. Flow 0.4 mL/min at 60 ºC. Buffer A: water. Buffer B: MeCN. Buffer C: 1% (v/v) formic acid in water.

Synthesis of 1.

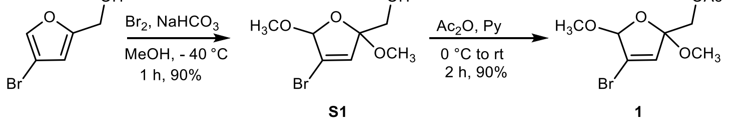

Br_2 (2.6 mL, 50.7 mmol, 1.3 eq.) was added dropwise to a solution of the 3-bromofurfuryl alcohol (6.9 g, 39.0 mmol, 1.0 eq.) and sodium bicarbonate (4.9 g, 58.5 mmol, 1.5 eq.) in dry MeOH (250 mL) at -40 ℃. After 1 h, the reaction mixture was allowed to warm to room temperature and quenched with

saturated $NaHCO_3$. Methanol was removed under reduced pressure. The residue was extracted with DCM. The result organic phase was washed with saturated NaCl. After drying over $MgSO_4$, the organic phase was concentrated to give a crude product **S1** (8.4 g, 90%) which was used without further purification.

The above obtained product was dissolved in dry pyridine (240.0 mL), acetic anhydride (13.0 mL, 14.1 mmol, 4.0 eq.) was added dropwise at 0 ℃. After stirring for 2 h at room temperature, the reaction was quenched with H_2O and concentrated under reduced pressure. The result mixture was diluted with EtOAc and washed with saturated $NaHCO_3$. After drying over $MgSO_4$, the organic phase was concentrated, and the residue was chromatographed on silica gel (EtOAc: PE = 15%) to obtain the compound **1** (8.8 g, 90%) as a colorless oil. ^{1}H NMR (400 MHz, CD_3OD) δ 6.25 (s, 1H), 6.23 (s, 1H), 5.66 (s, 1H), 5.38 (s, 1H), 4.26 - 4.02 (m, 4H), 3.53 (s, 3H), 3.44 (s, 3H), 3.24 (s, 3H), 3.19 (s, 3H), 2.03 (s, 6H). ^{13}C NMR (101 MHz, CD_3OD) δ 172.2, 172.1, 132.7, 132.1, 124.4, 124.3, 113.6, 112.3, 109.8, 108.8, 67.3, 66.9, 57.0, 55.7, 51.2, 50.6, 21.0, 20.8. HRMS (ESI): $C_9H_{13}NaBrO_5$, $[M+Na]^+$ cal. 302.9844, found 302.9897.

Synthesis of 4.

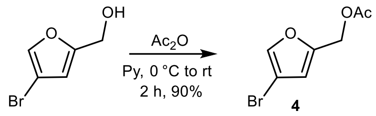

Ac_2O (8.4 mL, 89.3 mmol, 4.0 eq.) was added dropwise to a solution of the 3-bromofurfuryl alcohol (4.0 g, 22.3 mmol, 1.0 eq.) in dry pyridine (150 mL) at 0 □. After stirring for 2 h at room temperature, the reaction was quenched with H_2O and concentrated under reduced pressure. The result mixture was diluted with EtOAc and washed with saturated $NaHCO_3$. After drying over $MgSO_4$, the organic phase was concentrated, and the residue was chromatographed on silica gel (EtOAc: PE = 1: 9) to obtain the compound **4** (4.4 g, 90%) as a yellow oil. ^{1}H NMR (400 MHz, $CDCl_3$) δ 7.41 (m, 1H), 6.46 (s, 1H), 5.01 (s, 2H), 2.08 (s, 3H). ^{13}C NMR (101 MHz, $CDCl_3$) δ 170.6, 150.7, 141.5, 114.0, 100.4, 57.8, 20.9.

Synthesis of 3Br-5MPs 3a-3g by Method 1 was carried out as previously described.[3]

General procedure for one-pot synthesis of 3Br-5MPs from 4 by method 2:

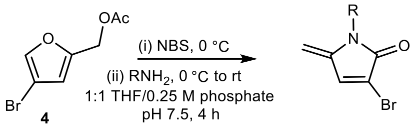

To a solution of **4** (100.0 mg, 0.46 mmol, 1.0 eq.) in 10.0 mL of solvent (5.0 mL THF + 5.0 mL of 0.25 M sodium phosphate buffer, pH 7.5), NBS (98.0 mg, 0.55 mmol, 1.2 eq.) was added. After the solution was stirred for 1 h at 0 □, amine substrate (0.55 mmol, 1.2 eq.) was added and the reaction was stirred for 4 h at room temperature. The reaction mixture was concentrated.

Synthesis of 3a. The crude residue was purified by flash column chromatography (MeOH: DCM = 5% - 10%) to obtain **3a** (64.0 mg, 60%) as a white solid. ^{1}H NMR (400 MHz, d_6-DMSO) δ 7.66 (s, 1H), 7.53 (s, 1H), 7.19 (s, 1H), 5.06 (s, 2H), 4.20 (s, 2H). ^{13}C NMR (101 MHz, d_6-DMSO) δ 168.5, 164.6, 143.8, 136.6, 117.2, 99.0, 42.1. HRMS (ESI): $C_7H_7N_2NaBrO_2$, $[M+Na]^+$ cal. 252.9589, found 252.9608.

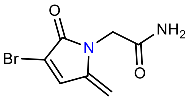

3a

Synthesis of 3b. The crude residue was purified by flash column chromatography (MeOH: DCM = 1: 8) to obtain **3b** (77.0 mg, 50%) as a white solid. ^{1}H NMR (400 MHz, D_2O) δ 7.50 (s, 1H), 5.53 (d, J = 8.0 Hz, 1H), 5.39 (d, J = 2.8 Hz, 1H), 5.28 (d, J = 2.8 Hz, 1H), 4.41 - 4.36 (m, 1H), .3.92 - 3.91 (m, 1H), 3.79 - 3.77 (m, 1H), 3.64 - 3.46 (m, 3H). ^{13}C NMR (101 MHz, D_2O) δ 167.2, 144.8, 137.3, 116.3, 102.0, 91.9, 76.1, 70.6, 70.3, 60.7, 60.4. HRMS (ESI): $C_{11}H_{14}NaBrNO_6$, $[M+Na]^+$ cal. 357.9902, found 357.9907.

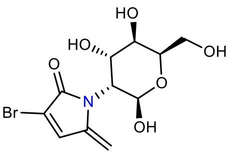

3b

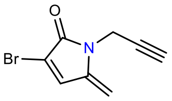

3c

Synthesis of 3c. The crude residue was diluted with water and extracted twice with DCM. The organic layers were pooled, dried with anhydrous $MgSO_4$ and the solvent was removed. The resulting residue was purified by flash column chromatography (EtOAc: PE = 1: 9) to obtain **3c** (51 mg, 52%) as a pale yellow solid. ^{1}H NMR (400 MHz, $CDCl_3$) δ 7.15 (s, 1H), 5.16 (d, J = 2.0 Hz, 1H), 4.99 (d, J = 2.0 Hz, 1H), 4.46 (d, J = 2.4 Hz, 2H), 2.25 (t, J = 2.4 Hz, 1H). ^{13}C NMR (101 MHz, $CDCl_3$) δ 164.3, 142.8, 135.8, 118.5, 98.6, 77.4, 72.6, 29.5. HRMS (ESI): C_8H_7BrNO, $[M+H]^+$ cal. 211.9711, found 211.9712.

Synthesis of 3d. The crude residue was purified by flash column chromatography (MeOH: DCM = 5% - 10%) to obtain **3d** (86.0 mg, 50%) as a yellow solid. ^{1}H NMR (400 MHz, CD_3OD) δ 7.56 (s, 1H), 5.12 (d, *J* = 1.6 Hz, 1H), 5.10 (d, *J* = 1.6 Hz, 1H), 4.52 - 4.49 (m, 1H), 4.34 - 4.31 (m, 1H), 3.24 - 3.21 (m, 1H), 2.94 (dd, *J* = 12.8, 4.8 Hz, 1H), 2.73 - 2.68 (m, 1H), 2.41 (t, *J* = 7.2 Hz, 2H), 1.79 - 1.64 (m, 4H), 1.60 - 1.50 (m, 2H). ^{13}C NMR (101 MHz, CD_3OD) δ 175.2, 166.3, 164.5, 144.3, 136.7, 117.9, 99.3, 63.5, 61.8, 57.1, 41.2, 34.3, 29.7, 29.5, 26.4. HRMS (ESI): $C_{15}H_{20}BrN_4O_3S$, $[M+H]^+$ cal. 415.0439, foun d 415.0451.

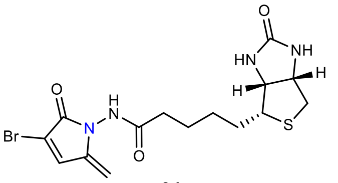

3d

Synthesis of 3e. The crude residue was purified directly by preparative RP HPLC to afford **3e** (104 mg, 45%) as an orange solid. RP HPLC conditions: C18, 10 µm, 21.2 mm X 250 mm. Flow 15 mL/min at rt. Buffer A: water. Buffer B: MeCN. 0 - 10 - 20 min, B: 5% - 40% - 85%. Retention time of **3e** is 21.5 min. ^{1}H NMR (400 MHz, *d*$_6$-DMSO) δ 7.93 (d, *J* = 1.6 Hz, 1H), 7.92 (s, 1H), 7.73 (dd, *J* = 8.0, 1.6 Hz, 1H), 7.40 (d, *J* = 8.0 Hz, 1H), 6.70 - 6.88 (m, 3H), 6.66 (s, 1H), 6.59 (d, *J* = 2.4 Hz, 1H), 6.57 (d, *J* = 2.4 Hz, 1H), 5.27 (d, *J* = 2.0 Hz, 1H), 5.12 (d, *J* = 2.0 Hz, 1H). ^{13}C NMR (101 MHz, *d*$_6$-DMSO) δ 168.0, 164.1, 160.3, 152.2, 144.0, 137.4, 135.4, 134.7, 129.4, 128.0, 125.5, 123.9, 116.9, 113.2, 109.5, 102.5, 101.2. HRMS (ESI): $C_{25}H_{15}BrNO_6$, $[M+H]^+$ cal. 504.0083, found 504.0086.

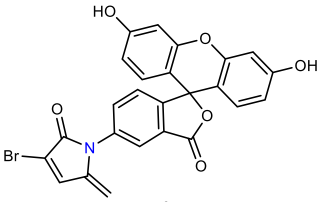

3e

Synthesis of 3f. The crude residue was purified directly by preparative RP HPLC to afford **3f** (150 mg, 47%) as a red solid. RP HPLC conditions: C18, 10 µm, 21.2 mm X 250 mm. Flow 15 mL/min at rt. Buffer A: water. Buffer B: MeCN. 0 - 10 - 20 min, B: 5% - 40% - 85%. Retention time of **3f** is 23.0 min. ^{1}H NMR (400 MHz, $CDCl_3$) δ 13.88 (s, 1H), 13.15 (s, 1H), 7.97 (d, *J* = 7.6 Hz, 1H), 7.76 (t, *J* = 8.0 Hz, 1H), 7.37 (d, *J* = 8.4 Hz, 1H), 7.08 (s, 1H), 5.62 (m, 1H), 5.33 (s, 1H), 5.17 (s, 1H), 4.95 (d, *J* = 2.4 Hz, 1H), 4.90 (d, *J* = 2.4 Hz, 1H), 4.76 (brs, 1H), 4.74 (s, 1H), 4.13 - 4.09 (m, 2H), 4.06 (s, 3H), 3.94 (brs, 1H), 3.22 (d, *J* =18.8 Hz, 1H), 3.05 (brs, 1H), 2.95 (d, *J* = 19.2 Hz, 1H), 2.85 - 2.79 (m, 1H), 2.40 (d, *J* = 14.8 Hz, 1H), 2.20 - 2.15 (m, 1H), 1.71 - 1.67 (m, 2H), 1.36 (d, *J* = 6.4 Hz, 3H). ^{13}C NMR (101 MHz, $CDCl_3$) δ 213.5, 186.9, 186.6, 167.0, 161.0, 156.1, 155.5, 143.5, 136.3, 135.8, 135.3, 133.5, 120.7, 119.8, 118.5, 118.2, 111.5, 111.4, 100.6, 99.8, 76.8, 70.0, 69.6, 69.0,

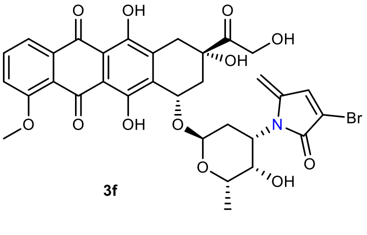

3f

65.4, 56.7, 52.6, 35.4, 33.9, 28.0, 17.3. HRMS (ESI): $C_{32}H_{30}NaBrNO_{12}$, $[M+Na]^+$ cal. 722.0849, found 722.0894.

Synthesis of 3g. The crude residue was diluted with water and extracted twice with DCM. The organic layers were pooled, dried with anhydrous $MgSO_4$ and the solvent was removed. The resulting residue

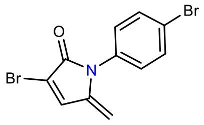

3g

was purified by flash column chromatography (EtOAc: PE = 1: 9) to obtain **3g** (98 mg, 65%) as a solid. ^{1}H NMR (400 MHz, *d*$_6$-DMSO) δ 7.87 (s, 1H), 7.72 (d, J = 8.8 Hz, 2H), 7.31 (d, J = 8.8 Hz, 2H), 5.19 (d, J = 1.6 Hz, 1H), 4.91 (d, J = 1.6 Hz, 1H). ^{13}C NMR (101 MHz, *d*$_6$-DMSO) δ 163.8, 144.0, 136.9, 133.1, 132.4, 129.9, 121.1, 116.9, 100.4. HRMS (ESI): $C_{11}H_8Br_2NO$, $[M+H]^+$ cal. 327.8973, found 327.8976.

Synthesis of 6.

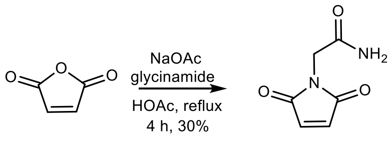

6

A solution of glycinamide hydrochloride (1.7 g, 15.3 mmol, 1.5 eq.), maleic anhydride (1.0 g, 10.2 mmol, 1.0 eq.) and sodium acetate (1.3 g, 15.3 mmol, 1.5 eq.) in acetic acid (20 mL) was stirred for 4 h at reflux temperature (110 ℃). After the reaction mixture was cooled to r.t, chilled sodium bicarbonate solution was added and the product was extracted with DCM twice. The result organic phase was washed with saturated NaCl. After drying over $MgSO_4$, The organic solvent was removed on vacuum and the residue was purified by silica gel column chromatography (MeOH: DCM = 1: 10) to give 6 (0.48 g, 30%) as a yellow solid. ^{1}H NMR (400 MHz, *d*$_6$-DMSO) δ 7.61 (s, 1H), 7.22 (s, 1H), 7.09 (s, 2H), 3.98 (s, 2H). ^{13}C NMR (101 MHz, *d*$_6$-DMSO) δ 170.8, 168.0, 134.9, 39.6. HRMS (ESI): $C_6H_7N_2O_3$, $[M+H]^+$ cal. 155.0457, found 155.0470.

Large scale synthesis of 7.

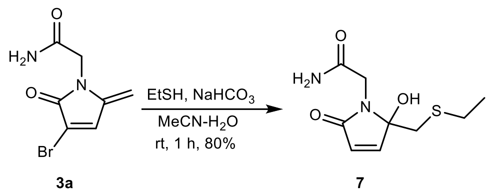

To a solution of **3a** (1.5 g, 6.5 mmol, 1.0 eq.) in 150.0 mL of solvent (70.0 mL H_2O + 80.0 mL CH_3CN), ethanethiol (730.0 μL, 9.8 mmol, 1.5 eq.) and $NaHCO_3$ (547.0 mg, 6.5 mmol, 1.0 eq.) were added. After stirring for 1 h at room temperature, the reaction mixture was concentrated under reduced pressure. The residue was chromatographed on silica gel (MeOH: DCM = 5%) to obtain the compound **7** (1.2 g, 80%) as a pale yellow oil. ^{1}H NMR (400 MHz, d_6-DMSO) δ 7.24 (brs, 1H), 7.14 (brs, 1H), 7.12 (d, J = 6.0 Hz, 1H), 6.40 (s, 1H), 6.12 (d, J = 6.0 Hz, 1H), 3.77 (d, J = 17.2 Hz, 1H), 3.69 (d, J = 17.2 Hz, 1H), 2.91 (d, J = 14.0 Hz, 1H), 2.82 (d, J = 14.0 Hz, 1H), 2.50 - 2.45 (m, 2H), 1.10 (t, J = 7.2 Hz, 3H). ^{13}C NMR (101 MHz, d_6-DMSO) δ 170.9, 169.3, 150.4, 126.0, 91.3, 41.2, 36.9, 26.7, 14.8. HRMS (ESI): $C_9H_{14}N_2O_3SNa$, $[M+Na]^+$ cal. 253.0623, found 253.0611. The structure of **7** was further characterized by 2D NMR spectrum.

Large scale synthesis of 8.

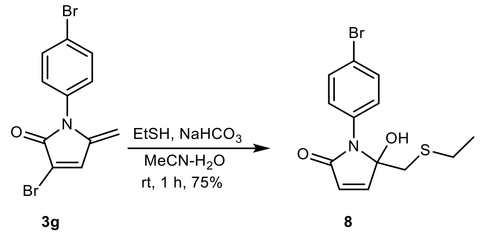

To a solution of **3g** (430 mg, 1.31 mmol, 1.0 eq.) in 45.0 mL of solvent (15.0 mL H_2O + 30.0 mL CH_3CN), ethanethiol (145.0 μL, 1.97 mmol, 1.5 eq.) and $NaHCO_3$ (110 mg, 1.31 mmol, 1.0 eq.) were added. After stirring for 1 h at room temperature, the reaction mixture was concentrated under reduced pressure. The residue was chromatographed on silica gel (EtOAc: PE = 20 - 50%) to obtain the compound **8** (310 mg, 75%) as a white solid. ^{1}H NMR (400 MHz, CD_3OD) δ 7.57 (d, J = 8.0 Hz, 2H), 7.48 (d, J = 8.0 Hz, 2H), 7.16 (d, J = 6.0 Hz, 1H), 6.26 (d, J = 6.0 Hz, 1H), 2.98 (d, J = 14.0 Hz, 1H), 2.75 (d, J = 14.0 Hz, 1H), 2.47 - 2.41 (m, 2H), 1.12 (t, J = 7.2 Hz, 3H). ^{13}C NMR (101 MHz, CD_3OD) δ 169.8, 149.8, 134.6, 131.6, 128.1, 126.3, 119.9, 94.2, 36.6, 27.0, 13.7. HRMS (ESI): $C_{13}H_{15}BrNO_2S$, $[M+Na]^+$ cal. 328.0007, found 327.9989.

Large scale synthesis of 9.

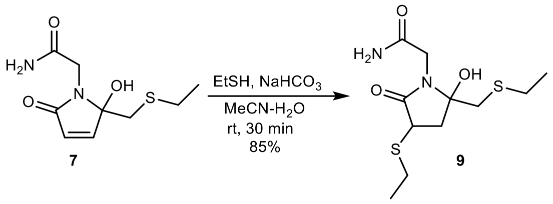

To a solution of **7** (600 mg, 2.61 mmol, 1.0 eq.) in 50 mL of solvent (20 mL H_2O + 30 mL CH_3CN), ethanethiol (290 μL, 3.91 mmol, 1.5 eq.) and $NaHCO_3$ (215 mg, 2.61 mmol, 1.0 eq.) were added. After stirring for 30 min at room temperature, the reaction mixture was concentrated under reduced pressure. The residue was chromatographed on silica gel (MeOH: DCM = 5%) to obtain the compound **9** (650.0 mg, 85%) as a pale yellow oil and it was a pair of diastereoisomers. ^{1}H NMR (400 MHz, d_6-DMSO) δ 7.40 (s, 1H), 7.27 (s, 1H), 7.16 (s, 1H), 7.14 (s, 1H), 6.26 (s, 2H), 3.77 - 3.64 (m, 4H), 2.89 - 2.38 (m, 16H), 2.12 (dd, *J* = 7.7, 13.6 Hz, 1H), 1.82 (dd, *J* = 8.0, 13.6 Hz, 1H), 1.16 - 1.09 (m, 12H). ^{13}C NMR (101 MHz, d_6-DMSO) δ 173.5, 172.4, 171.0, 170.8, 89.6, 89.5, 42.6, 42.4, 41.7, 40.9, 40.7, 40.0, 39.7, 26.9, 26.8, 24.9, 24.8, 15.4, 15.3, 15.0, 14.9. HRMS (ESI): $C_{11}H_{20}N_2O_3S_2Na$, $[M+Na]^+$ cal. 315.0813, found 315.0828.

Large scale synthesis of 17.

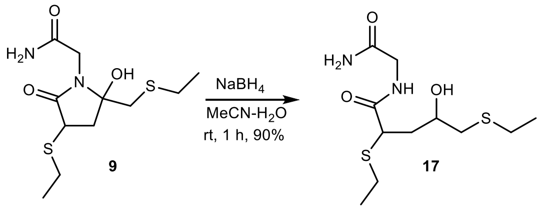

To a solution of **9** (283.0 mg, 0.96 mmol, 1.0 eq.) in 30 mL of solvent (9 mL H_2O + 21 mL CH_3CN), $NaBH_4$ (41 mg, 1.05 mmol, 1.1 eq.) was added. After stirring for 1 h at room temperature, the reaction mixture was concentrated under reduced pressure. The residue was chromatographed on silica gel (MeOH: DCM = 5% - 10%) to obtain the compound **17** (254 mg, 90%) as a white solid. ^{1}H NMR (400 MHz, d_6-DMSO) δ 8.19 (t, *J* = 5.6 Hz, 1H), 7.23 (s, 1H), 7.04 (s, 1H), 4.91 (d, *J* = 5.2 Hz, 1H), 3.79 - 3.77 (m, 1H), 3.70 (dd, *J* = 16.8, 6.0 Hz, 1H), 3.59 (dd, *J* = 16.8, 5.6 Hz, 1H), 3.48 (dd, *J* = 6.0, 2.8 Hz, 1H), 2.61 - 2.48 (m, 6H), 1.87 - 1.69 (m, 2H), 1.18 (t, *J* = 7.2 Hz, 3H), 1.15 (t, *J* = 7.2 Hz, 3H). ^{13}C NMR (101 MHz, d_6-DMSO) δ 172.0, 170.9, 67.4, 44.3, 41.9, 38.3, 37.6, 25.8, 24.1, 14.9, 14.6. HRMS (ESI): $C_{11}H_{22}N_2O_3S_2Na$, $[M+Na]^+$ cal. 317.0970, found 317.0974. The structure of **17** was

characterized by 2D NMR spectrum.

Large scale synthesis of 18.

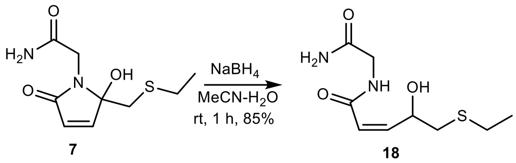

To a solution of **7** (160.0 mg, 0.69 mmol, 1.0 eq.) in 9.0 mL of solvent (3.0 mL H_2O + 6.0 mL CH_3CN), $NaBH_4$ (29.5 mg, 0.76 mmol, 1.1 eq.) was added. After stirring for 1 h at room temperature, the reaction mixture was concentrated under reduced pressure. The residue was chromatographed on silica gel (MeOH: DCM = 1: 9) to obtain the compound **18** (136.0 mg, 85%) as a colorless oil. ^{1}H NMR (400 MHz, d_6-DMSO) δ 8.30 (t, J = 6.0 Hz, 1H), 7.36 (s, 1H), 7.04 (s, 1H), 5.96 (dd, J = 11.6, 7.2 Hz, 1H), 5.88 (d, J = 12.0 Hz, 1H), 5.32 (d, J = 5.2 Hz, 1H), 5.21 - 5.15 (m, 1H), 3.69 (d, J = 6.0 Hz, 2H), 2.59 - 2.50 (m, 4H), 1.17 (t, J = 7.2 Hz, 3H). ^{13}C NMR (101 MHz, d_6-DMSO) δ 170.8, 165.5, 147.1, 121.9, 66.4, 41.7, 37.8, 25.6, 14.8. HRMS (ESI): $C_9H_{16}N_2O_3SNa$, $[M+Na]^+$ cal. 255.0779, found 255.0798. The structure of **18** was characterized by 2D NMR spectrum.

Reaction of peptide 19 with 3a, 5, 6 and 7.

For 2 equiv reactions: A mixture of **3a**, **5**, **6** or **7** (0.4 mM) and peptide **19** (0.2 mM) in HEPES buffer (20 mM, pH 7.5, 100 mM NaCl) was incubated at 4 ºC and 37 ºC for 5 min. Aliquots were analyzed by UPLC-MS/MS. For reaction of **7** at 37 ºC, the reaction was extended to 1 h.

For 10 equiv reactions: A mixture of **3a**, **5**, **6** and **7** (2 mM) and peptide **19** (0.2 mM) in HEPES buffer (20 mM, pH 7.5, 100 mM NaCl) was incubated at 37 ºC for 1 h. Aliquots were analyzed by UPLC-MS/MS. UPLC condition (column I): 0-6 min, B: 5-20%, C: 10% keep constant. The overall yield and selectivity were calculated by integral of LC chromatogram and MS/MS analysis.

Reaction of protein H3-V35C with 3Br-5MPs.

Protein H3-V35C sequence:

ARTKQ TARKS TGGKA PRKQL ATKAA RKSAP ATGGC^{35} KKPHR YRPGT VALREI RRYQK STELL IRKLP FQRLV REIAQ DFKTD LRFQS SAVMA LQEAS EAYLV ALFED TNLAA IHAKR VTIMP KDIQL ARRIR GERA

Calculated Mass: 15242

A mixture of 3Br-5MPs (**3a, 3b, 3c, 3d, 3e** or **3f**, 20 μM) and H3-V35C (10 μM) in HEPES buffer (20 mM, pH 7.5, 100 mM NaCl) was incubated at 37 °C for 1 h. Aliquots were taken and analyzed by UPLC-MS. UPLC conditions (column III): 0-7.5 min, B: 2-80%, C: 10% keep constant. The mass of intact protein was obtained by deconvolution of the raw data using MaxEnt1 tool.

Preparation of modified protein 21.

A mixture of **3e** (200 μM) and H3-V35C (100 μM) in HEPES buffer (20 mM, pH 7.5, 100 mM NaCl) was incubated at 37 °C for 1 h. Excess **3e** and salts were removed by PD Minitrap G-25 column (GE Healthcare). The reaction yield was calculated based on MS peak intensity. The recovery of modified protein **21** was determined by $A_{280\ nm}$ to be 85% (ε_{280} of protein H3-V35C: 4595 $M^{-1}cm^{-1}$; ε_{280} of fluorescein: 14400 $M^{-1}cm^{-1}$).

Preparation of modified protein 23.

A mixture of **3e** (200 μM) and H3-V35C (100 μM) in HEPES buffer (20 mM, pH 7.5, 100 mM NaCl) was incubated at 37 °C for 1 h. Without purification, Biotin-SH[4] (**22**, 700 μM) was added to the solution mixture directly followed by incubation at 37 °C for 2 h. Then excess small molecules and salts were removed by PD Minitrap G-25 column (GE Healthcare). The reaction yield was calculated based on MS peak intensity. The recovery of multi-functional modified protein was determined by $A_{280\ nm}$ to be 80%.

Stability of protein H3-V35C upon treatment with $NaBH_4$.

To a solution of protein H3-V35C (10 μM) in HEPES buffer (20 mM, pH 7.5, 100 mM NaCl) was added 100 mM $NaBH_4$ solution (freshly prepared, final concentration 3 mM, 10 mM or 20 mM) followed by incubation at 37 °C for 1 h. 2 μL of the mixture was taken and analyzed by UPLC-MS. UPLC conditions (column III): 0-7.5 min, B: 2-80%, C: 10% keep constant. The mass of intact protein was obtained by deconvolution of the raw data using MaxEnt1 tool.

Preparation of reduced protein conjugate 25.

A mixture of **3e** (200 μM) and H3-V35C (100 μM) in HEPES buffer (20 mM, pH 7.5, 100 mM NaCl)

was incubated at 37 °C for 1 h. Without purification, Biotin-SH[4] (**22**, 700 μM) was added to the solution mixture directly followed by incubation at 37 °C for 2 h. 100 mM $NaBH_4$ solution (freshly prepared, final concentration 3 mM) was added directly into the reaction mixture and incubated at 37 °C for 40 min. Then excess small molecules and salts were removed by PD Minitrap G-25 column (GE Healthcare). The reaction yield was calculated based on MS peak intensity. The recovery of reduced protein **25** was determined by $A_{280\ nm}$ to be 80%.

Trypsin digestion and UPLC-MS/MS analysis of conjugate 25: Above pure modified protein **25** (0.2 μg/μL) and trypsin (0.5 μg/μL) were mixed in a 50 μL buffer containing 50 mM NH_4HCO_3 and 1 mM $CaCl_2$. The reaction mixture was incubated at 37 °C for 3 h, quenched with TFA to final concentration of 1%. Sample (10 μL) was analyzed by UPLC-MS/MS analysis.

Preparation of reduced protein 26.

A mixture of **3e** (200 μM) and H3-V35C (100 μM) in HEPES buffer (20 mM, pH 7.5, 100 mM NaCl) was incubated at 37 °C for 1 h. 100 mM $NaBH_4$ solution (freshly prepared, final concentration 3 mM) was added directly into the reaction mixture and incubated at 37 °C for 40 min. Excess small molecules and salts were removed by PD Minitrap G-25 column (GE Healthcare).

Stability of modified protein 23 in neutral aqueous solutions.

Pure modified protein **23** (40 μM) was incubated in HEPES buffer (20 mM, pH 7.5, 100 mM NaCl, 0.1 μg/μL BSA) or the same buffer with 1 mM GSH at r.t. Aliquots were taken at 0, 24, 48, 72 h. The samples were mixed with SDS loading buffer (50 mM Tris, pH 6.8, glycol 10% (v/v), SDS 2% (w/v), bromophenol 0.1% (w/v)) and analyzed by 15% SDS-PAGE. Fluorescein intensity of each band was quantified using GE Typhoon Gel Imaging Scanner with excitation at 488 nm and emission at 520 nm. Western blotting against biotin was performed following a standard procedure: Proteins were transferred to a PVDF membrane using 100 V for 10 min followed by 60 V for 20 min in ice bath. The membrane was blocked by 5 % dry milk in TTBS for 2 h at r.t., followed by washing with TTBS for 10 min×3. Then soaked in a solution of streptavidin-peroxidase in TTBS (1:2000, v/v) at r.t. for another 2 h, and washed with TTBS for 5 min×3. The membrane was detected using an ECL Plus chemiluminescence kit according to the manufacturer's instructions. Chemiluminescence intensity of

each band was quantified using GE Typhoon Gel Imaging Scanner.

As a control, another set of samples were analyzed by 15% SDS-PAGE and stained by commassie blue.

Stability of reduced protein conjugate 25.

Reduced protein conjugate **25** (40 μM) was incubated in different buffers (① HEPES, 20 mM, pH 7.5, 100 mM NaCl, 0.1 μg/μL BSA; ② HEPES, 20 mM, pH 7.5, 100 mM NaCl, 0.1 μg/μL BSA, 1 mM GSH) at r.t. 16 μL of mixture were taken at 0, 24, 48, 72 h. Half volume of the sample was mixed with SDS loading buffer (50 mM Tris, pH 6.8, glycol 10% (v/v), SDS 2% (w/v), bromophenol 0.1% (w/v)) and analyzed by 15% SDS-PAGE. Fluorescein intensity of each band was quantified using GE Typhoon Gel Imaging Scanner with excitation at 488 nm and emission at 520 nm.

Then quantify biotin by western blotting following the procedure described above.

As a control, another set of samples was analyzed by 15% SDS-PAGE and stained by commassie blue.

Stability of reduced protein conjugate 26.

Pure protein conjugated **26** (40 μM) was incubated in different buffers (① HEPES, 20 mM, pH 7.5, 100 mM NaCl, 0.1 μg/μL BSA; ② HEPES, 20 mM, pH 7.5, 100 mM NaCl, 0.1 μg/μL BSA, 1 mM GSH) at r.t. Aliquots were taken at 0, 24, 48, 72 h, mixed with SDS loading buffer (50 mM Tris, pH 6.8, glycol 10% (v/v), SDS 2% (w/v), bromophenol 0.1% (w/v)) and analyzed by 15% SDS-PAGE. Fluorescein intensity of each band was quantified using GE Typhoon Gel Imaging Scanner with excitation at 488 nm and emission at 520 nm.

Disulfide bridging bioconjugation of SST using 3Br-5MP.

A mixture of TCEP (0.075 mM, 1.5 equiv) and hormone somatostain (SST) (0.05 mM) in HEPES buffer (20 mM, pH 7.5, 100 mM NaCl) was incubated at 37 ºC for 2 h. Without purification, **3a** or **3e** (0.065 mM, 1.3 equiv) were added into the reaction mixture followed by incubation at same temperature for 1 h. 100 mM $NaBH_4$ solution (freshly prepared, final concentration 2.5 mM) was added followed by incubation for another 40 min. Aliquots were taken and analyzed by UPLC-MS. UPLC conditions (column I): 0-6 min, B: 5-60%, C: 10% keep constant.

In order to verify the disulfide bridging bioconjugation structure, products **29** and **30** were purified by

HPLC, and subjected to Ellman's test:[5] A solution of **29** or **30** (0.06 mM) in water was treated with Ellman's reagent (1.2 mM in H_2O and DMSO (1:1, v/v), 100 equiv) at 37 °C for 3 h, after which the mixture was analyzed by UPLC-MS. Analysis showed that no reaction between modified peptide and Ellman's reagent, highlighting the structure of **29** and **30** with disulfide bridged conjugation.

Reaction of SST with different equivalent of 3a.

A mixture of TCEP (0.075 mM, 1.5 equiv) and hormone somatostain (SST) (0.05 mM) in HEPES buffer (20 mM, pH 7.5, 100 mM NaCl) was incubated at 37 °C for 2 h. Without purification, different equivalent of **3a** (1.3, 3, 7, 9, 11, 13, 15, 20, 25 equiv) were added into the reaction mixture followed by incubation at same temperature for 1 h. After which the mixture was analyzed by UPLC-MS. UPLC conditions (column I): 0-6 min, B: 5-60%, C: 10% keep constant.

Preparation of disulfide bridging bioconjugation of Fab fragment (33) using 3e.

A mixture of goat Fab anti-human IgG (0.5 mg/mL, approximately molecular weight 36 KDa) and DTT (0.56 mM, 40 equiv) in phosphate buffer (20 mM, pH 8.2) was incubated at 37 °C for 2 h. Excess DTT was removed by PD Spintrap G-25 column (GE Healthcare). The recovery of protein was determined by $A_{280\ nm}$ to be 67%. **3e** (0.02 mM, 1.4 equiv) was added into the protein solution and the mixture was incubated at same temperature for 1 h, followed by adding 100 mM $NaBH_4$ solution (freshly prepared, final concentration 3 mM). After 40 min incubation at r.t., dehydrogenated ascorbic acid (final 0.14 mM, 14 equiv) was added followed by incubation for another 3 h. Excess small molecules and salts were removed by PD Spintrap G-25 column (GE Healthcare). The modification efficiency was analyzed by 15% SDS-PAGE and UV-Vis spectroscopy (Supplementary Figure 40). SDS loading buffer (50 mM Tris, pH 6.8, glycol 10% (v/v), SDS 2% (w/v), bromophenol 0.1% (w/v)). Fluorescein intensity of each band was quantified using GE Typhoon Gel Imaging Scanner with excitation at 488 nm and emission at 520 nm. Finally, the gel was stained by coommassie blue.

Mammalian cell culture

Breast cancer cell lines SK-BR-3 was purchased from Shanghai Zhong Qiao Xin Zhou Biotechnology Co.,Ltd. SK-BR-3 cells were maintained at 37˚C, 5% CO_2 in Dulbecco's modified Eagle's medium complemented with 10% fetal bovine serum and 1% penicillin-streptomycin.

Fluorescence microscopy

SK-BR-3 cells were seeded on TC-treated coverslips in 24-well plates at a density of 1.0 × 10^5/well and cultured in an incubator at 37˚C and 5% CO_2 overnight. Cells were rinsed with PBS (3 × 400 µL) and fixed with 4% paraformaldehyde in PBS (300 µL) at r.t. for 20 min. Then the fixed cells were washed with PBS buffer (3 × 400 µL) and blocked with 10% goat serum for 30 min. After that, the cells were incubated with trastuzumab (500 nM) in PBS for 30 min at r.t. then incubated with goat anti-human antibody-Alexa488 conjugate (1:200) or equivalent pure **33** (1:200) for another 30 min. Cells were washed 3 times with PBS, nuclei were stained with DAPI (300 µL, 10 µg/mL) at r.t. for 7 min. After extensively washing, cells were mounted with antifade mounting medium (Beyotime, P0126). The coverslips were sealed using nail varnish. Cell images were taken using a 60 × oil immersion objective (NA 1.40) of a Nikon A1+ confocal microscope (Nikon, Tokyo, Japan).

(A)

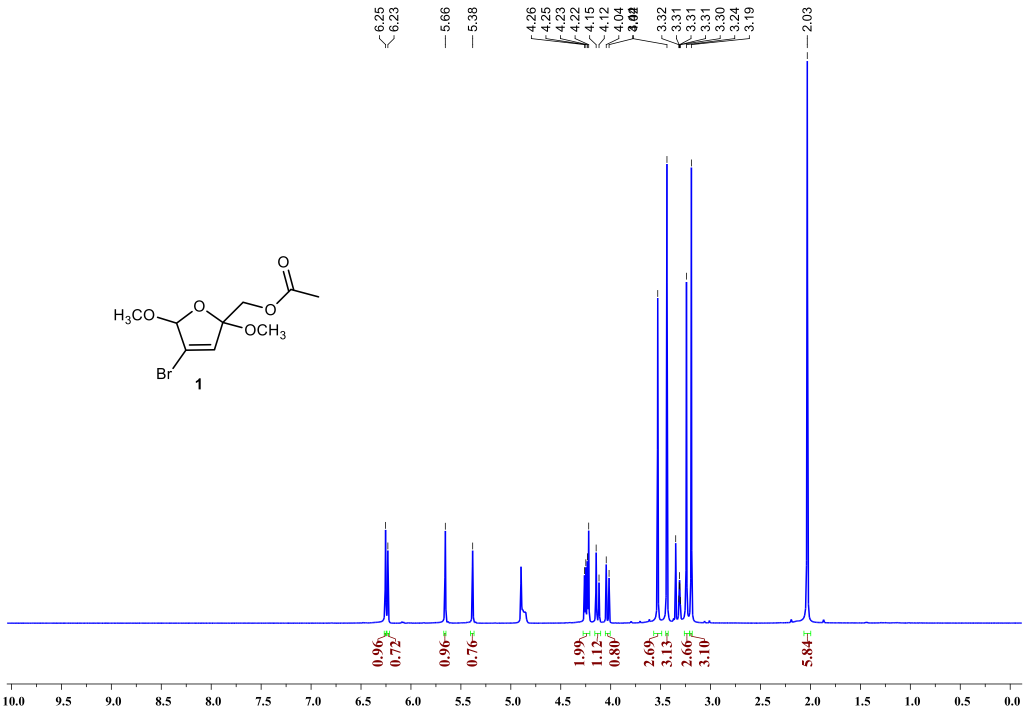

(B)

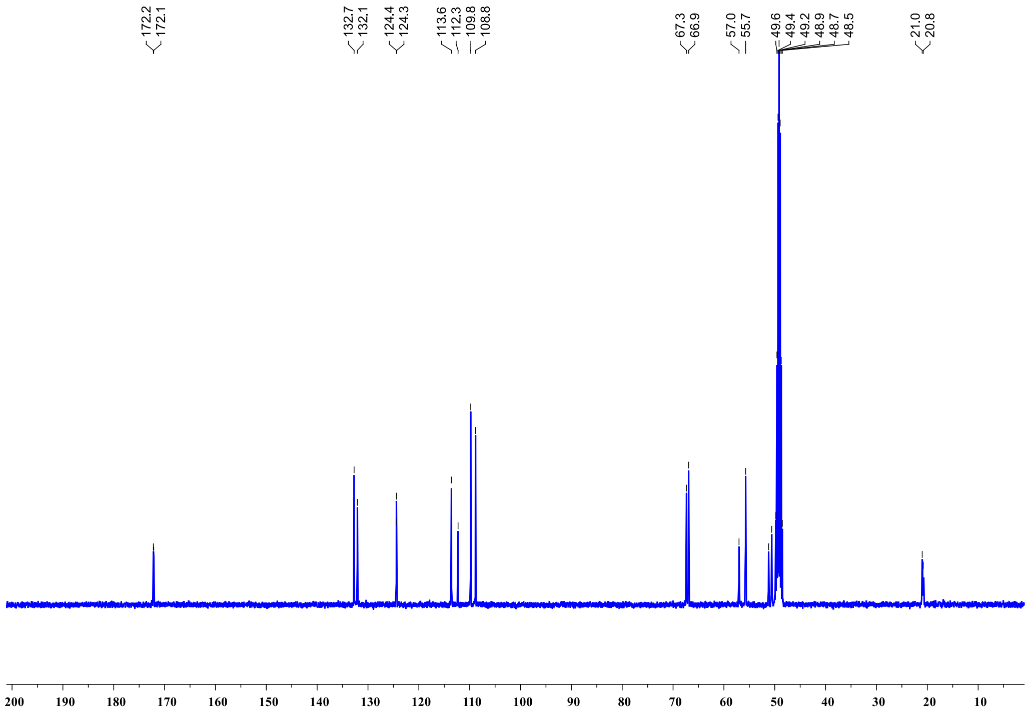

Supplementary Figure 1. NMR spectra of **1**. (A) ^{1}H NMR spectrum of **1**. (B) ^{13}C NMR spectrum of **1**.

(A)

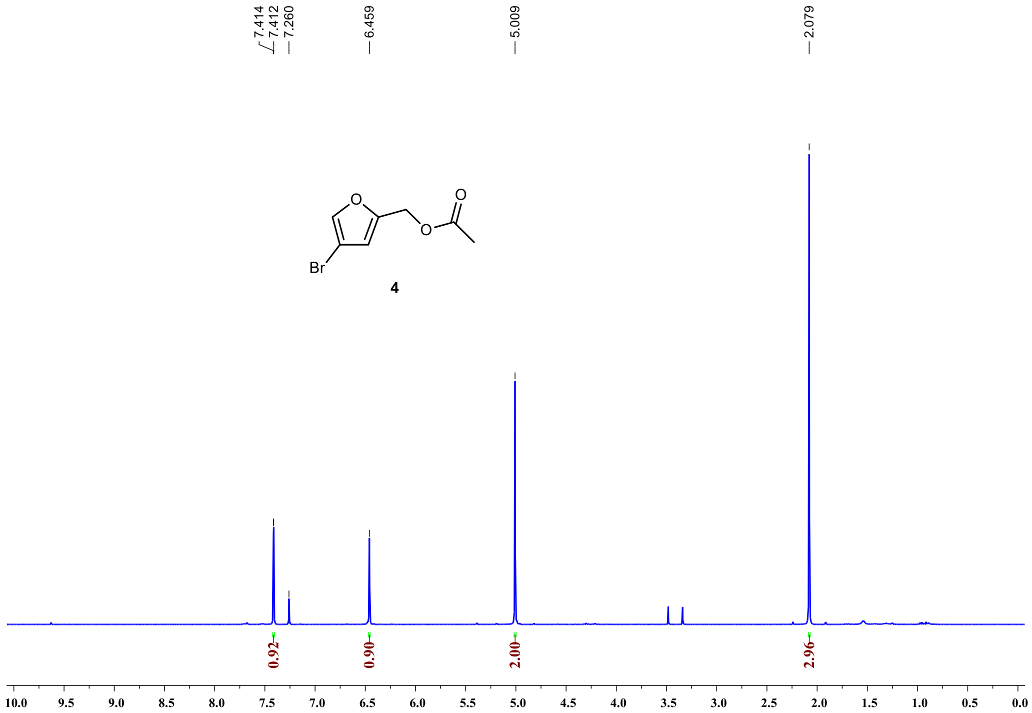

(B)

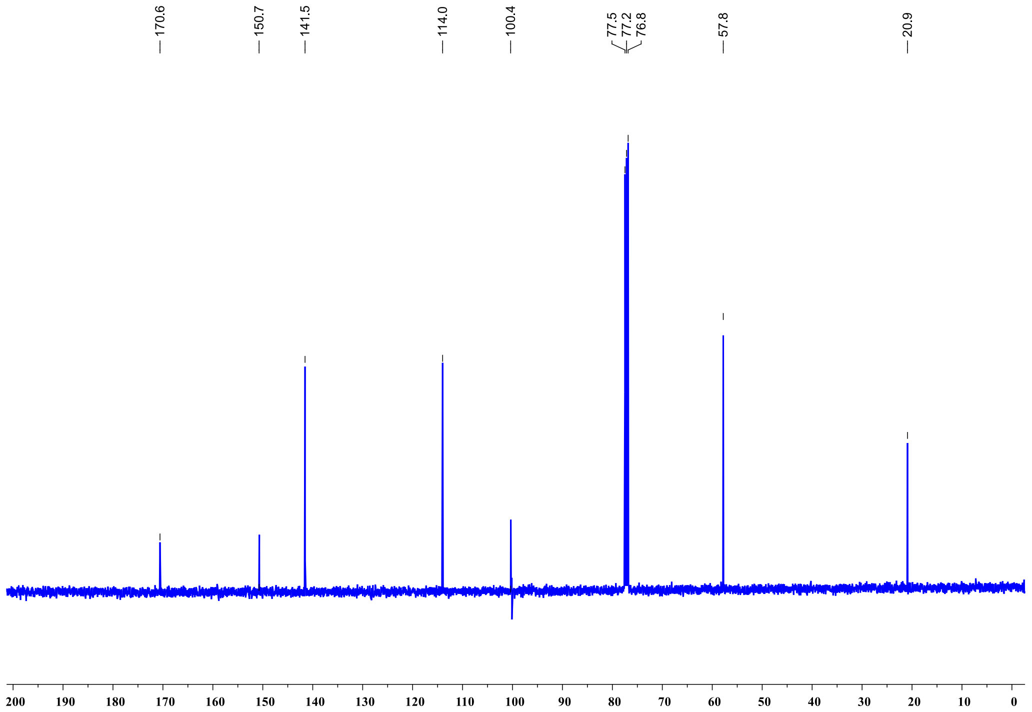

Supplementary Figure 2. NMR spectra of **4**. (A) ^{1}H NMR spectrum of **4**. (B) ^{13}C NMR spectrum of **4**.

(A)

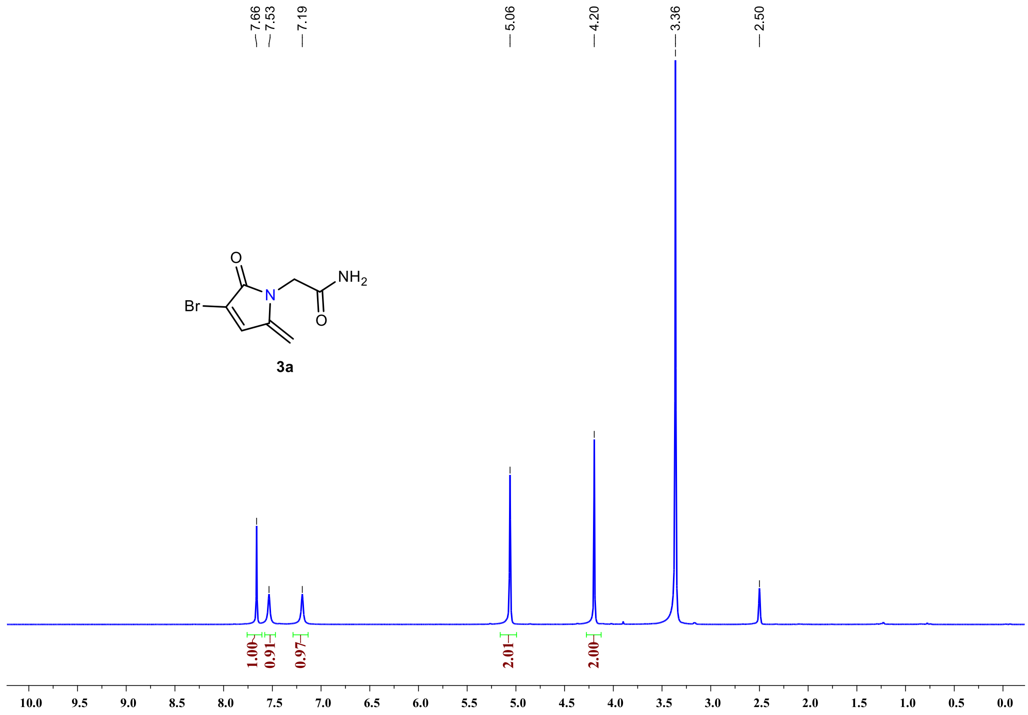

(B)

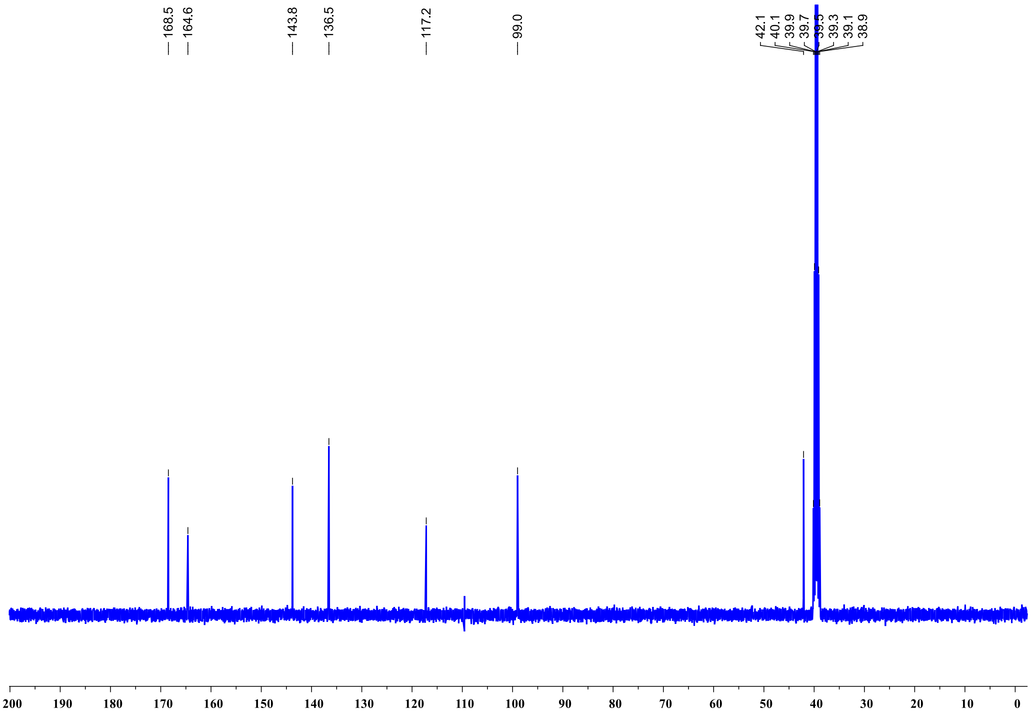

Supplementary Figure 3. NMR spectra of **3a**. (A) ^{1}H NMR spectrum of **3a**. (B) ^{13}C NMR spectrum of **3a**.

(A)

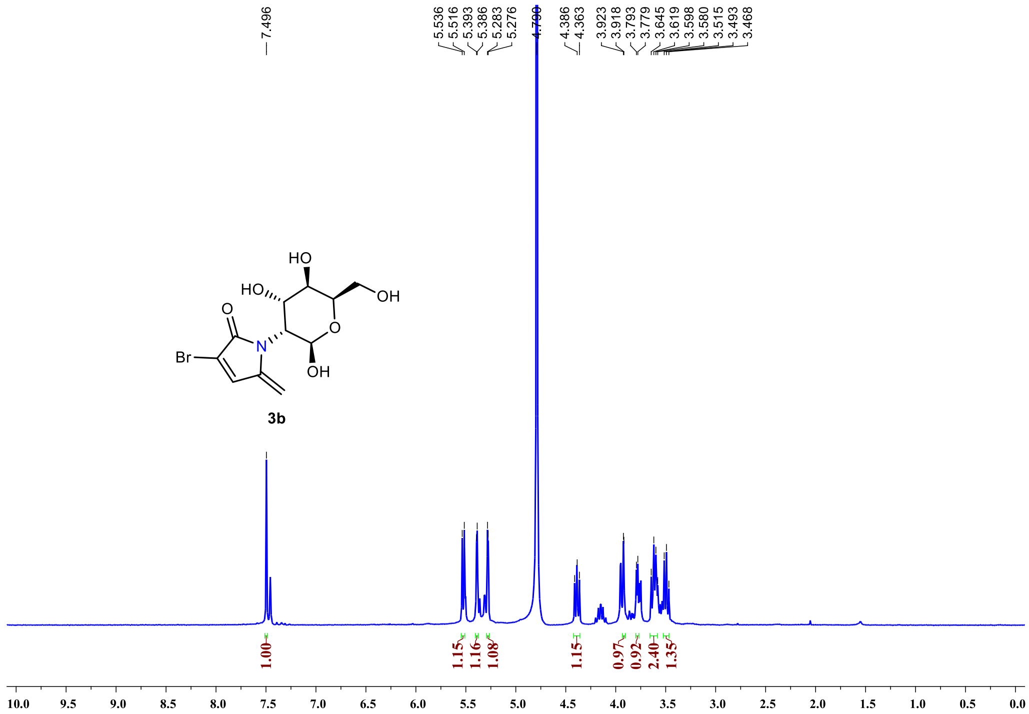

(B)

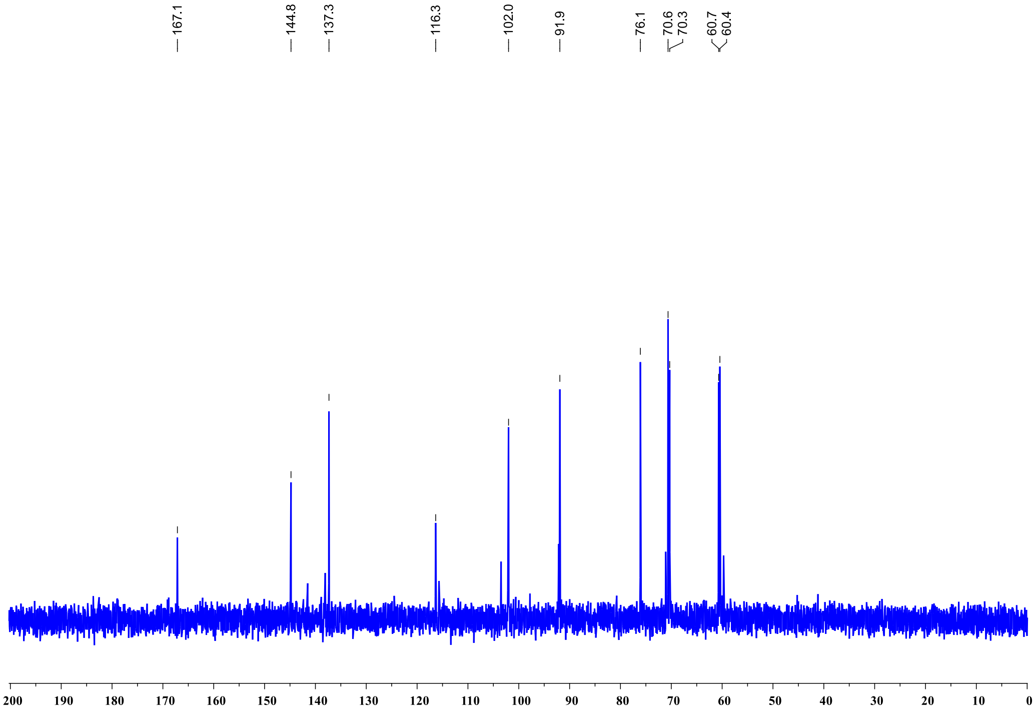

Supplementary Figure 4. NMR spectra of **3b**. (A) ^{1}H NMR spectrum of **3b**. (B) ^{13}C NMR spectrum of **3b**.

(A)

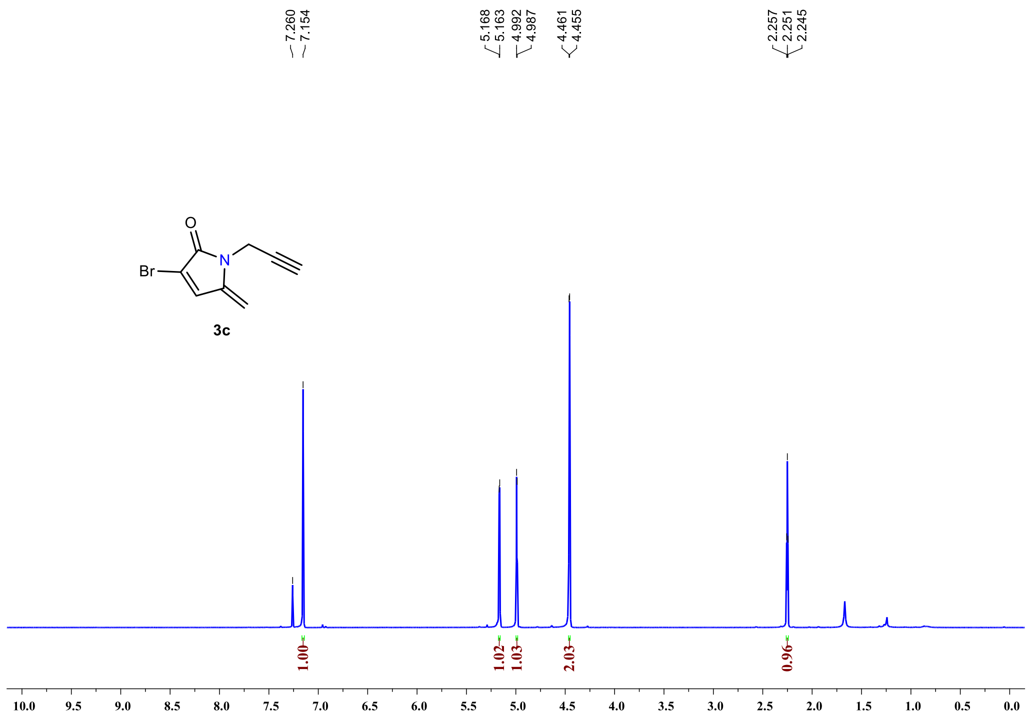

(B)

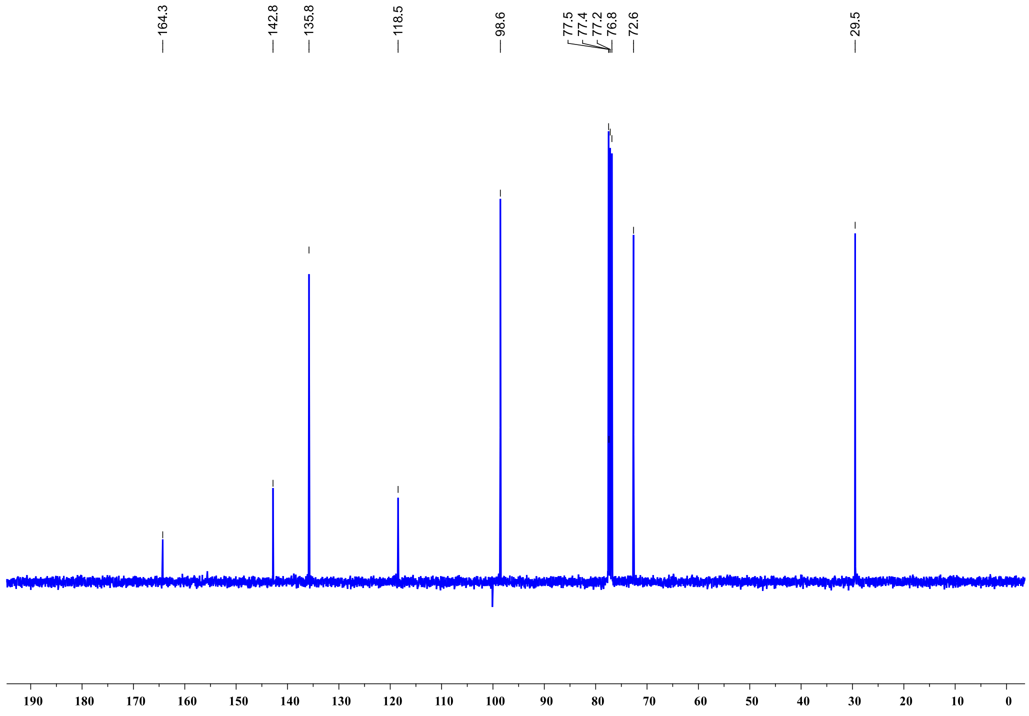

Supplementary Figure 5. NMR spectra of **3c**. (A) ^{1}H NMR spectrum of **3c**. (B) ^{13}C NMR spectrum of **3c**.

(A)

(B)

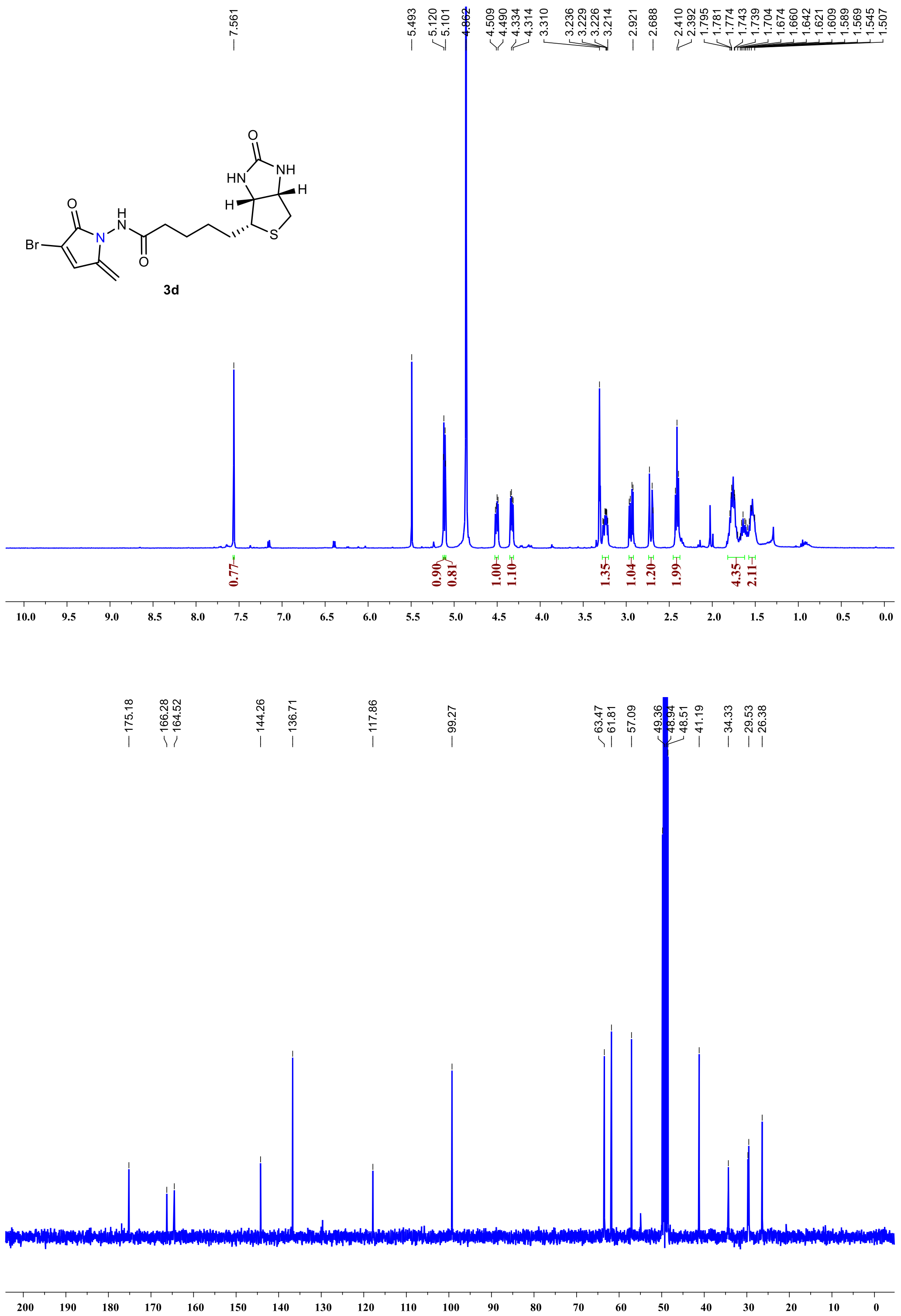

Supplementary Figure 6. NMR spectra of **3d**. (A) ^{1}H NMR spectrum of **3d**. (B) ^{13}C NMR spectrum of **3d**.

(A)

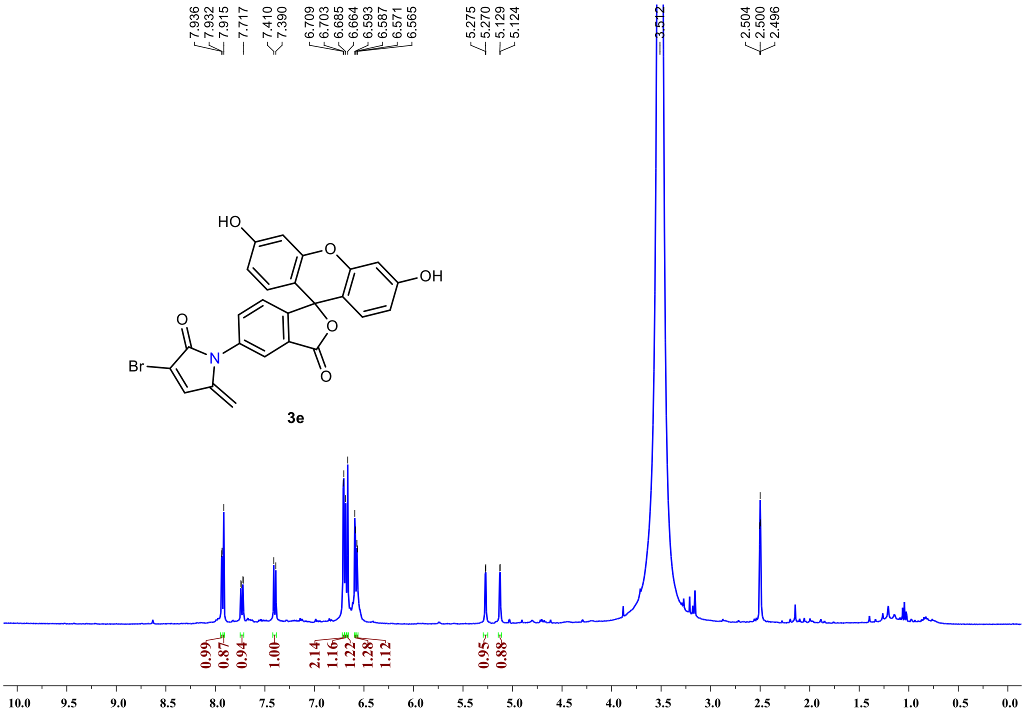

(B)

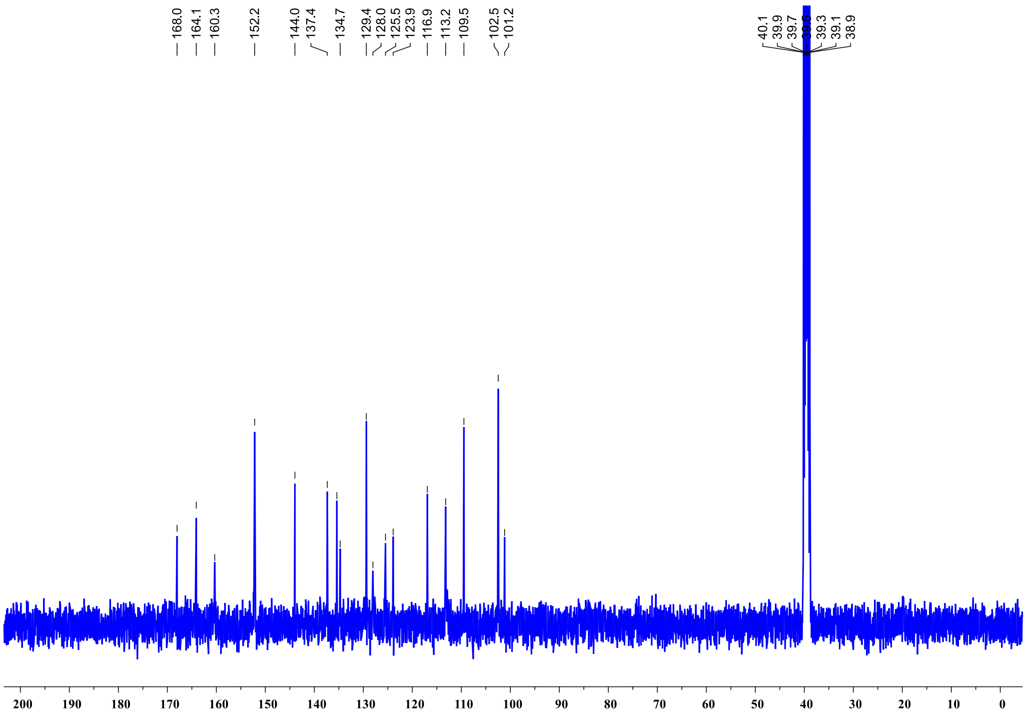

Supplementary Figure 7. NMR spectra of **3e**. (A) ^{1}H NMR spectrum of **3e**. (B) ^{13}C NMR spectrum of **3e**.

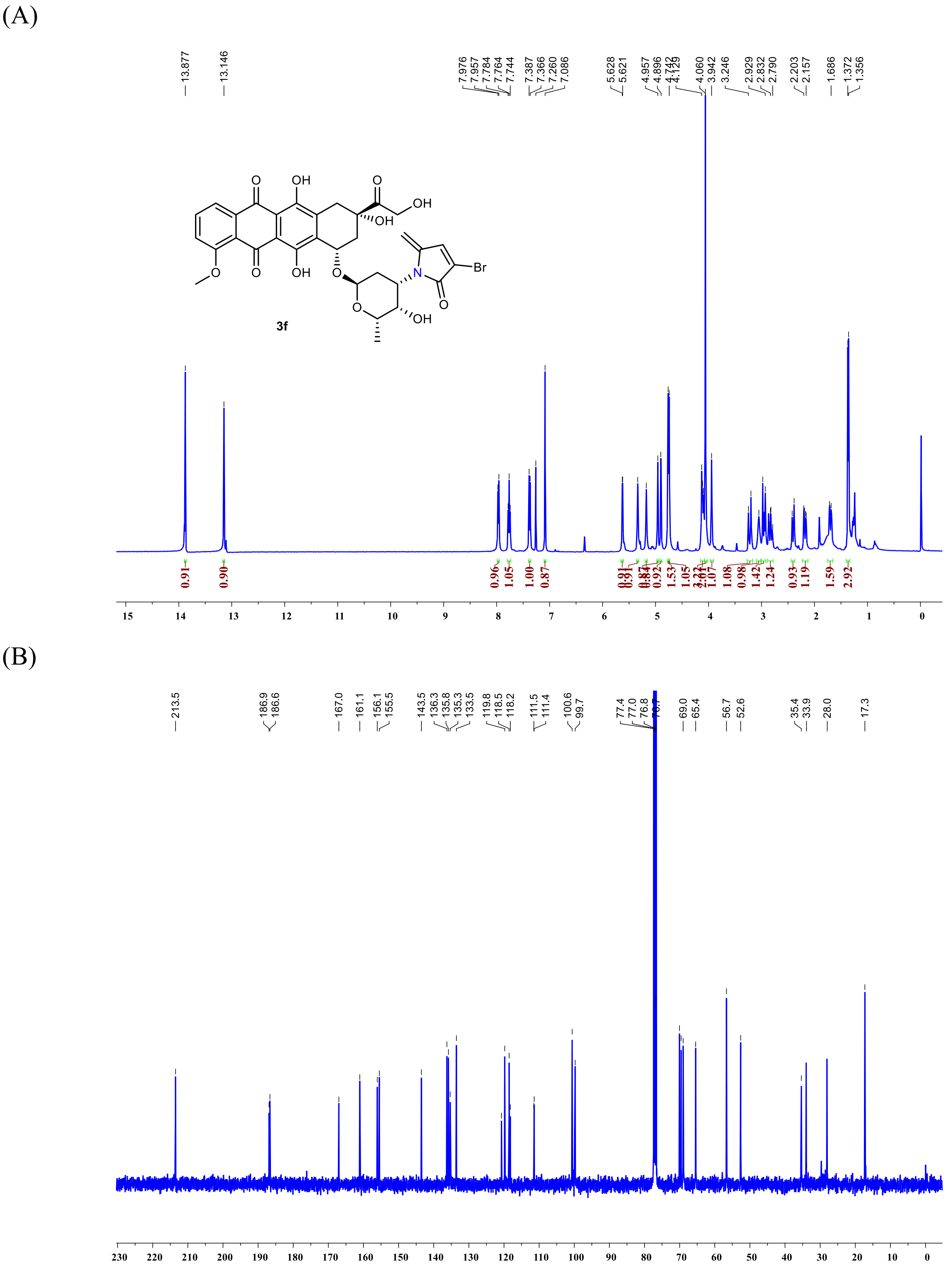

Supplementary Figure 8. NMR spectra of **3f**. (A) ^{1}H NMR spectrum of **3f**. (B) ^{13}C NMR spectrum of **3f**.

(A)

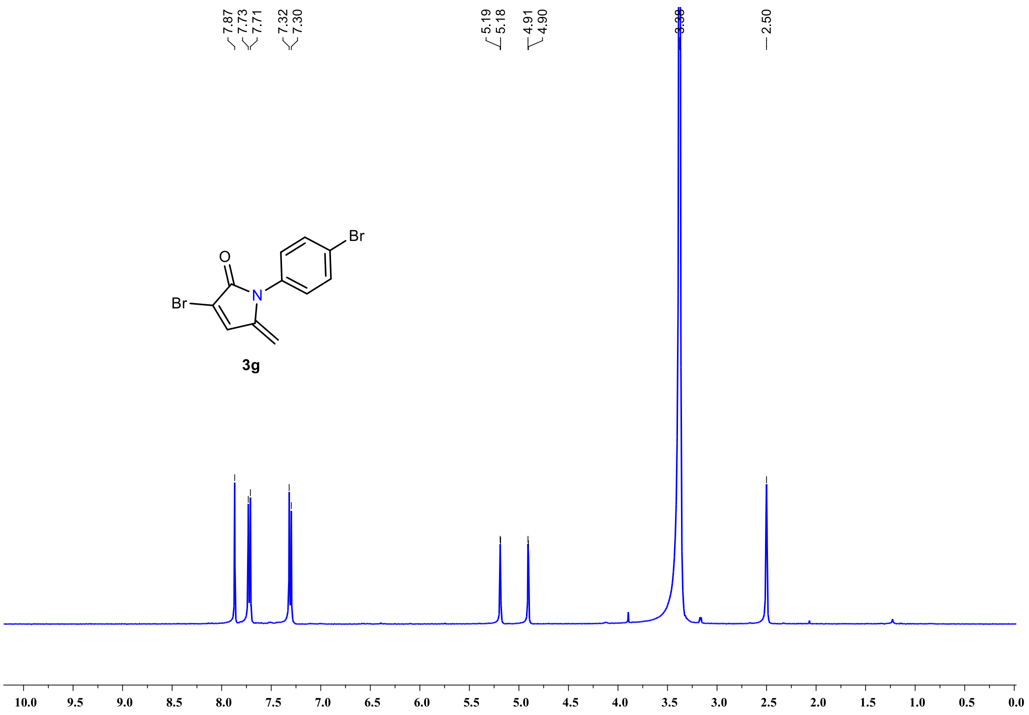

(B)

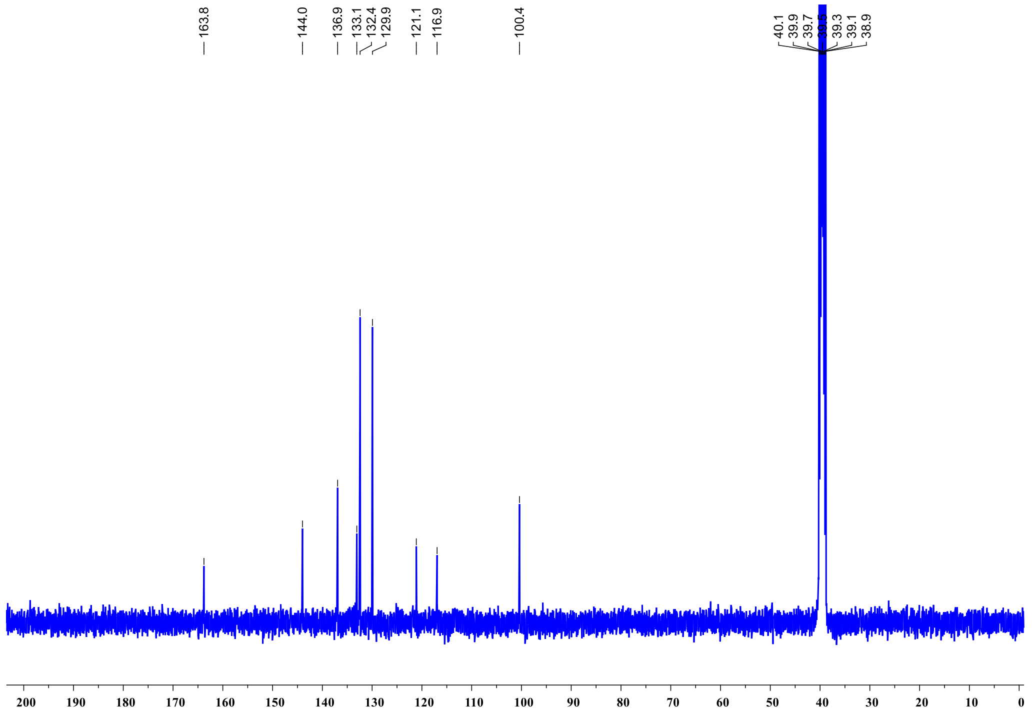

Supplementary Figure 9. NMR spectra of **3g**. (A) ^{1}H NMR spectrum of **3g**. (B) ^{13}C NMR spectrum of **3g**.

(A)

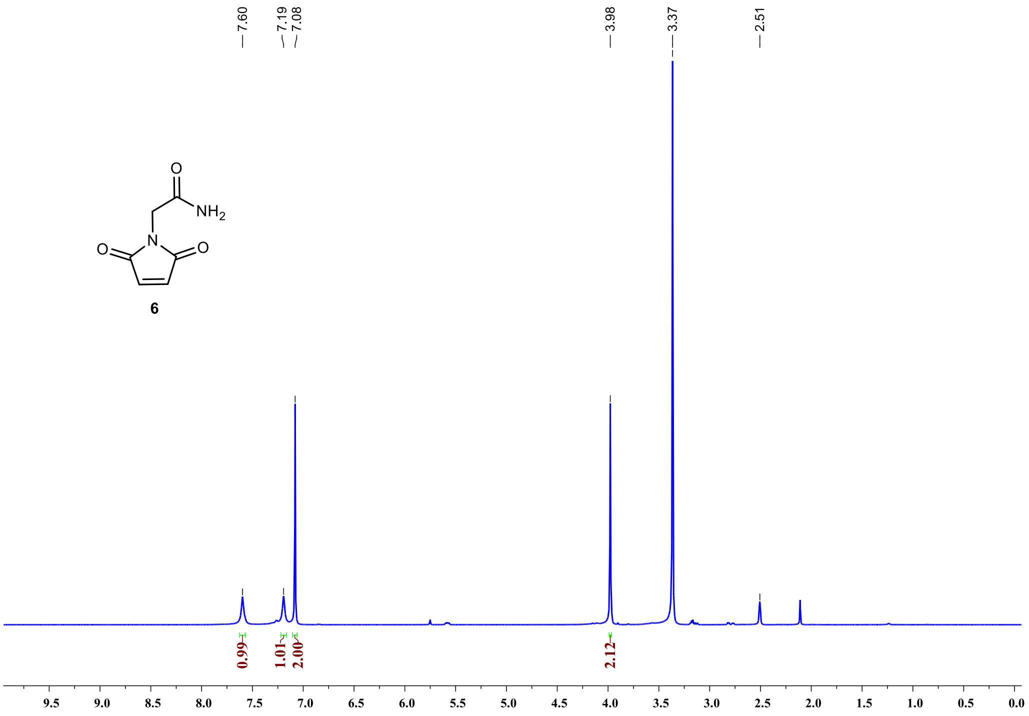

(B)

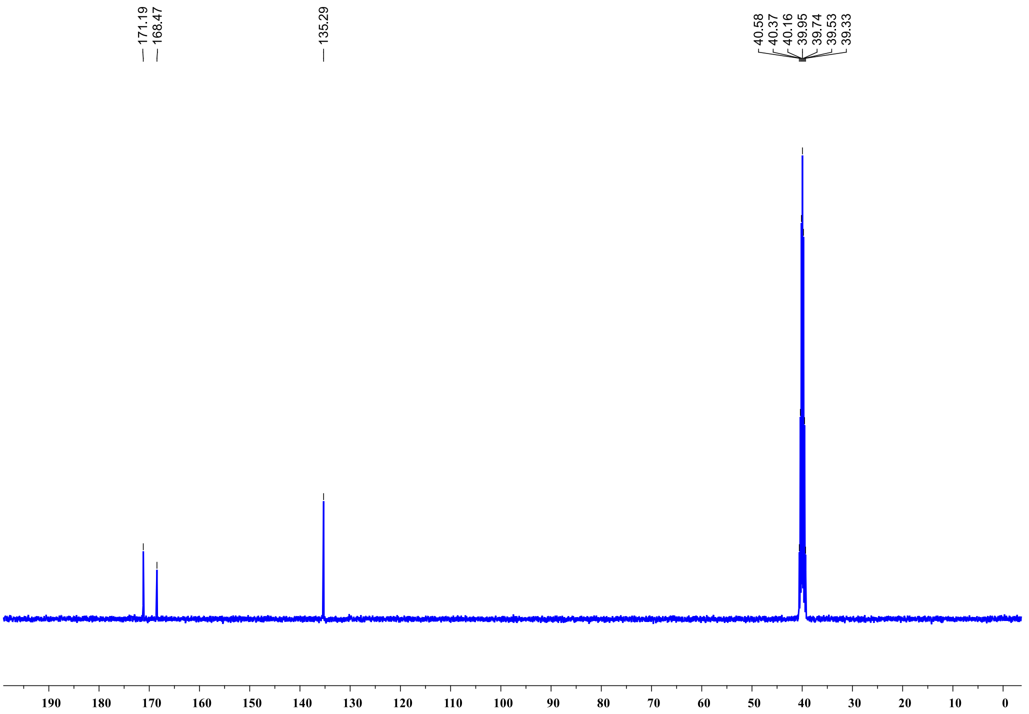

Supplementary Figure 10. NMR spectra of **6**. (A) ^{1}H NMR spectrum of **6**. (B) ^{13}C NMR spectrum of **6**.

(A)

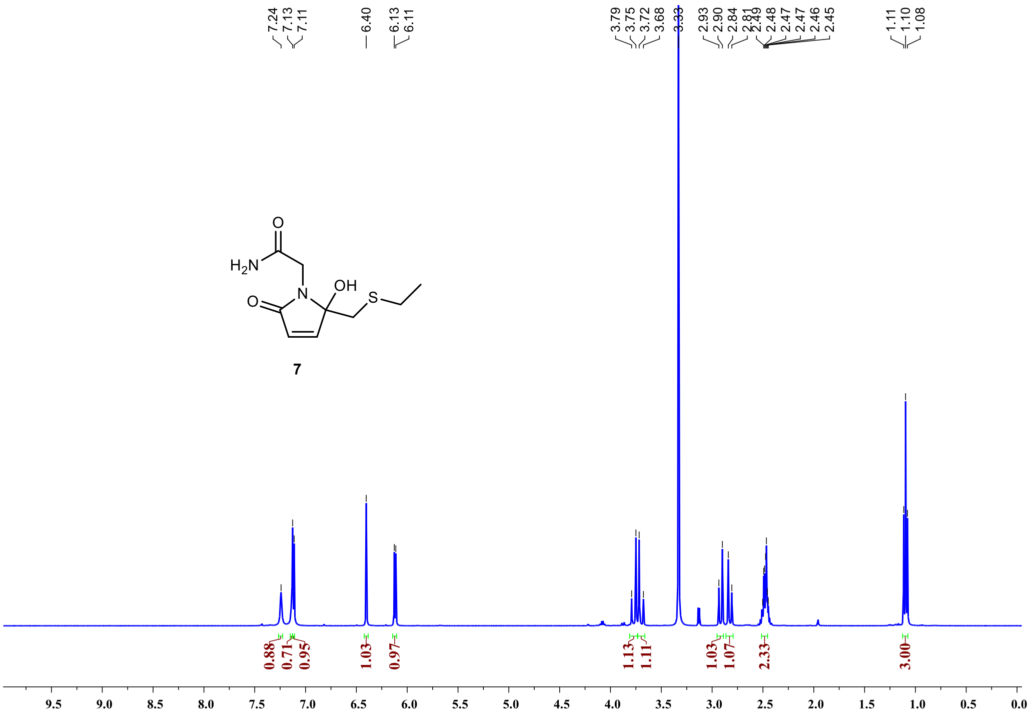

(B)

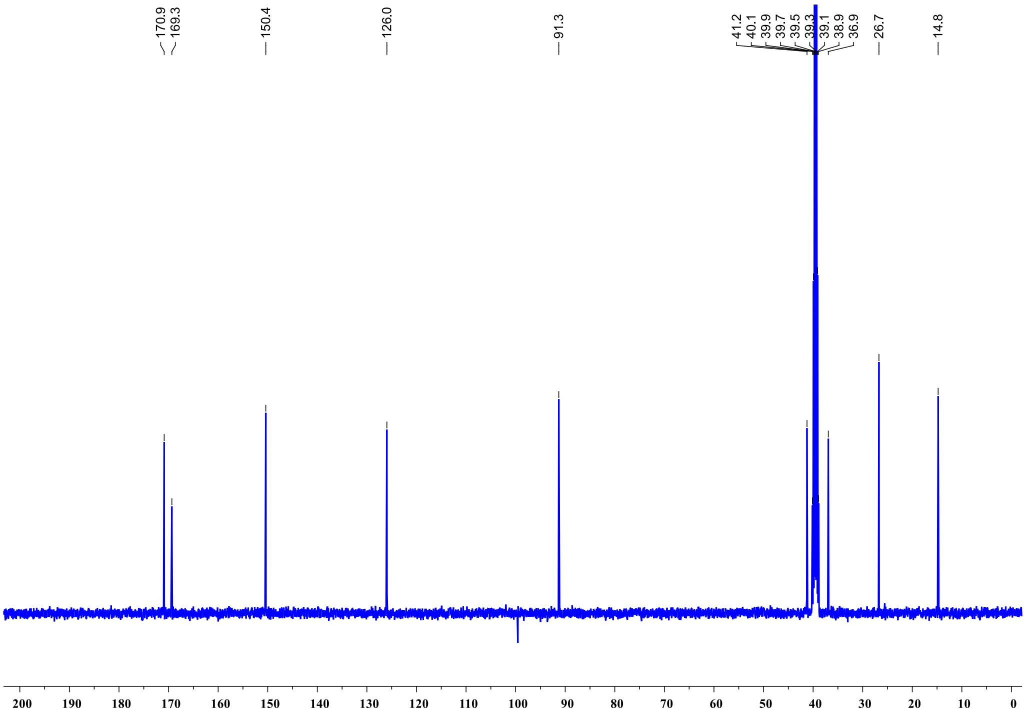

Supplementary Figure 11. NMR spectra of **7**. (A) ^{1}H NMR spectrum of **7**. (B) ^{13}C NMR spectrum of **7**.

(A)

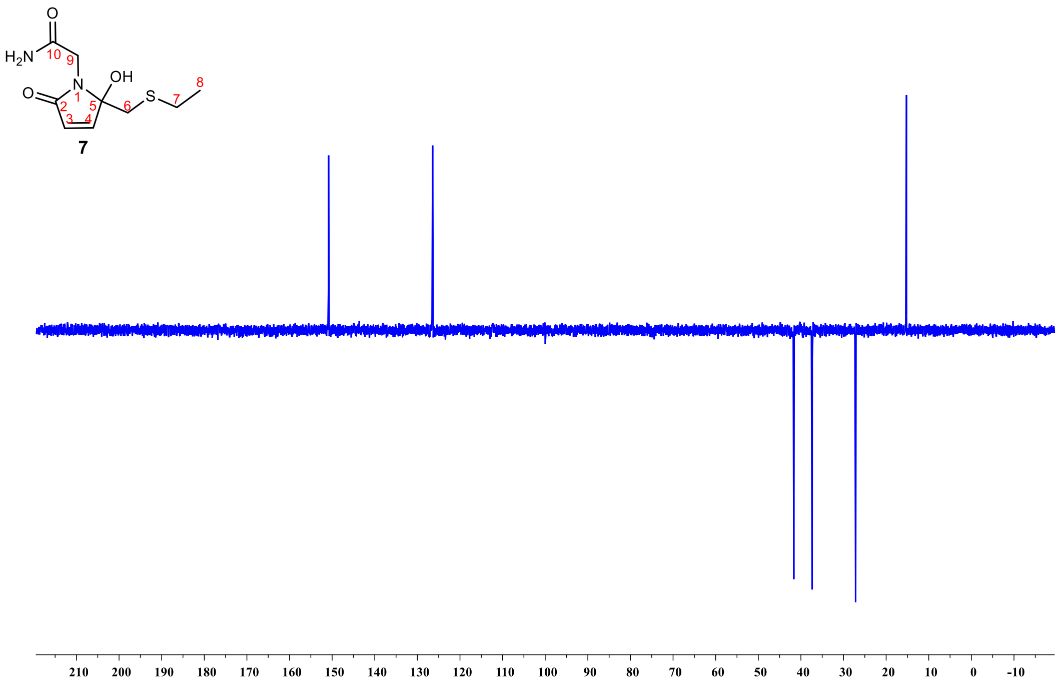

(B)

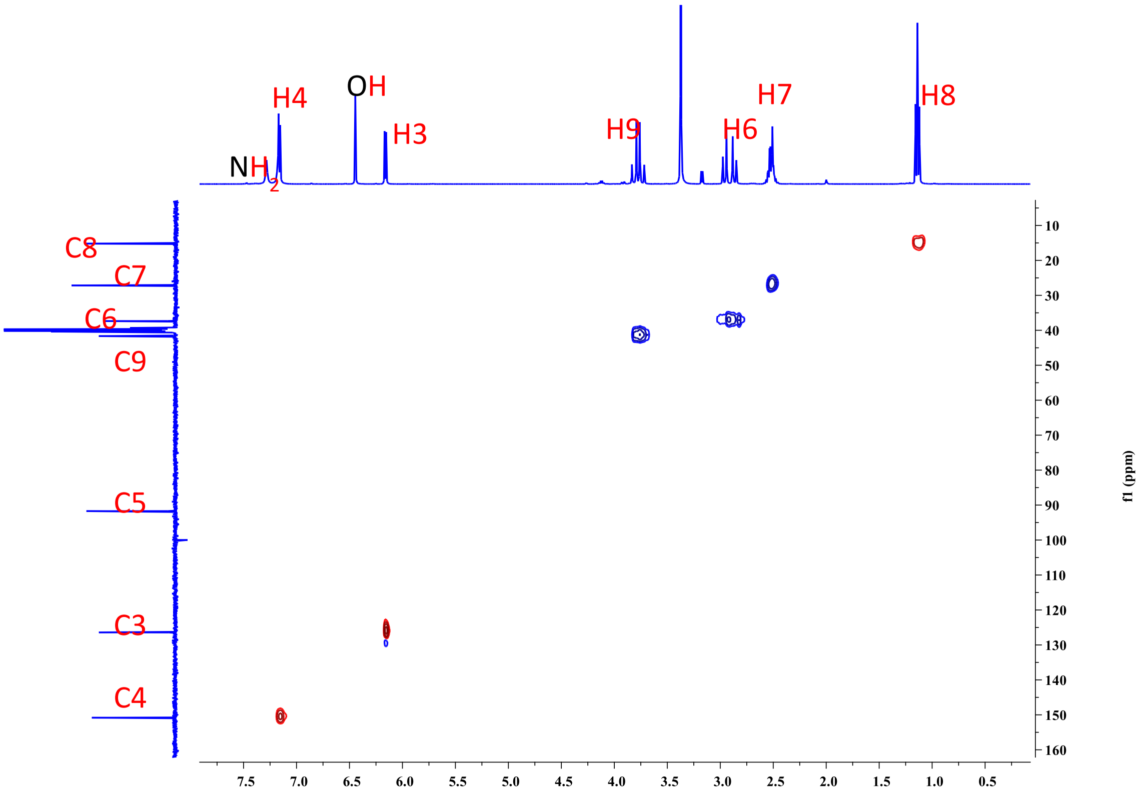

Supplementary Figure 12. NMR spectra of **7**. (A) DEPT 135 NMR spectrum of **7**. (B) HSQC NMR spectrum of **7**.

(A)

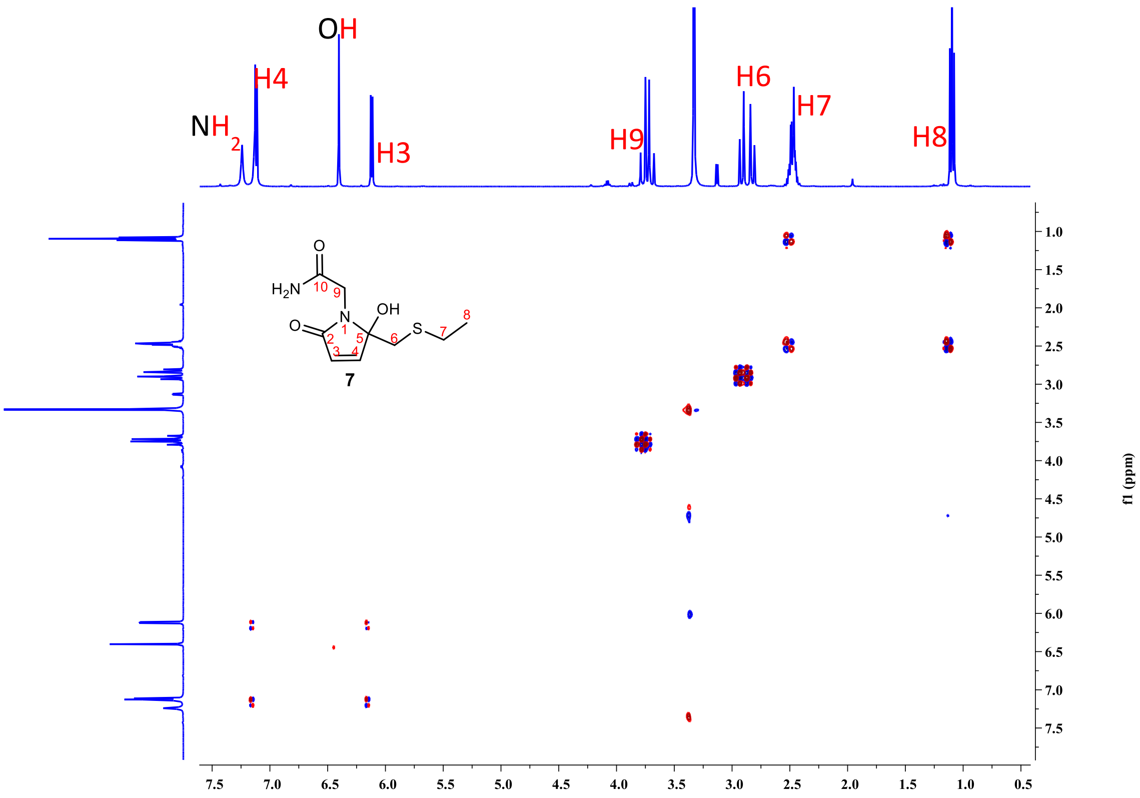

(B)

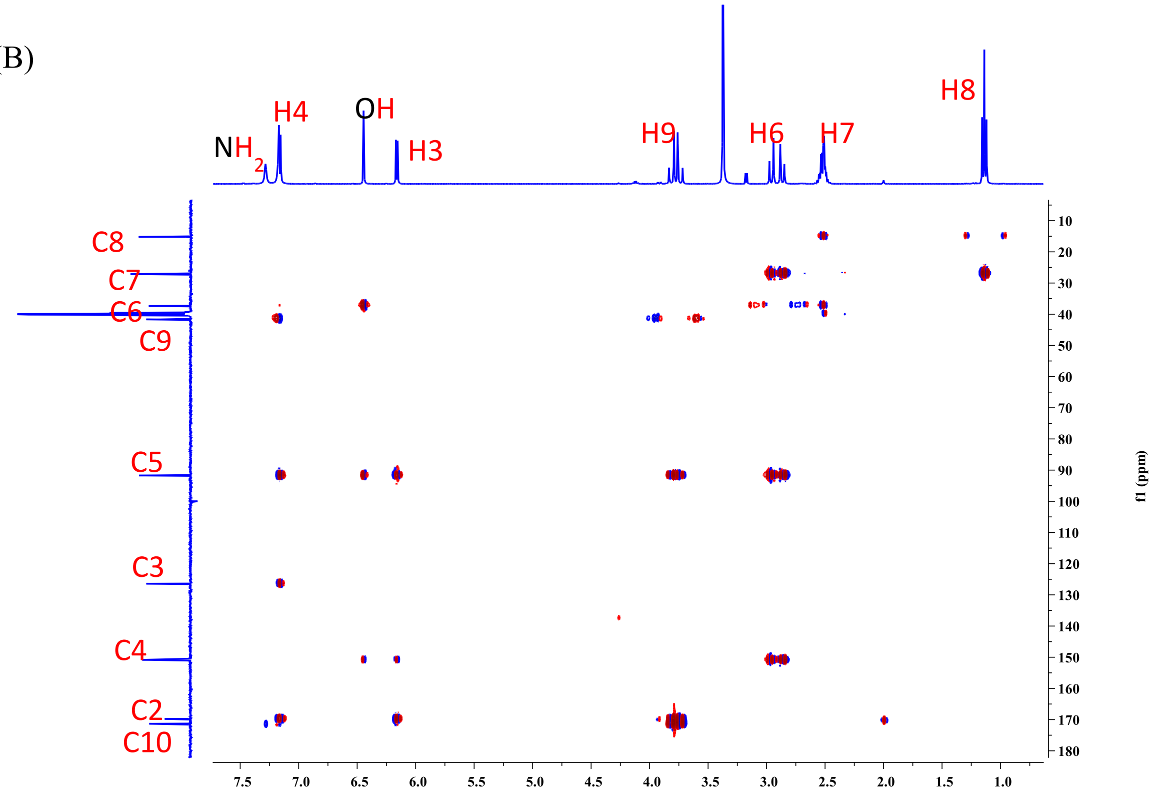

Supplementary Figure 13. NMR spectra of **7**. (A) COSY NMR spectrum of **7**. (B) HMBC NMR spectrum of **7**.

(A)

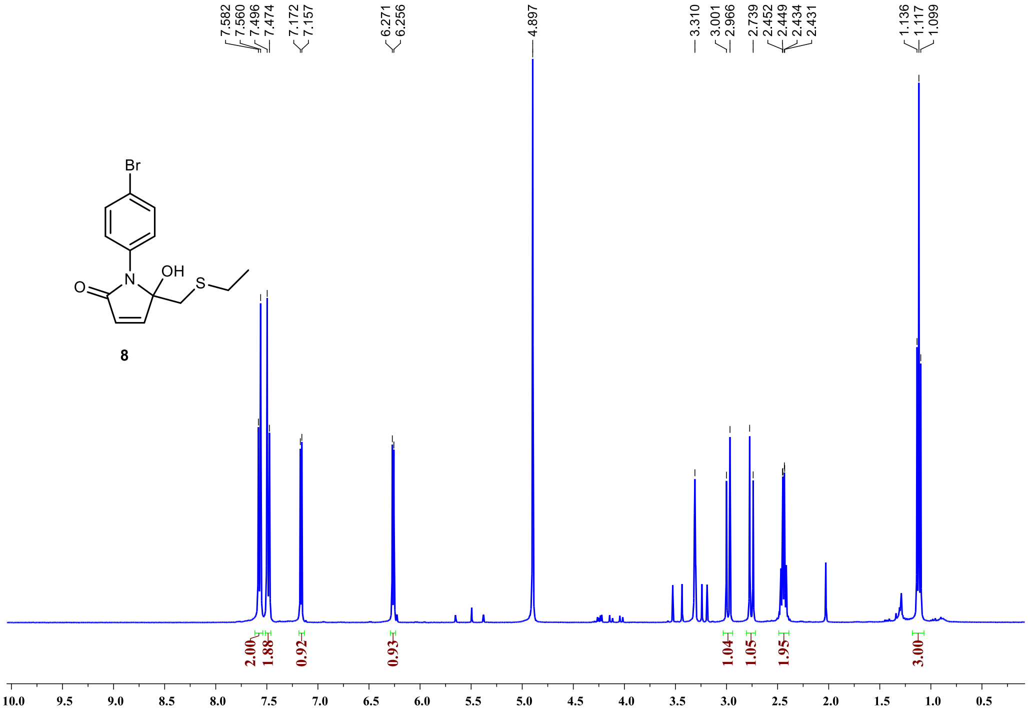

(B)

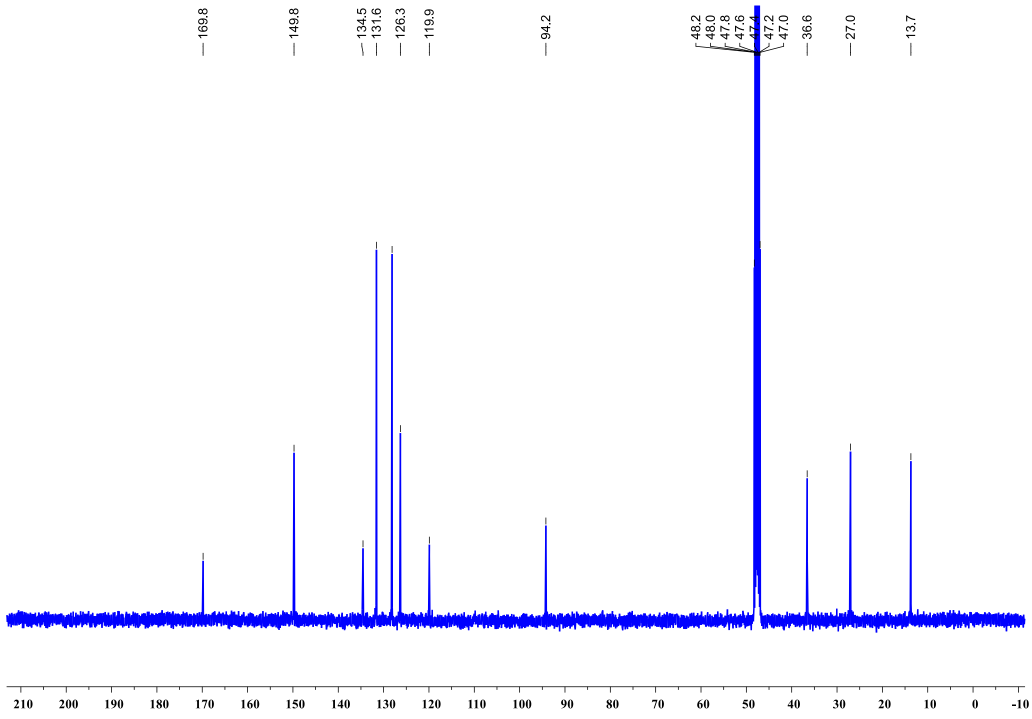

Supplementary Figure 14. NMR spectra of **8**. (A) ^{1}H NMR spectrum of **8**. (B) ^{13}C NMR spectrum of **8**.

(A)

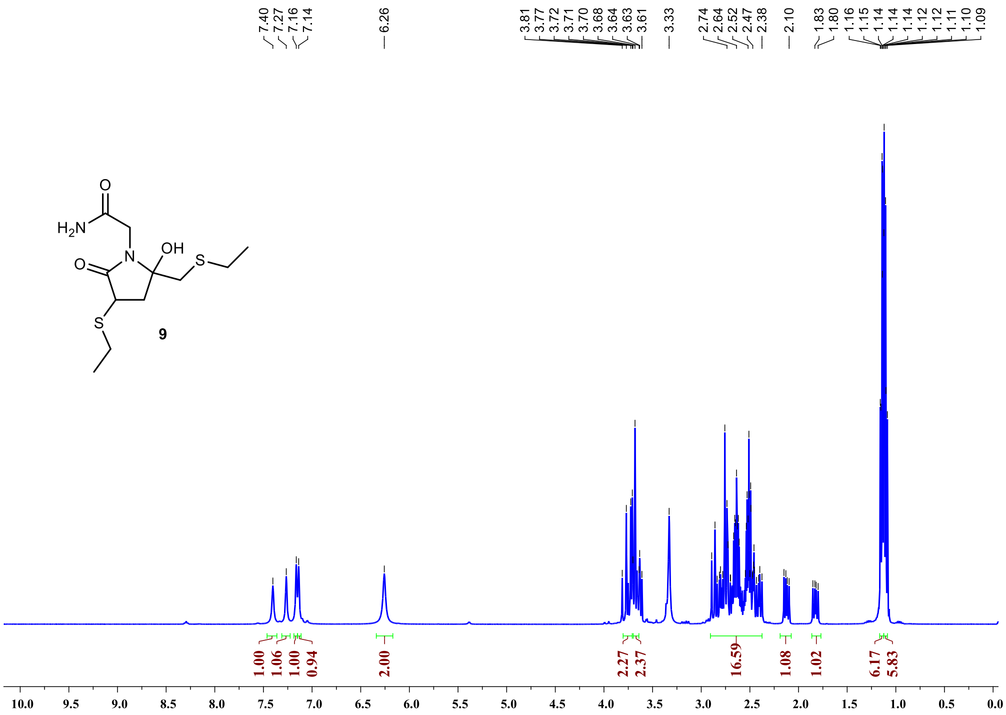

(B)

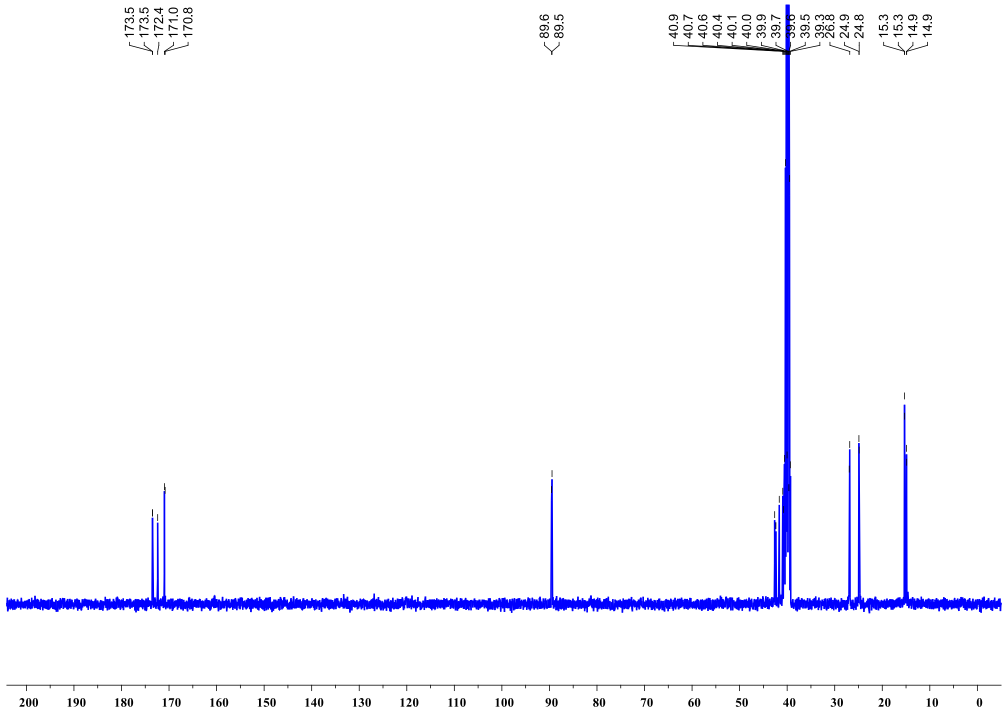

Supplementary Figure 15. NMR spectra of **9**. (A) ^{1}H NMR spectrum of **9**. (B) ^{13}C NMR spectrum of **9**.

(A)

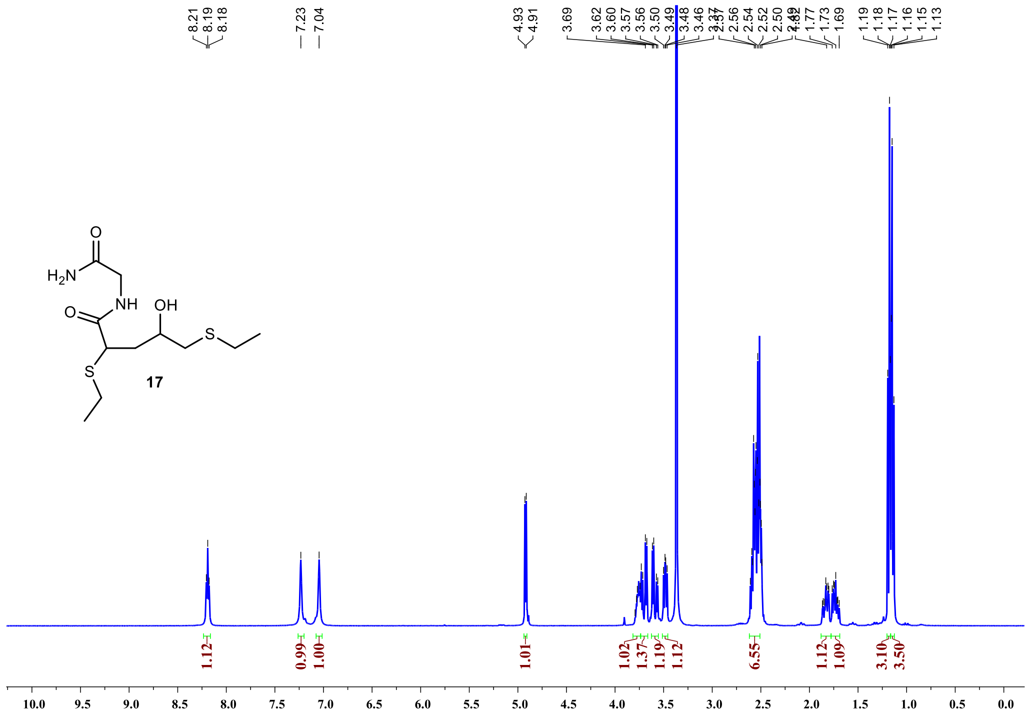

(B)

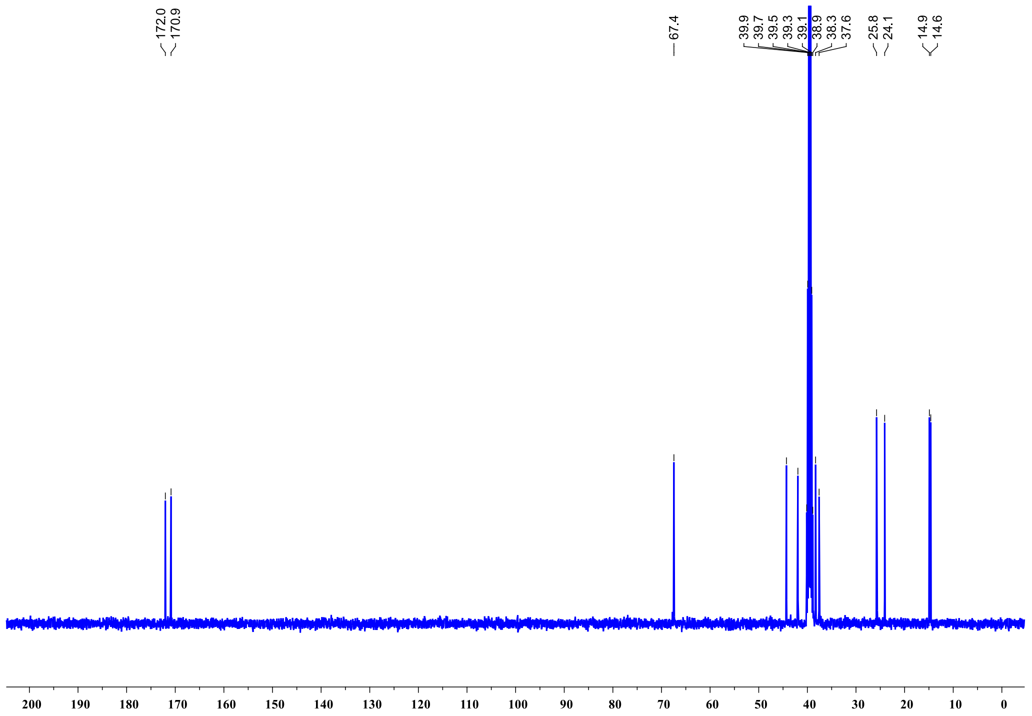

Supplementary Figure 16. NMR spectra of **17**. (A) ^{1}H NMR spectrum of **17**. (B) ^{13}C NMR spectrum of **17**.

(A)

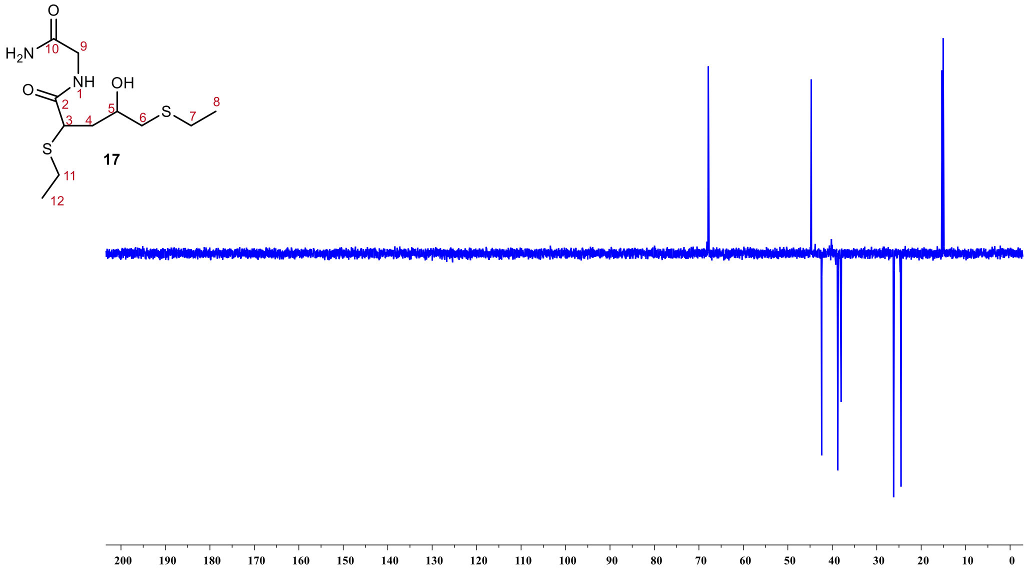

(B)

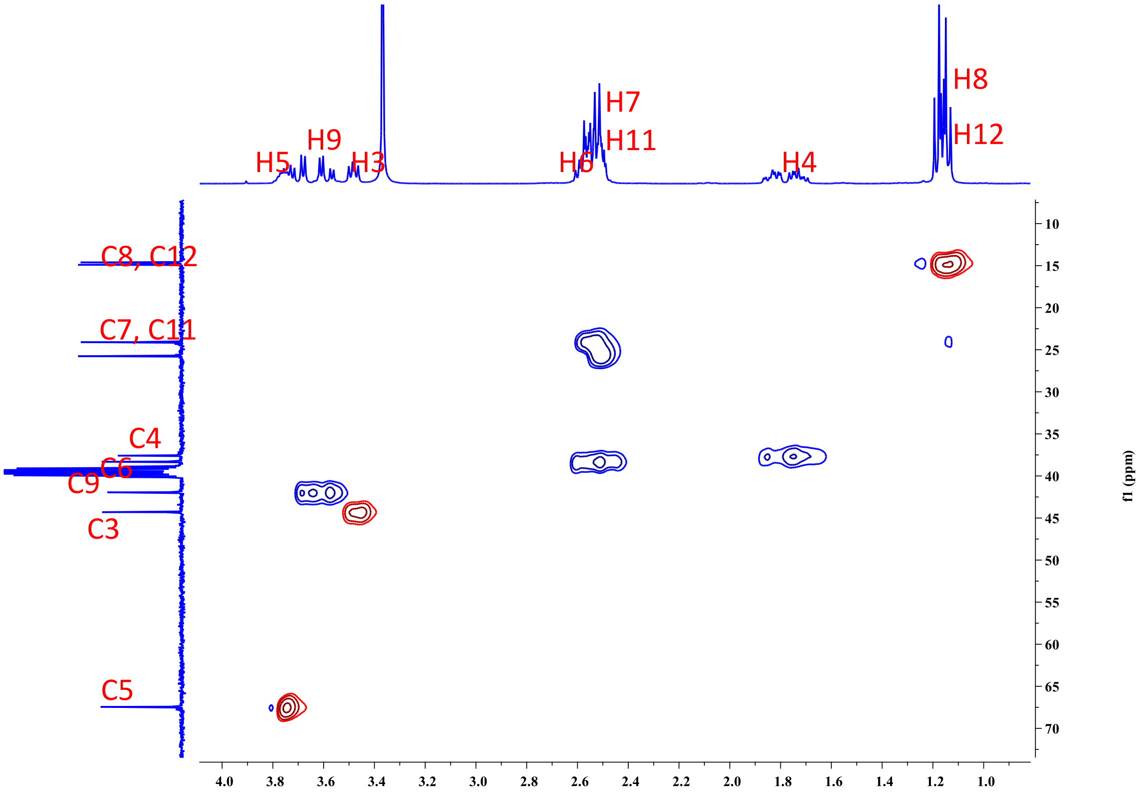

Supplementary Figure 17. NMR spectra of **17**. (A) DEPT 135 NMR spectrum of **17**. (B) HSQC NMR spectrum of **17**.

(A)

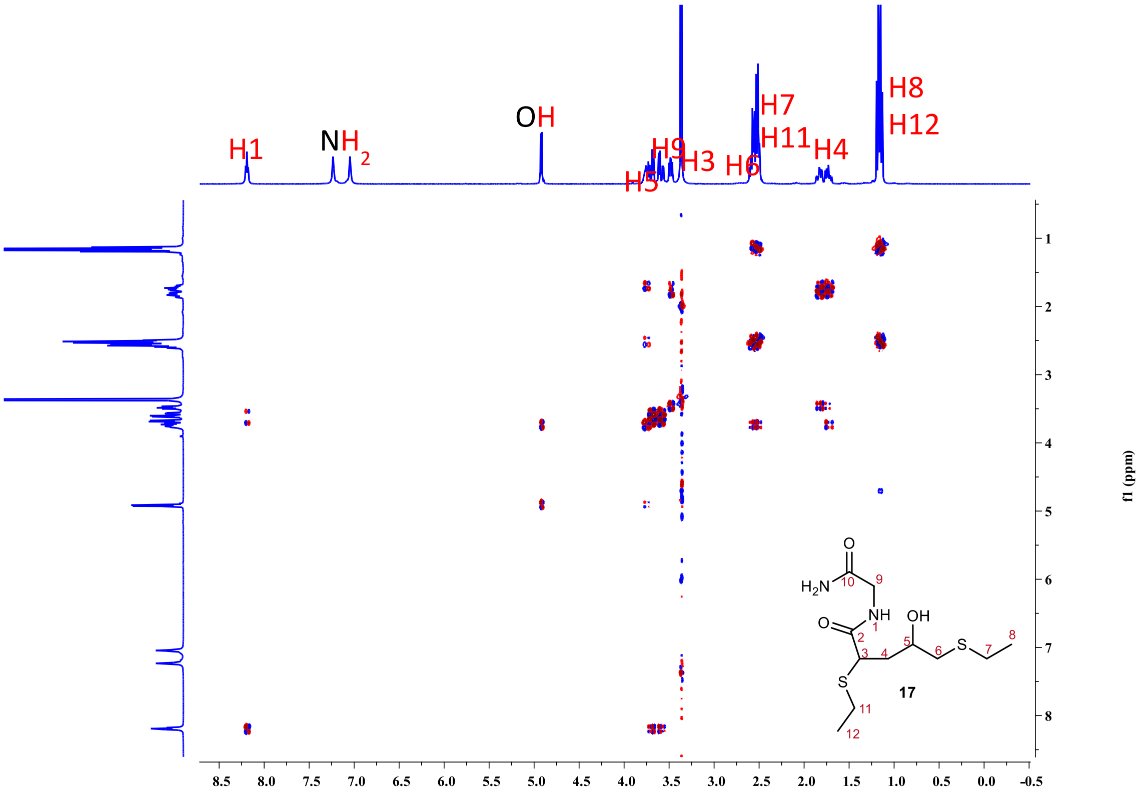

(B)

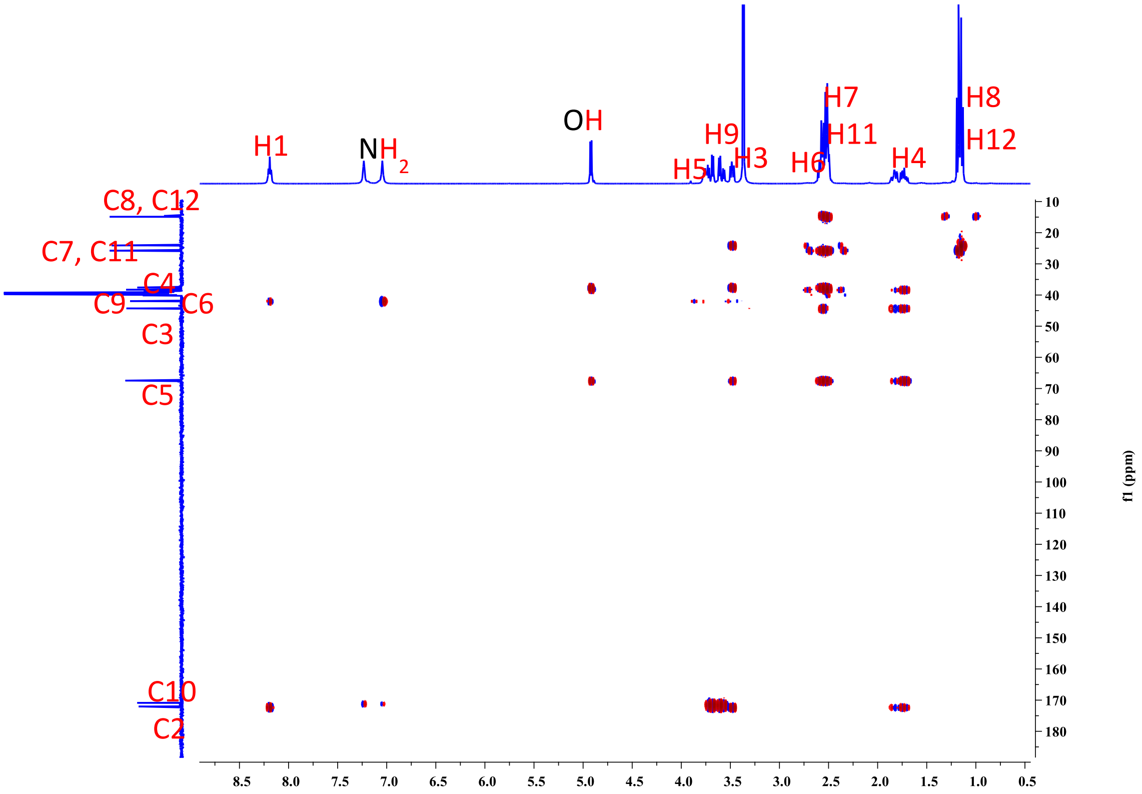

Supplementary Figure 18. NMR spectra of **17**. (A) COSY NMR spectrum of **17**. (B) HMBC NMR spectrum of **17**.

(A)

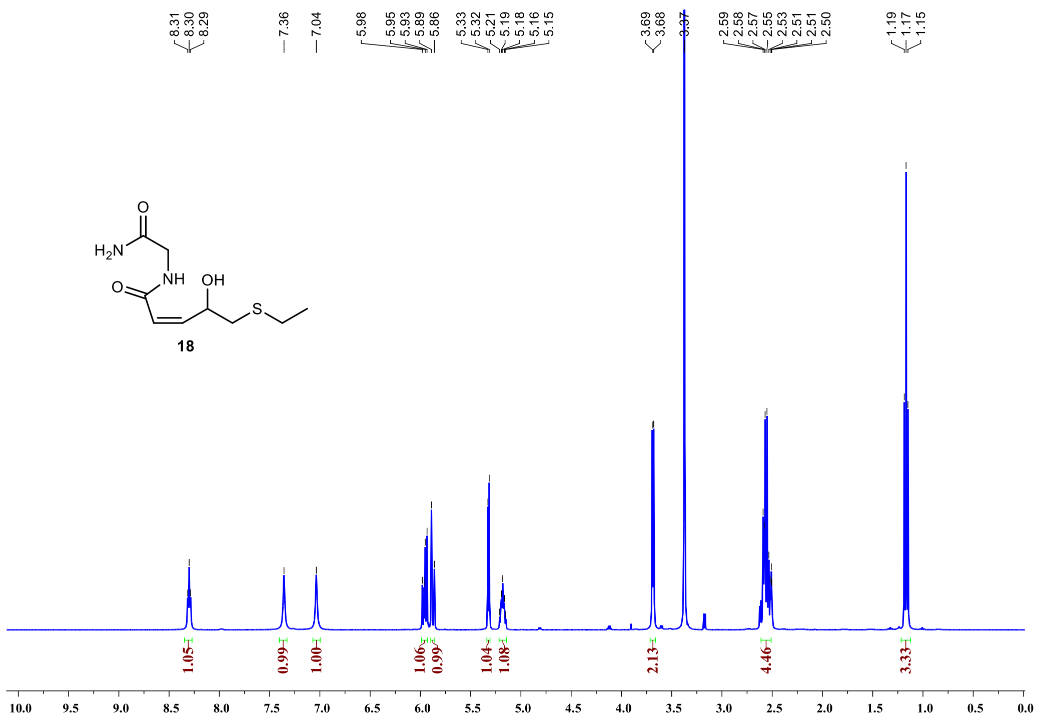

(B)

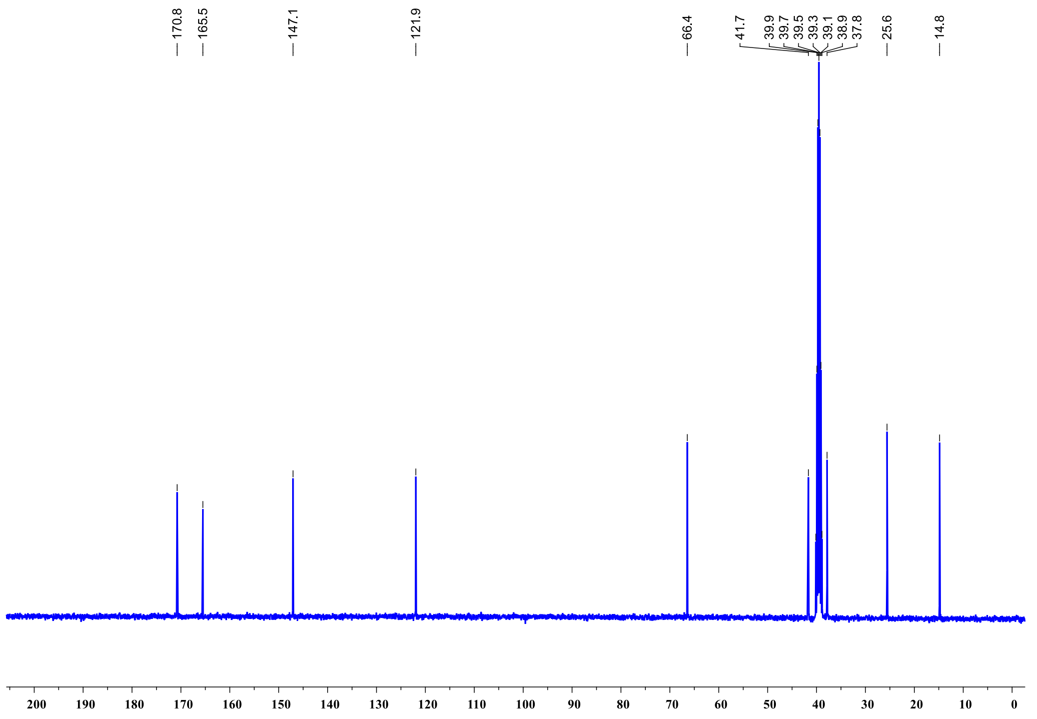

Supplementary Figure 19. NMR spectra of **18**. (A) ^{1}H NMR spectrum of **18**. (B) ^{13}C NMR spectrum of **18**.

(A)

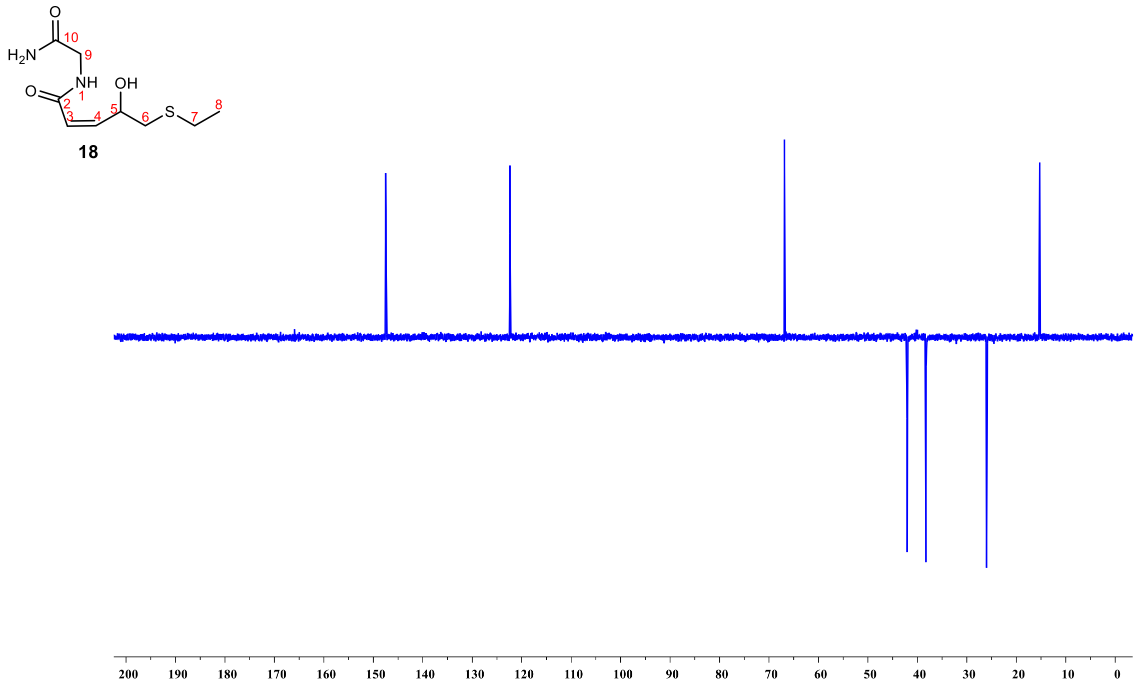

(B)

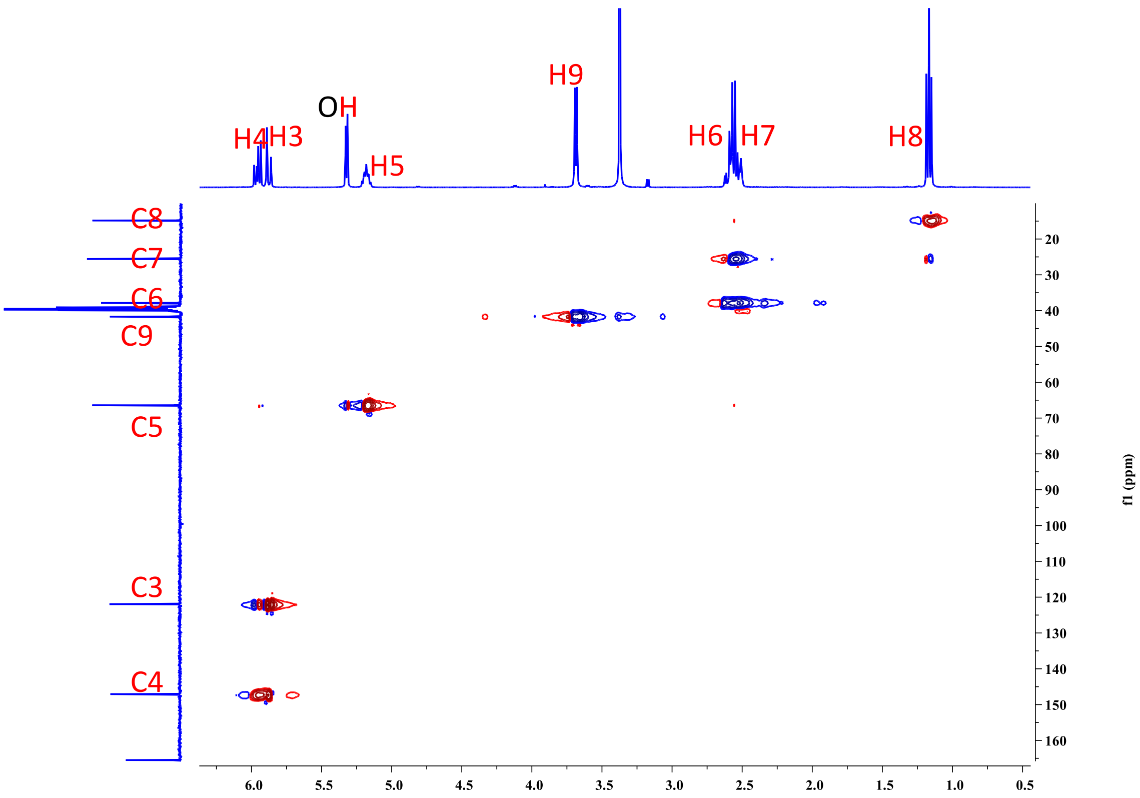

Supplementary Figure 20. NMR spectra of **18**. (A) DEPT 135 NMR spectrum of **18**. (B) HSQC NMR spectrum of **18**.

(A)

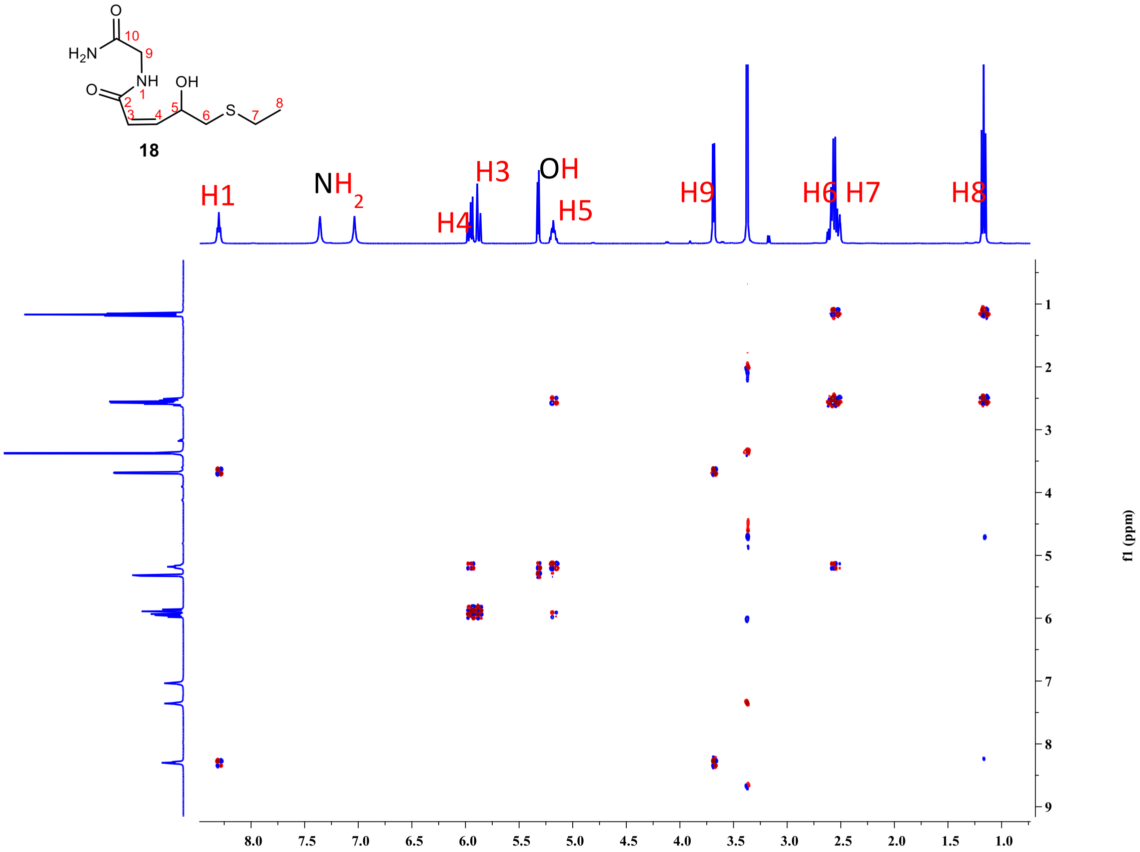

(B)

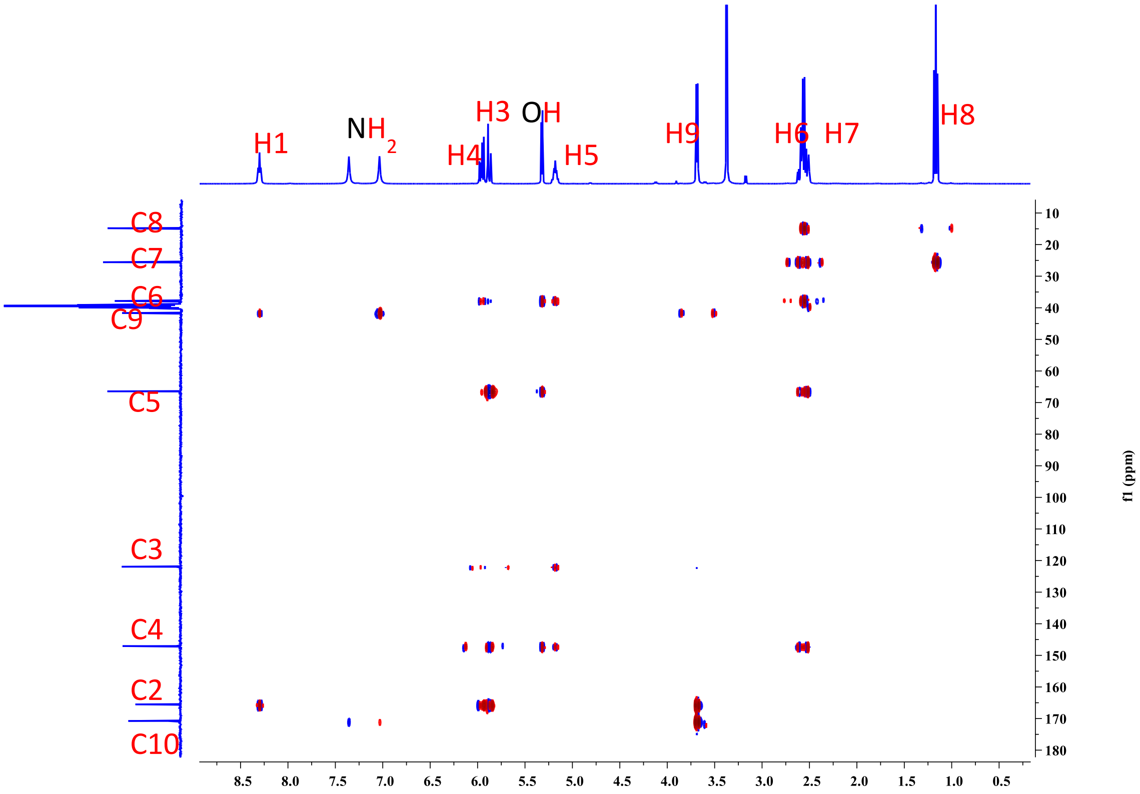

Supplementary Figure 21. NMR spectra of **18**. (A) COSY NMR spectrum of **18**. (B) HMBC NMR spectrum of **18**.

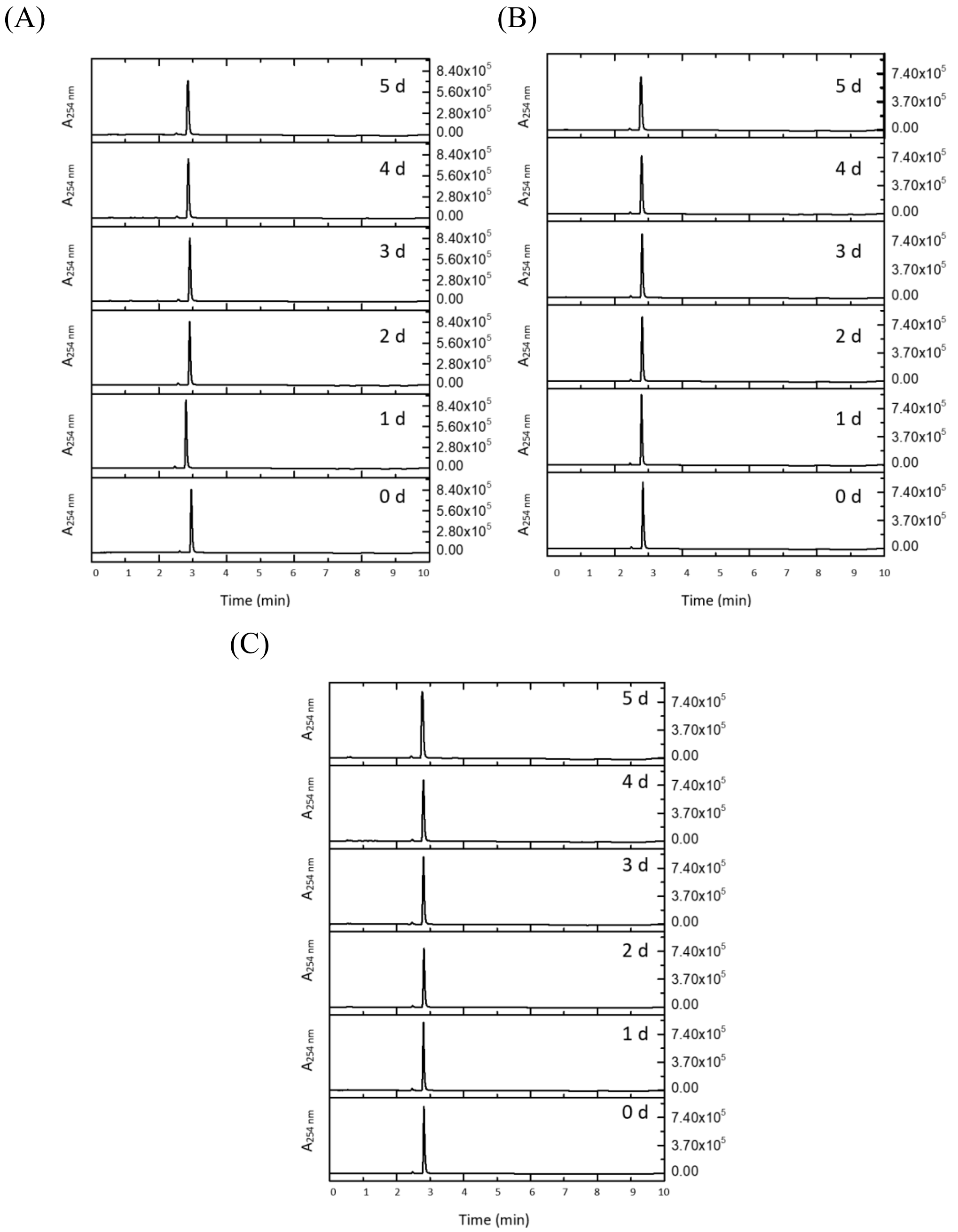

Supplementary Figure 22. UPLC analyses of the stability of **3a** in different conditions. (A) MES buffer, pH 6.0. (B) HEPES buffer, pH 7.5. (C) Sodium borate buffer, pH 9.5.

Reaction conditions: A solution of **3a** (storage solution: water contained 5% DMSO, final concentration 2 mM) in an appropriate buffer (MES, HEPES and sodium borate buffer were used for pH 6.0, 7.5, 9.5 respectively, final concentration 20 mM, 100 mM NaCl) was incubated at 37 ºC. 1 μL of reaction mixture was taken at appropriate intervals and analyzed by UPLC-MS. UPLC condition (column II): 0-6 min, B: 5-30%.

(A)

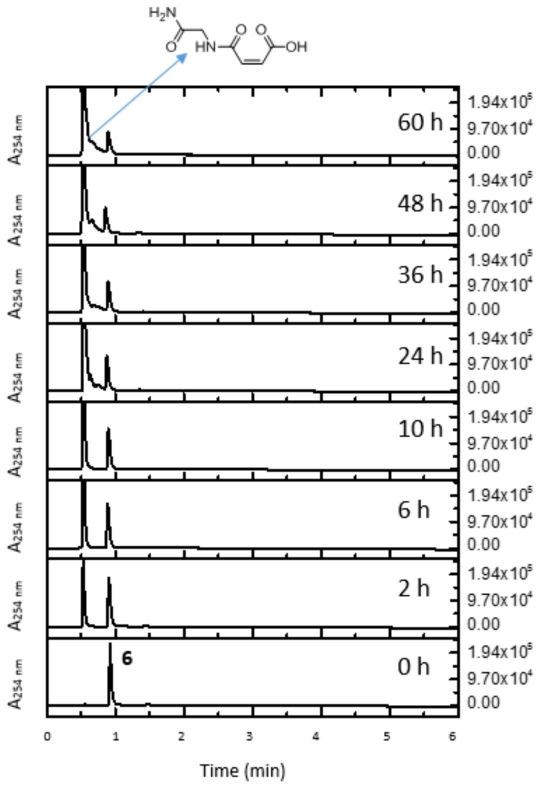

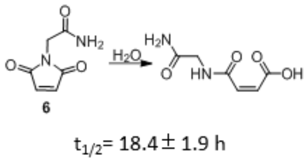

(B)

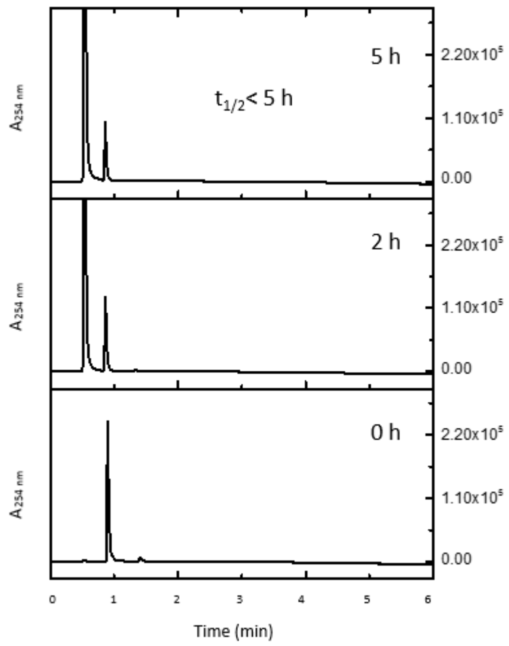

(C)

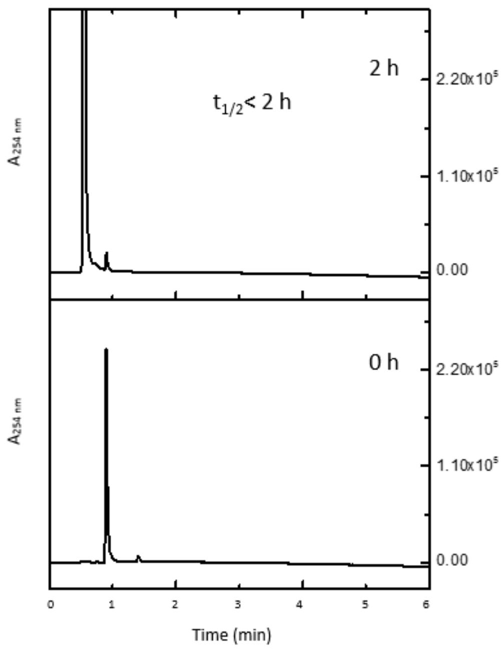

Supplementary Figure 23. UPLC analyses of the stability of **6** in different conditions. (A) MES buffer, pH 6.0. (B) HEPES buffer, pH 7.5. (C) Sodium borate buffer, pH 9.5.

Reaction conditions: A solution of **6** (final concentration 2 mM) in an appropriate buffer (MES, HEPES and sodium borate buffer were used for pH 6.0, 7.5, 9.5 respectively, final concentration 20 mM, 100 mM NaCl) was incubated at 37 ºC. 1 µL of reaction mixture was taken at appropriate intervals and analyzed by UPLC-MS. UPLC condition (column II): 0-6 min, B: 5-30%.

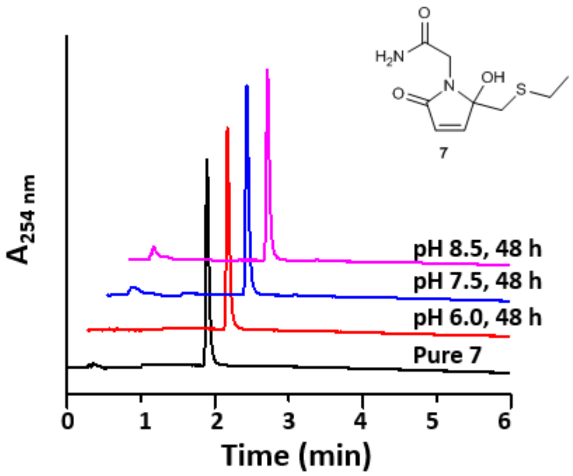

Supplementary Figure 24. UPLC analyses of the stability of **7** in different conditions.

Reaction conditions: A solution of **7** (final concentration 2 mM) in an appropriate buffer (MES, HEPES and HEPPSO buffer were used for pH 6.0, 7.5, 8.5 respectively, final concentration 20 mM, 100 mM NaCl) was incubated at 37 ºC. 1 μL of reaction mixture was taken after 2 days and analyzed by UPLC-MS. UPLC condition (column I): 0-6 min, B: 5-20%.

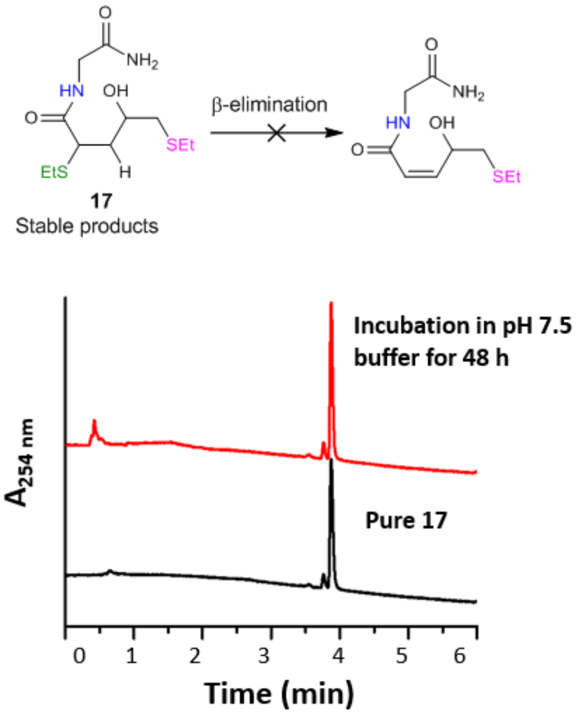

Supplementary Figure 25. UPLC analyses of the stability of **17** in HEPES buffer (pH = 7.5).

Reaction conditions: A solution of **17** (final concentration 5 mM) in HEPES (20 mM, pH 7.5, 100 mM NaCl) incubation at 37 ºC.1 µL of reaction mixture was taken after 2 days and analyzed by UPLC-MS. UPLC condition (column II): 0-6 min, B: 5-70%.

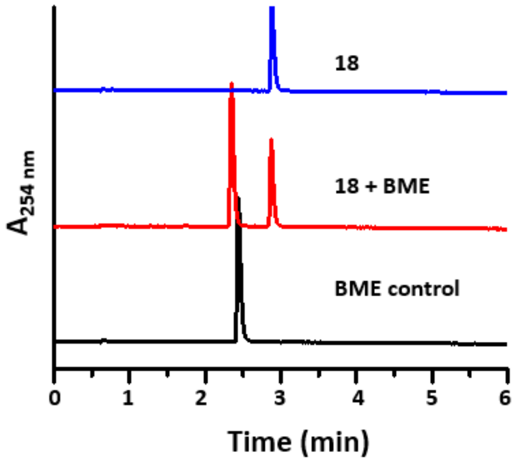

Supplementary Figure 26. UPLC analyses of the reaction of **18** with BME.

Reaction conditions: A solution of **18** (final concentration 2 mM) and BME (final concentration 40 mM, 20 equiv) in HEPES (20 mM, pH 7.5, 100 mM NaCl) incubation at 37 ºC.1 μL of reaction mixture was taken after 12 h and analyzed by UPLC-MS. UPLC condition (column II): 0-6 min, B: 5-30%.

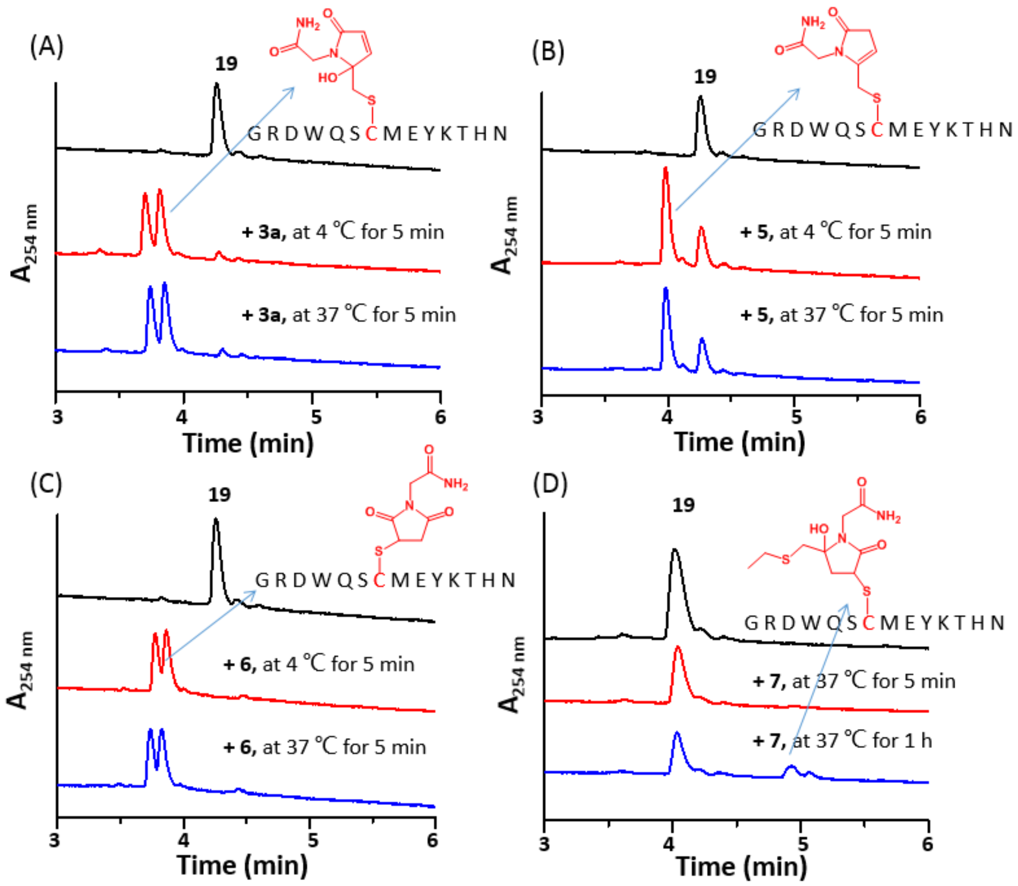

Supplementary Figure 27. UPLC analyses of the reactions of peptide **19** with 2 equiv of **3a**, **5**, **6** or **7**. (A) **3a**; (B) **5**; (C) **6**; (D) **7**.

Reaction condition: Peptide **19** (0.2 mM) and substrate **3a**, **5**, **6** or **7** (0.4 mM, 2 equiv) in HEPES (20 mM, pH 7.5, 100 mM NaCl) incubation at 37 °C or 4 °C for 5 min. For substrate **6**, the reaction time was extended to 1 h. 2 μL of reaction mixture were analyzed by UPLC-MS. UPLC condition (column I): 0-6 min, B: 5-20%, C: 10% keep constant.

(A) UPLC analysis of reactions of peptide **19** with 10 equiv of **3a**, **5**, **6**, or **7**.

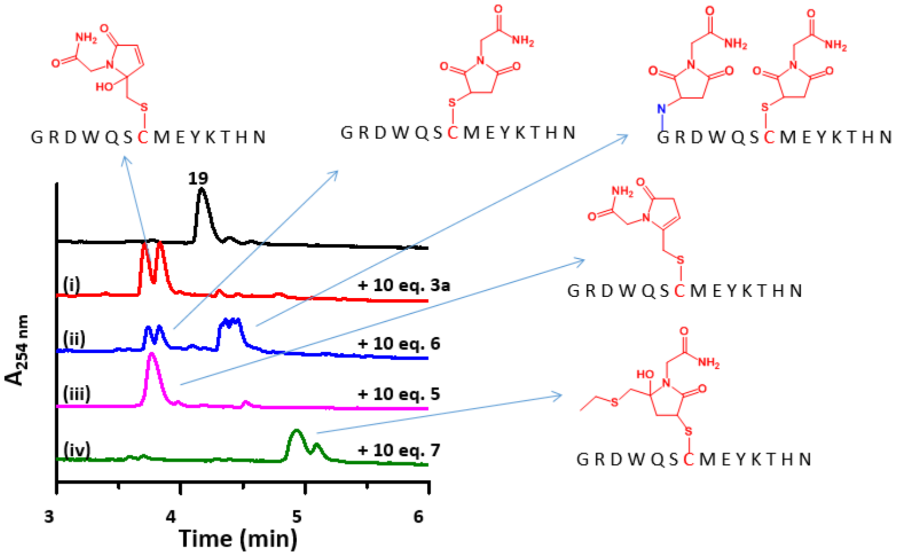

(B) MS/MS analysis of product in (A)-(i) [RT = 3.70, 3.81 min]

Ion	cal (Da)	found (Da)	Ion	cal (Da)	found (Da)
b2	214.1319	214.1304	y1	133.0636	133.0613
b3	329.1589	329.1573	y2	270.1222	270.1202
b4	515.2380	515.2366	y3	371.1693	371.1679
b5	643.2949	643.2952	y4	499.2625	499.2629
b6	730.3267	730.3273	y5	662.3280	662.3262
b7*	1001.3835	1001.3900	y6	791.3659	791.3688
b8*	1132.4343	1132.4304	y7	922.4035	922.4093
b9*	1261.4745	1261.4730	y11*	1594.6447	1594.6418
b11*	1552.6183	1552.6313	y12*	1709.6930	1709.6688
b12*	1653.6705	1653.6790	y14*	1922.7941	1922.7914
b13*	1790.7404	1790.7379			

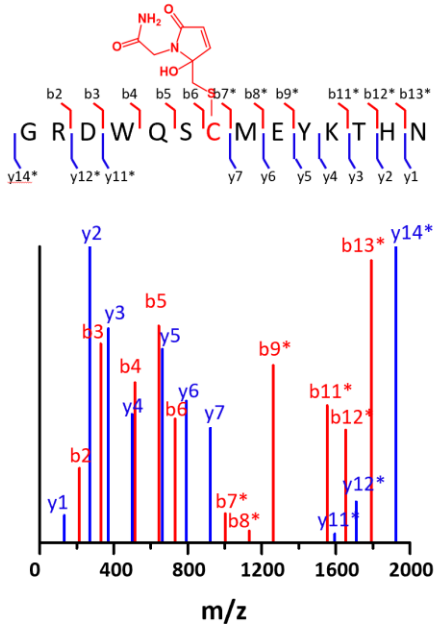

(C) MS/MS analysis of product in (A)-(ii) [RT = 3.77, 3.83 min]

Ion	cal (Da)	found (Da)	Ion	cal (Da)	found (Da)
b2	214.1321	214.1304	y1	133.0630	133.0613
b3	329.1584	329.1573	y2	270.1214	270.1212
b4	515.2370	515.2366	y3	371.1695	371.1679
b5	643.2966	643.2952	y4	499.2634	499.2629
b6	730.3262	730.3273	y5	662.3239	662.3262
b7*	987.3721	987.3743	y6	791.3657	791.3688
b8*	1118.4138	1118.4147	y7	922.4080	922.4093
b9*	1247.4547	1247.4573	y8*	1179.4509	1179.4563
b10*	1410.5166	1410.5206	y9*	1266.4828	1266.4883
b11*	1538.6013	1538.6156	y10*	1394.5330	1394.5469
b12*	1639.6633	1639.6633	y12*	1695.6576	1695.6531
b13*	1776.7180	1776.7222	y14*	1908.7754	1908.7756

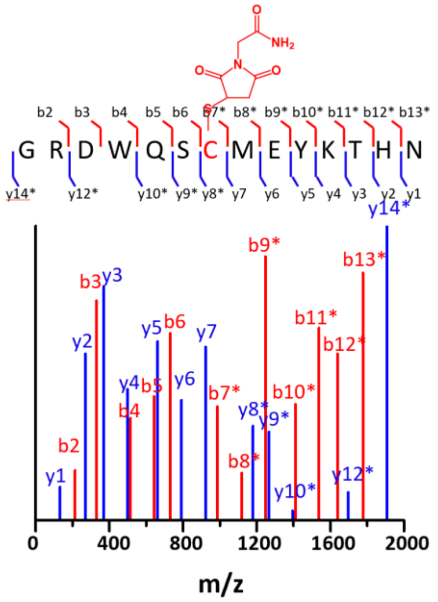

(D) MS/MS analysis of product in (A)-(ii) [RT = 4.41 min]

Ion	cal (Da)	found (Da)	Ion	cal (Da)	found (Da)
b2*	368.1686	368.1682	y1	133.0631	133.0613
b3*	483.1962	483.1951	y2	270.1216	270.1202
b4*	669.2754	669.2744	y3	371.1687	371.1679
b5*	797.3322	797.3330	y4	499.2631	499.2629
b6*	884.3637	884.3651	y5	662.3264	662.3262
b7*	1141.4092	1141.4120	y6	791.3670	791.3688
b8*	1272.4492	1272.4575	y7	922.4077	922.4093
b9*	1401.4882	1401.4951	y8*	1179.4534	1179.4563
b10*	1564.5457	1564.5585	y9*	1266.4852	1266.4883
b11*	1692.6274	1692.6534	y10*	1394.5389	1394.5469
b12*	1793.6989	1793.7010	Y11*	1580.6143	1580.6262
b13*	1930.7559	1930.7600	y12*	1695.6577	1695.6531
			y14*	2062.8142	2062.8135

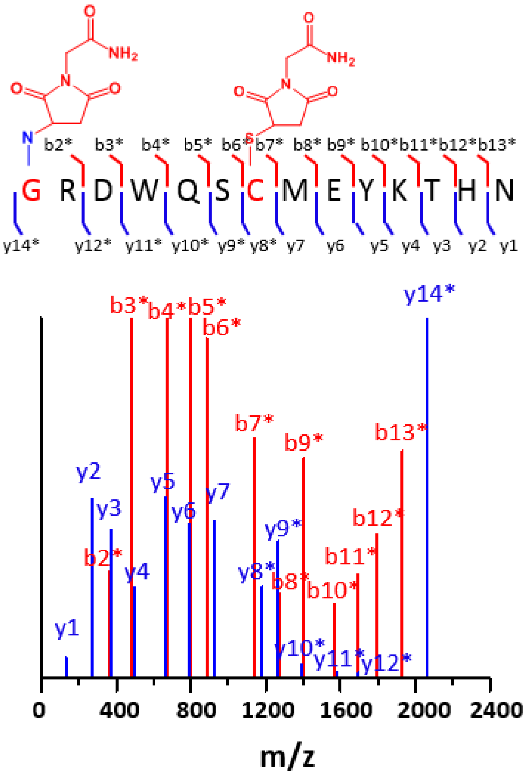

(E) MS/MS analysis of product in (A)-(iii) [RT = 3.98 min]

Ion	cal (Da)	found (Da)	Ion	cal (Da)	found (Da)
b10*	1408.5334	1408.5415	y1	133.0629	133.0613
b11*	1536.6230	1536.6364	y2	270.1214	270.1202
b12*	1637.6814	1637.6841	y3	371.1688	371.1679
b13*	1774.7400	1774.7430	y4	499.2631	499.2629
			y5	662.3245	662.3262
			y6	791.3669	791.3688
			y7	922.4067	922.4093
			y8*	1177.4738	1177.4771
			y9*	1264.5058	1264.5090
			y14*	1906.7958	1906.7965

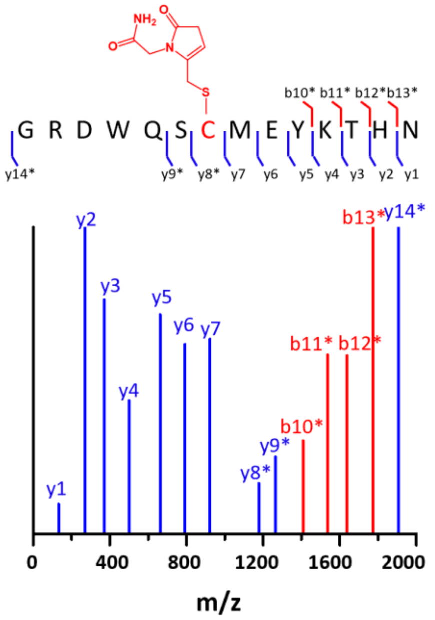

(F) MS/MS analysis of product in (A)-(iv) [RT = 4.93, 5.10 min]

Ion	cal (Da)	found (Da)	Ion	cal (Da)	found (Da)
b2	214.1325	214.1304	y1	133.0626	133.0613
b3	329.1594	329.1573	y2	270.1220	270.1202
b4	515.2377	515.5366	y3	371.1689	371.1679
b5	643.2962	643.2952	y4	499.2630	499.2629
b6	730.3297	730.3273	y5	662.3265	662.3262
b7*	1063.4227	1063.4039	y6	791.3691	791.3688
b8*	1194.4543	1194.4495	y7	922.4096	922.4093
b9*	1323.4946	1323.4921	y8*	1255.5127	1255.4910
b11*	1614.6364	1614.6503	y13*	1927.7834	1927.7889
b12*	1715.6818	1715.6980	y14*	1984.8152	1984.8104
b13*	1852.7592	1852.7570			

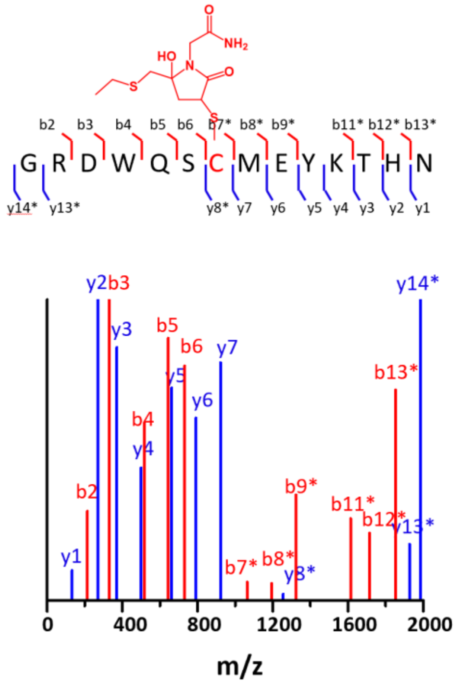

Supplementary Figure 28. UPLC-MS/MS analyses of the reactions of peptide **19** with 10 equiv of **3a**, **5**, **6** or **7**. (A) UPLC profiles. The MS/MS data of products are shown in (B)–(F).

Reaction condition: Peptide **19** (0.2 mM) and substrate **3a**, **5**, **6** or **7** (2 mM, 10 equiv) in HEPES (20 mM, pH 7.5, 100 mM NaCl) incubation at 37 ºC for 1 h. 2 µL of reaction mixture were analyzed by UPLC-MS. UPLC condition (column I): 0-6 min, B: 5-20%, C: 10% keep constant.

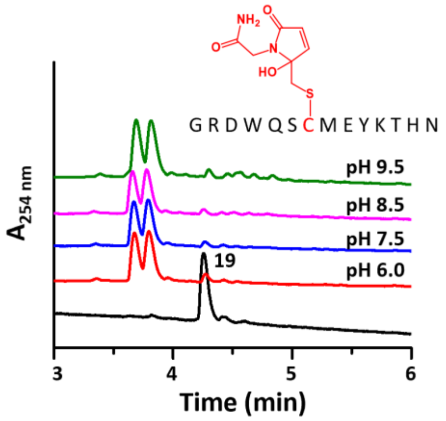

Supplementary Figure 29. UPLC analyses of the reactions of peptide **19** with **3a** in different pH conditions.

Reaction condition: Peptide **19** (0.2 mM) and **3a** (0.4 mM, 2 equiv) in an appropriate buffer (MES, HEPES, HEPPSO, and sodium borate buffer were used for pH 6.0, 7.5, 8.5, 9.5 respectively, final concentration 20 mM, 100 mM NaCl) was incubated at 37 ºC for 1 h. 2 μL of reaction mixture were analyzed by UPLC-MS. UPLC condition (column I): 0-6 min, B: 5-20%, C: 10% keep constant.

(A) Reaction of protein H3-V35C with **3a-f**.

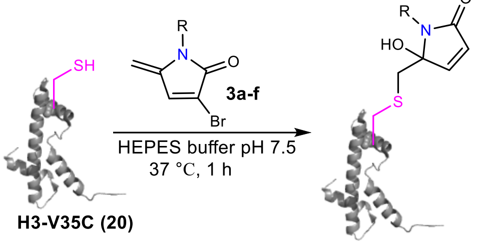

(B) ESI MS of pure protein H3-V35C

H3-V35C [MH]+ cal. 15242

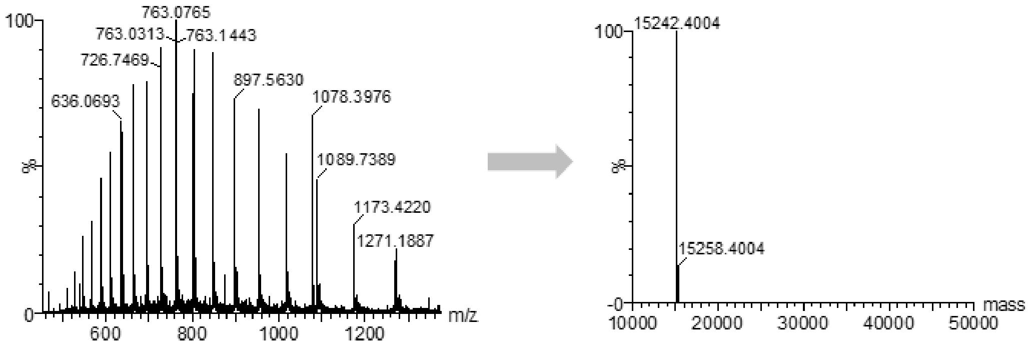

(C) ESI MS of **H3-C35C-3a** conjugate

H3-V35C + 3a [MH]+ cal. 15411

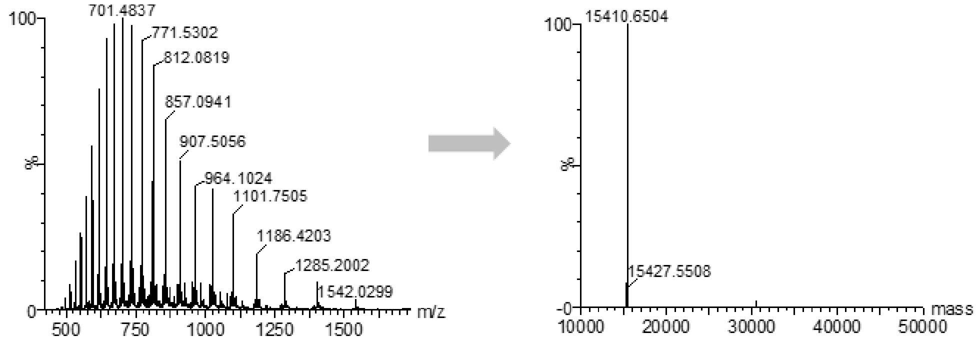

(D) ESI MS of **H3-C35C-3b** conjugate

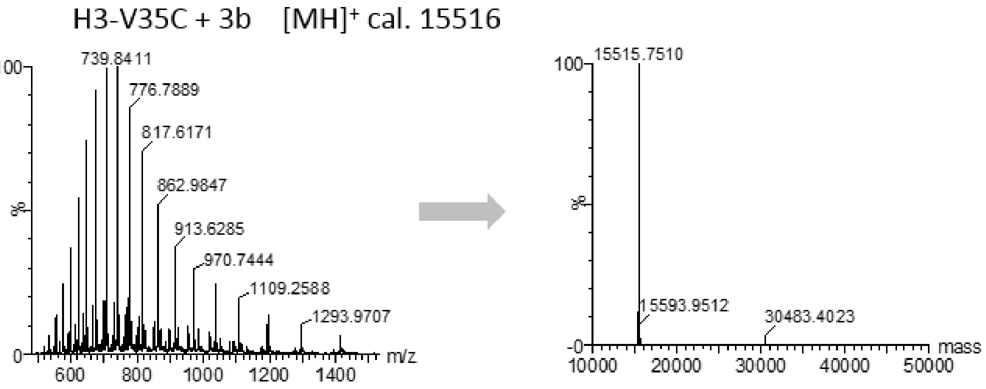

(E) ESI MS of **H3-C35C-3c** conjugate

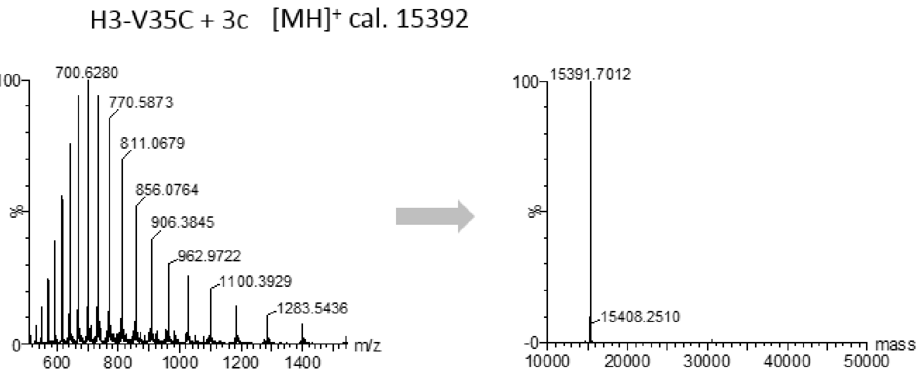

(F) ESI MS of **H3-C35C-3d** conjugate

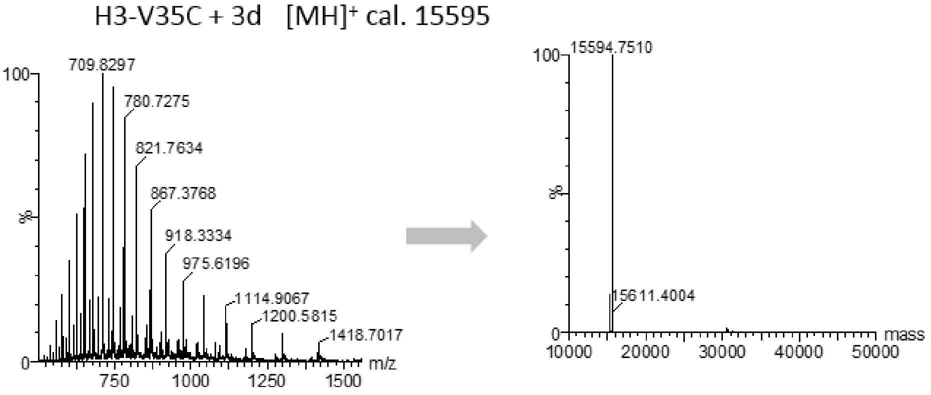

(G) ESI MS of **H3-C35C-3e** conjugate

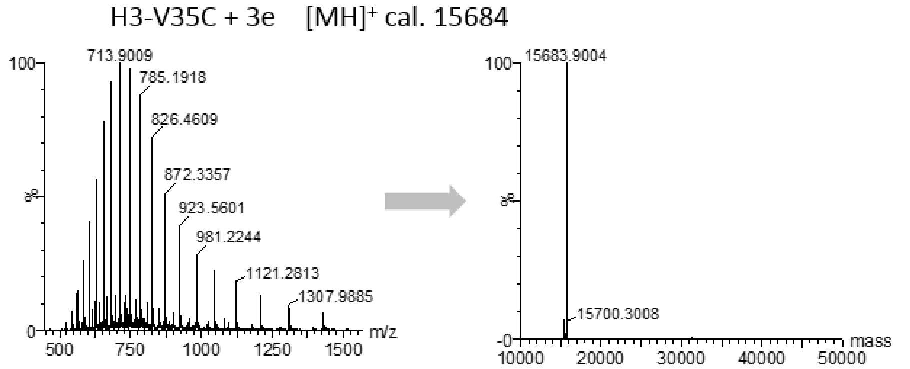

(H) ESI MS of **H3-C35C-3f** conjugate

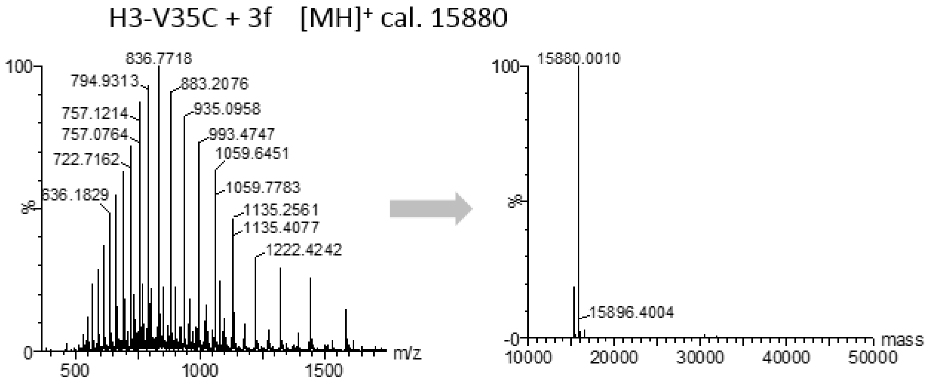

Supplementary Figure 30. MS analyses of the reactions between protein H3-V35C and **3a-f**. (A) Reaction of protein H3-V35C with **3a-f**. The ESI ion series/deconvolution MS spectra of modified products are shown in (B)–(H).

Reaction condition: A mixture of **3a-f** (20 μM, 2 equiv) with H3-V35C (10 μM) in HEPES buffer (10 mM, pH 7.5, 100 mM NaCl) was incubated at 37 ºC for 1 h. 2 μL of the mixtures were taken and analyzed by UPLC-MS. UPLC conditions (column III): 0-7.5 min, B: 2-80%, C: 10% keep constant. The mass of intact protein was obtained by deconvolution of the raw data using MaxEnt1 tool.

(A) Multifunctionalization reactions of protein H3-V35C

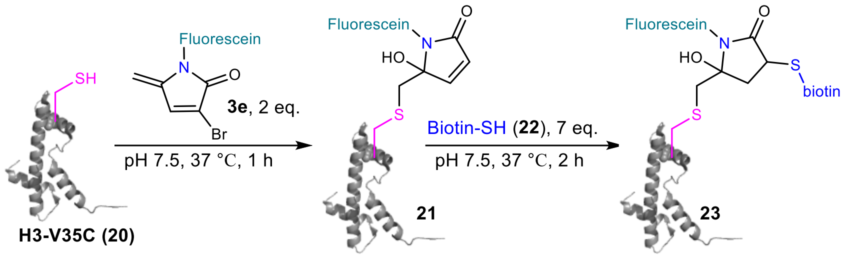

(B) UPLC analyses of protein modification reactions

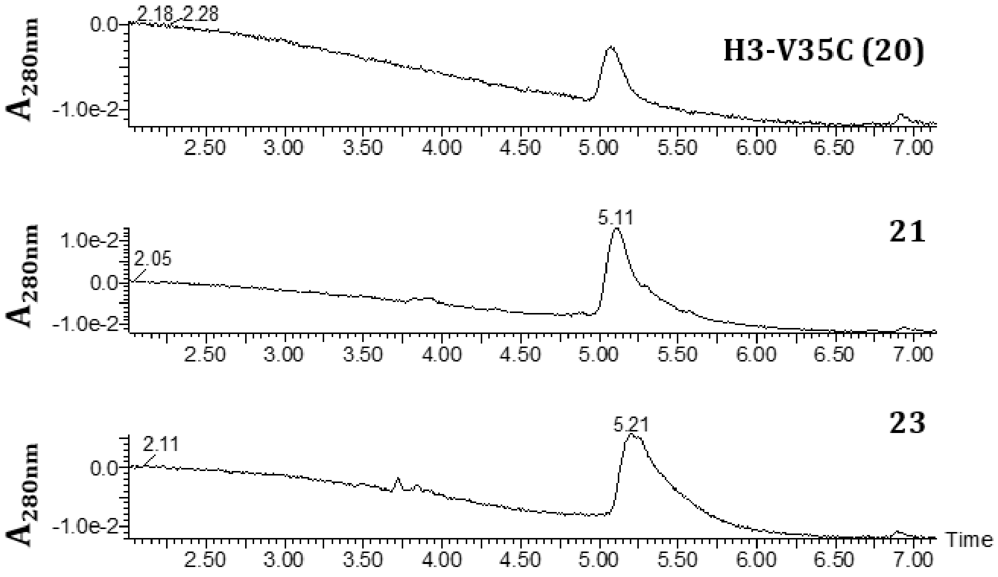

(C) ESI MS of conjugate **21**

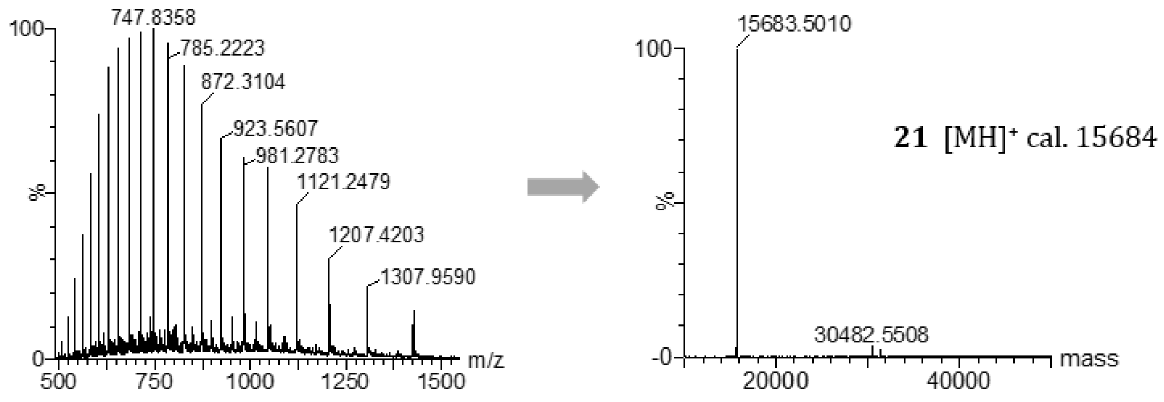

(D) ESI MS of conjugate **23**

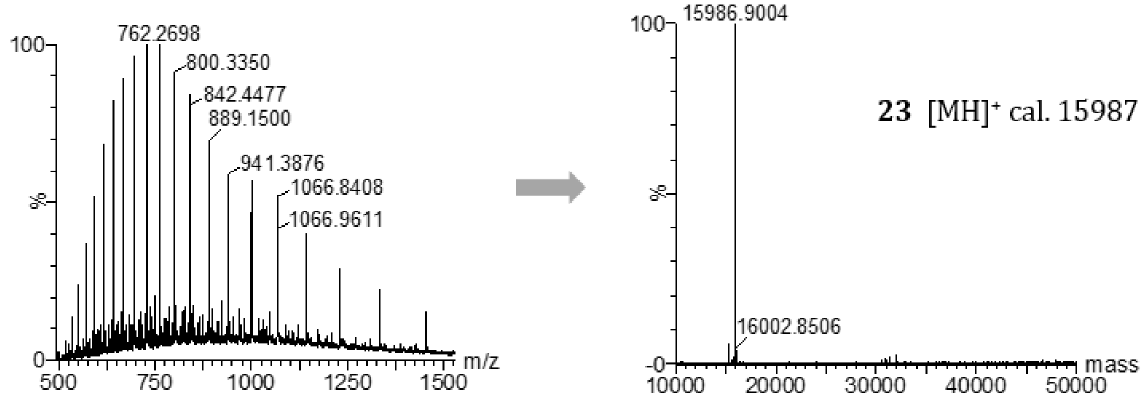

Supplementary Figure 31. UPLC-MS analyses of multifunctionalization of protein H3-V35C. (A) Multifunctionalization reactions of protein H3-V35C. (B) UPLC analyses of protein modification reactions. (C) The ESI ion series/deconvolution MS spectra of modified products **21**. (D) The ESI ion series/deconvolution MS spectra of modified products **23**.

Reaction condition: A mixture of **3e** (200 μM, 2 equiv) and H3-V35C (100 μM) in HEPES buffer (20 mM, pH 7.5, 100 mM NaCl) was incubated at 37 ºC for 1 h to gave **2**. Without purification, Biotin-SH (**22**, 700 μM, 7 equiv) was added to the mixture followed by incubation at 37 ºC for 2 h to gave **23**. The reactions were monitored by UPLC-MS. UPLC conditions (column III): 0-7.5 min, B: 2-80%, C: 10% keep constant. The mass of intact protein was obtained by deconvolution of the raw data using MaxEnt1 tool.

(A) SDS PAGE analysis of the stability of conjugate **23** in pH 7.5 buffer

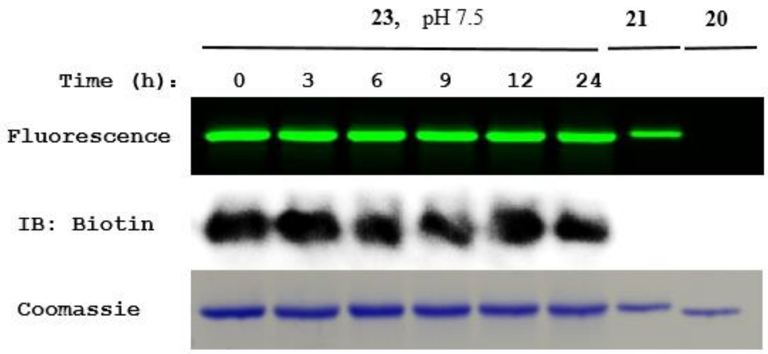

(B) MS analysis the stability of conjugate **23** after incubation in pH 7.5 buffer for 24 h

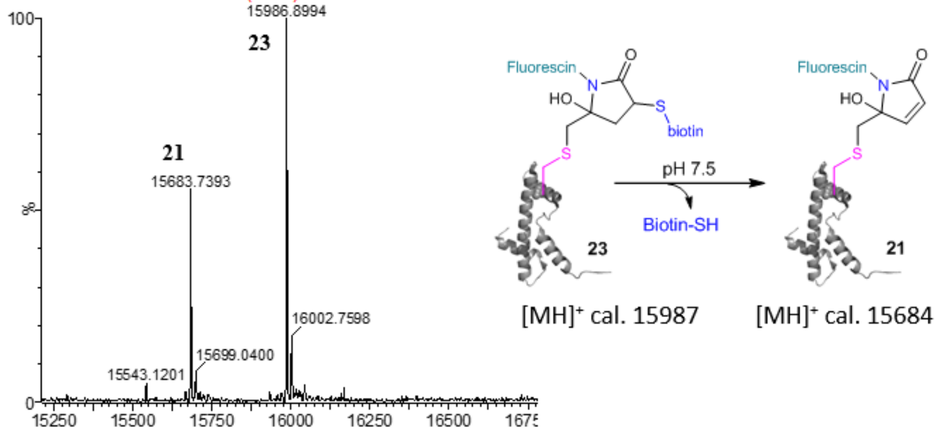

(C) MS analysis the stability of conjugate **23** after incubation in pH 7.5 buffer in the presence of GSH for 24 h

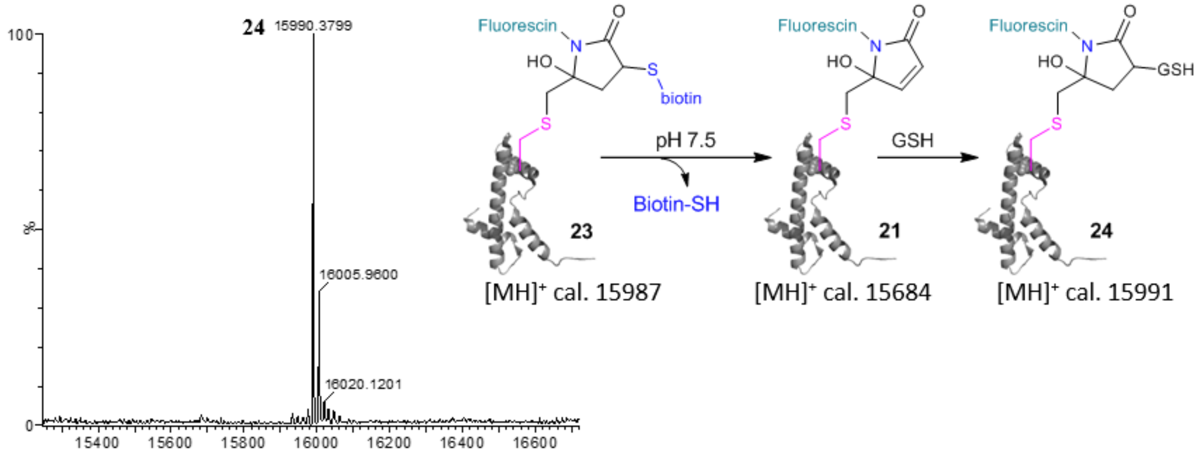

Supplementary Figure 32. Stability of modified protein **23** in neutral buffers. (A) SDS PAGE analysis of the stability of conjugate **23** in pH 7.5 buffer. Top gels: fluorescence. Middle gels: Immunoblotting with antibody against biotin. Bottom gels: Coomassie staining. Source data are provided as a Source Data file. (B) MS analysis the stability of conjugate **23** after incubation in pH 7.5 buffer for 24 h. (C) MS analysis the stability of conjugate **23** after incubation in pH 7.5 buffer in the presence of GSH (1 mM) for 24 h.

Reaction conditions: Protein **23** (40 μM) was incubated in HEPES buffer (20 mM, pH 7.5, 100 mM NaCl, 0.1 μg/μL BSA) at r.t. Aliquots were taken at appropriate intervals and analyzed by 15% SDS-PAGE.

(A) ESI MS of pure protein H3-V35C

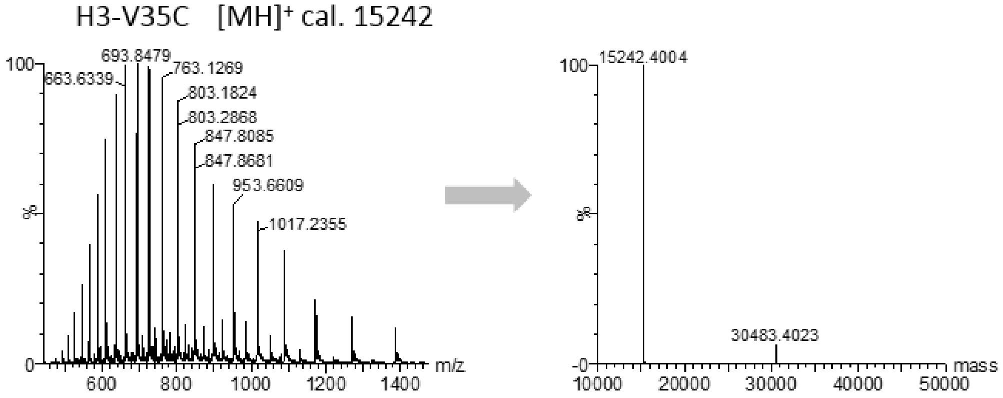

(B) ESI MS of protein H3-V35C upon treatment with 3 mM $NaBH_4$

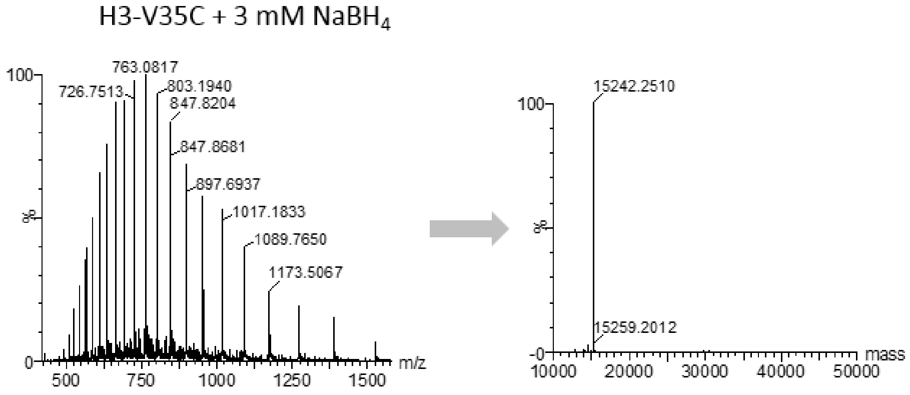

(C) ESI MS of protein H3-V35C upon treatment with 10 mM $NaBH_4$

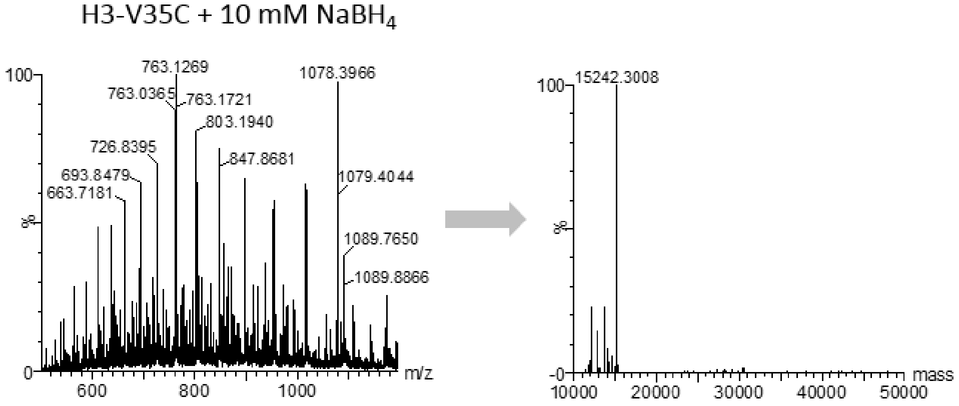

(D) ESI MS of protein H3-V35C upon treatment with 20 mM $NaBH_4$

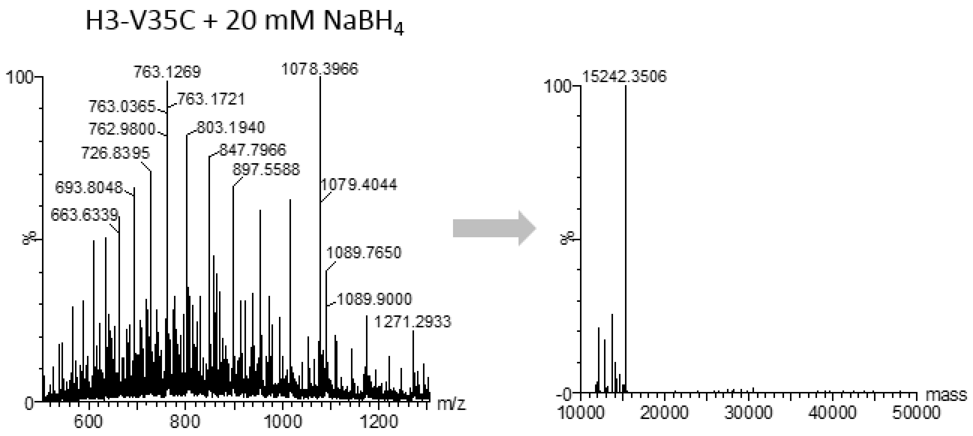

(E) Circular dichroism spectra of protein H3-V35C before and after treatment with 3 mM $NaBH_4$

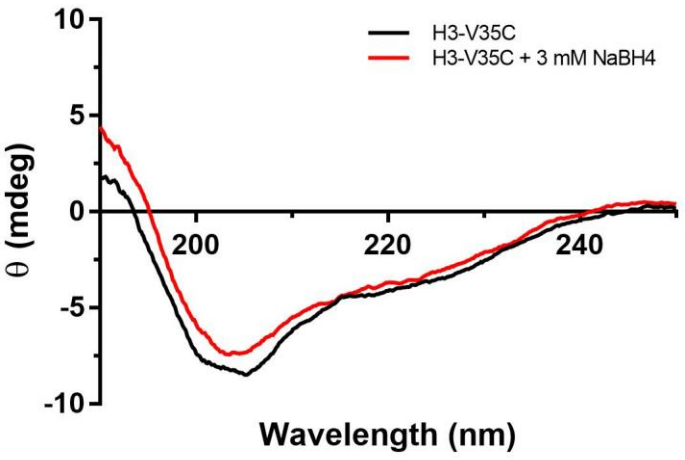

Supplementary Figure 33. Stability of protein H3-V35C upon treatment with $NaBH_4$. (A) The ESI ion series/deconvolution MS spectra of pure protein H3-V35C. (B) The ESI ion series/deconvolution MS spectra of protein H3-V35C upon treatment with 3 mM $NaBH_4$. (C) The ESI ion series/deconvolution MS spectra of protein H3-V35C upon treatment with 10 mM $NaBH_4$. (D) The ESI ion series/deconvolution MS spectra of protein H3-V35C upon treatment with 20 mM $NaBH_4$. (E) Circular dichroism spectra of protein H3-V35C before and after treatment with 3mM $NaBH_4$.

Reaction condition: A mixture of sodium borohydride (0–20 mM) and H3-V35C (10 μM) in HEPES buffer (20 mM, pH 7.5, 100 mM NaCl) was incubated at 37 °C for 1 h.

(A) Reaction of conjugate **23** with $NaBH_4$

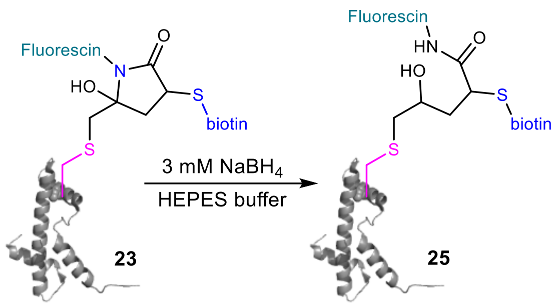

(B) UPLC analyses of the reduction of conjugate **23**

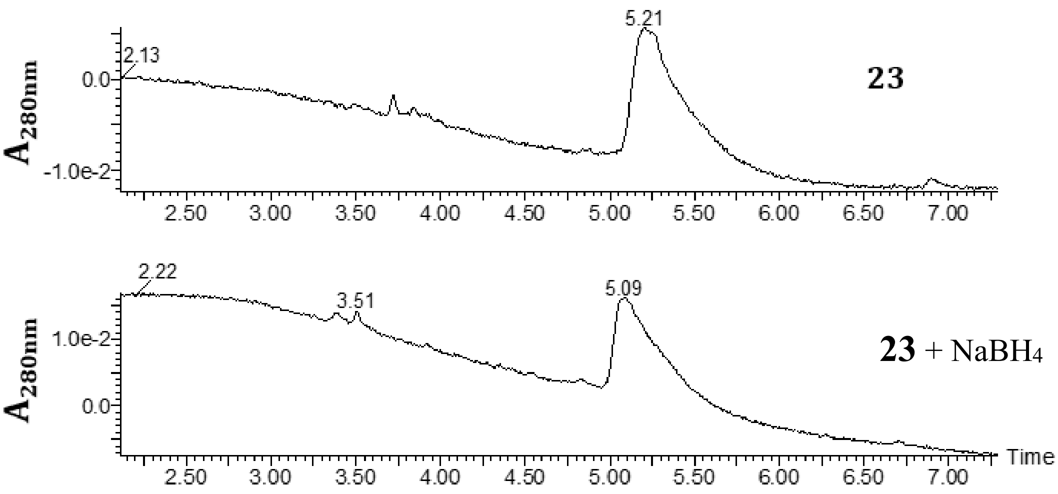

(C) ESI MS of **23**

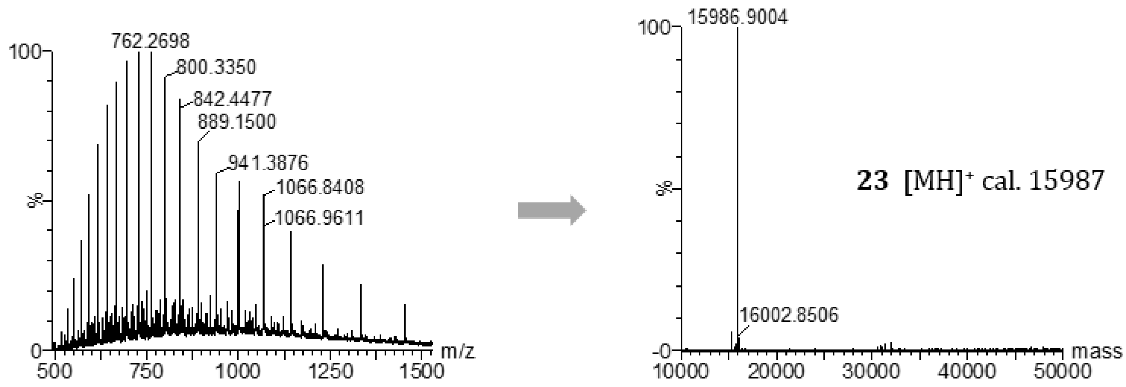

(D) ESI MS of the product **25**

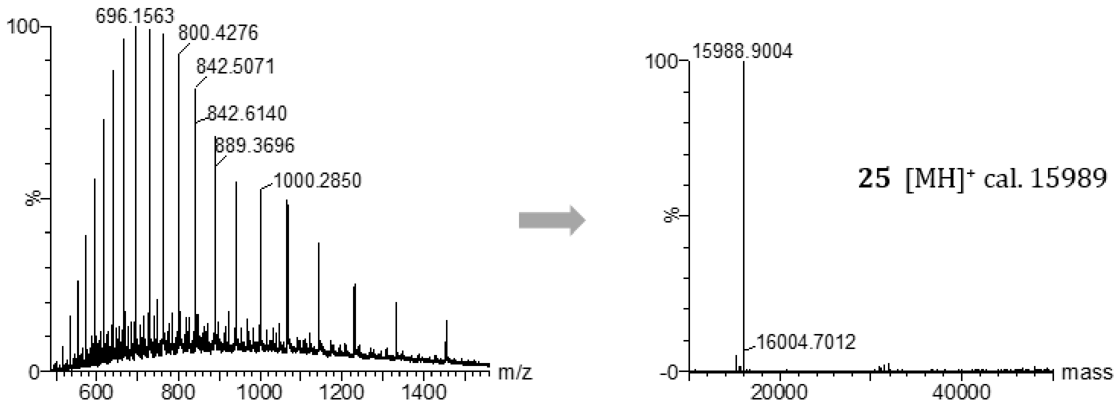

Supplementary Figure 34. UPLC-MS analysis of the reduction of **23** with $NaBH_4$. (A) Reaction of conjugate **23** with $NaBH_4$. (B) UPLC analyses of the reaction of conjugate **23** with $NaBH_4$. (C) The ESI ion series/deconvolution MS spectra of **23**. (D) The ESI ion series/deconvolution MS spectra of the product **25**.

Reaction condition: A mixture of **3e** (200 μM) and H3-V35C (100 μM) in HEPES buffer (20 mM, pH 7.5, 100 mM NaCl) was incubated at 37 ºC for 1 h. Without purification, Biotin-SH (**22**, 700 μM) was added to the solution mixture directly followed by incubation at 37 ºC for 2 h. Then 100 mM freshly prepared $NaBH_4$ (final concentration 3 mM) was added directly and the mixture was incubated at 37 ºC for 40 min. The reaction was monitored by UPLC-MS. UPLC conditions (column III): 0-7.5 min, B: 2-80%, C: 10% keep constant. The mass of intact protein was obtained by deconvolution of the raw data using MaxEnt1 tool.

Ion	cal (Da)	found (Da)	Ion	cal (Da)	found (Da)
b2	159.1767	159.0770	y1	147.1147	147.1133
b3	256.1331	256.1297	y2*	996.3294	996.3306
b4	327.1643	327.1668	y3*	1053.3485	1053.3521
b7	542.2587	542.2574	y4*	1110.730	1110.3734
			y5*	1211.4200	1211.4211
			y6*	1282.4573	1282.4583
			y7*	1379.4181	1379.5110
			y8*	1450.5459	1450.5481
			y9*	1537.5696	1537.5802

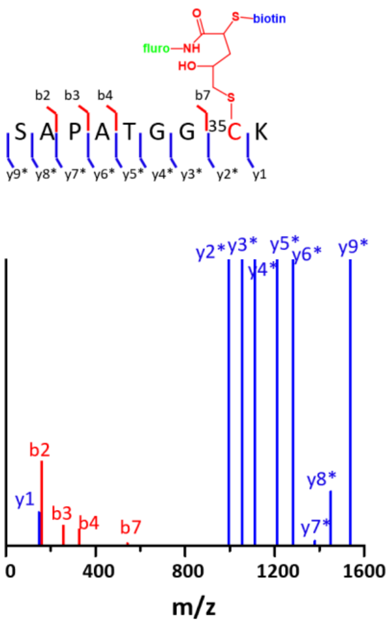

Supplementary Figure 35. MS/MS analysis of tryptic peptide fragment of reduced conjugate **25.**

(A) Reaction of conjugate **21** with $NaBH_4$.

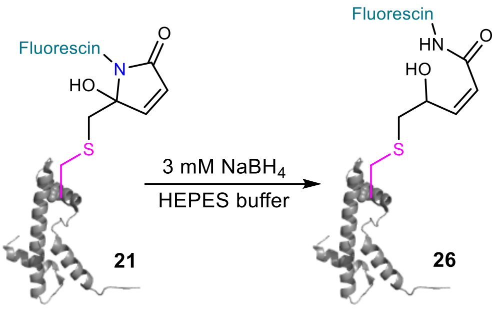

(B) UPLC analyses of the reduction of conjugate **21**.

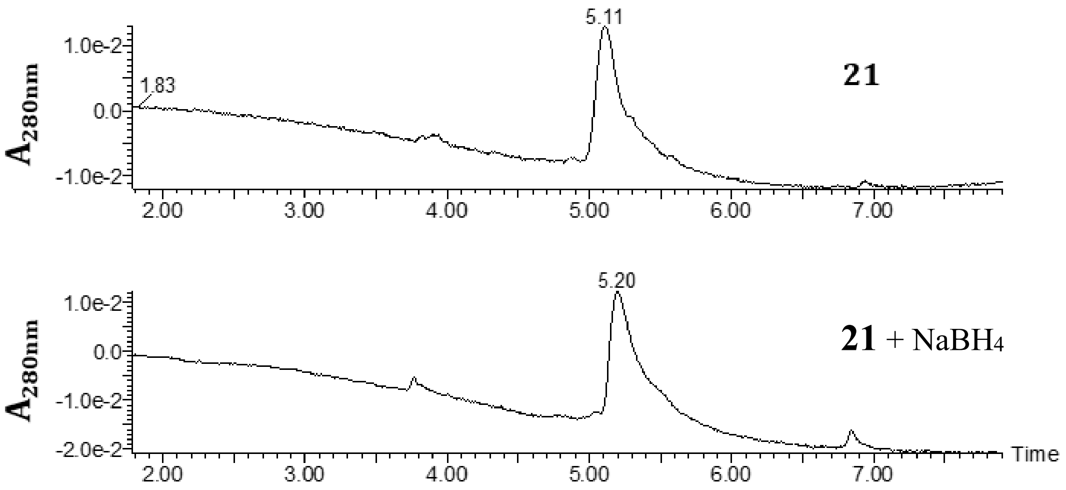

(C) ESI MS of **21**.

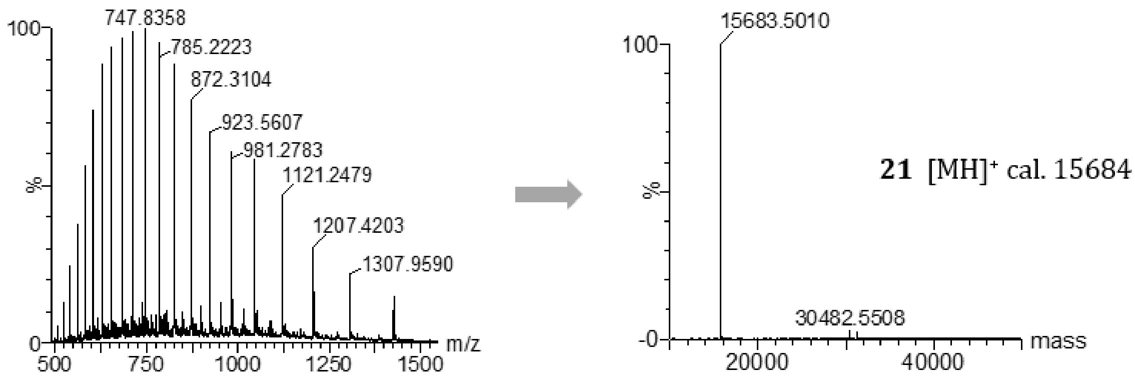

(D) ESI MS of **26**.

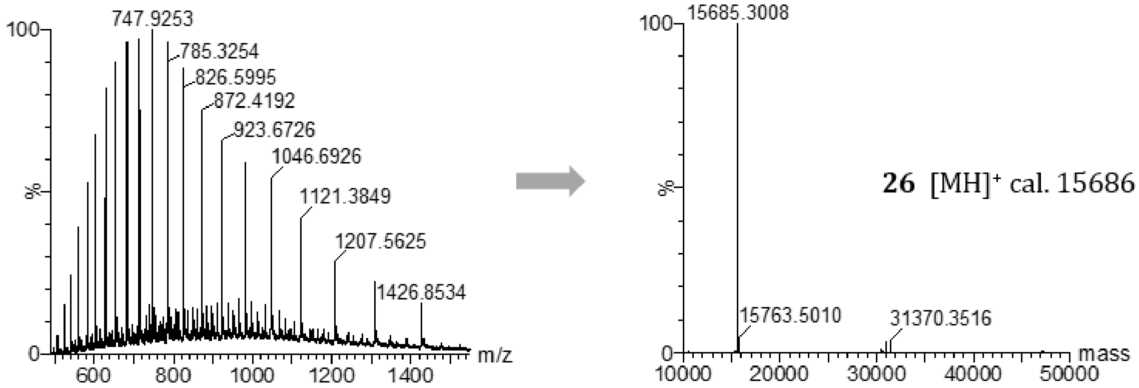

Supplementary Figure 36. UPLC-MS analysis of reduction of **21** with $NaBH_4$. (A) Reaction of conjugate **21** with $NaBH_4$. (B) UPLC analyses of the reaction of conjugate **21** with $NaBH_4$. (C) The ESI ion series/deconvolution MS spectra of **21**. (D) The ESI ion series/deconvolution MS spectra of the product **26**.

Reaction condition: A mixture of **3e** (200 µM) and H3-V35C (100 µM) in HEPES buffer (20 mM, pH 7.5, 100 mM NaCl) was incubated at 37 ºC for 1 h. Then 100 mM freshly prepared $NaBH_4$ (final 3 mM) was added directly into the reaction mixture followed by incubation at 37 ºC for 40 min. The reaction was monitored by UPLC-MS. UPLC conditions (column III): 0-7.5 min, B: 2-80%, C: 10% keep constant. The mass of intact protein was obtained by deconvolution of the raw data using MaxEnt1 tool.

(A) 15% SDS PAGE analyses of the stability of conjugate **26** in pH 7.5 buffer

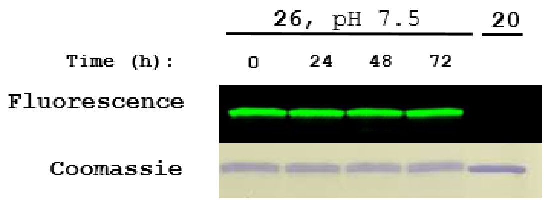

(B) MS of **26** after incubation in pH 7.5 buffer for 72 h

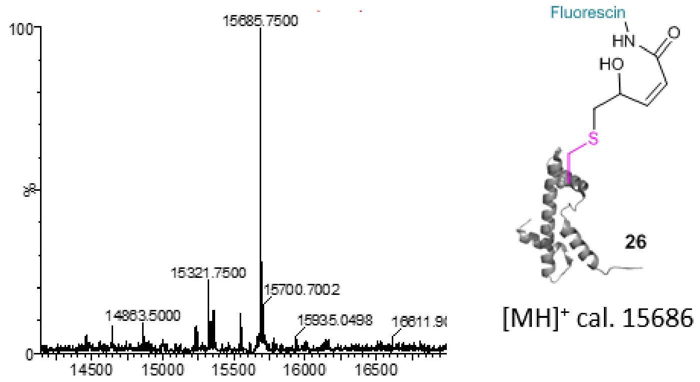

Supplementary Figure 37. Stability of modified protein **26** in neutral buffers. (A) SDS PAGE analysis of the stability of conjugate **26** in pH 7.5 buffer. Source data are provided as a Source Data file. (B) MS analysis the stability of conjugate **26** after incubation in pH 7.5 buffer for 72 h.

Reaction condition: Protein **26** (40 µM) was incubated in HEPES buffer (20 mM, pH 7.5, 100 mM NaCl, 0.1 µg/µL BSA) at r.t. Aliquots were taken at appropriate intervals and analyzed by 15% SDS-PAGE.

(A) UPLC analyses of reactions between SST and **3a**

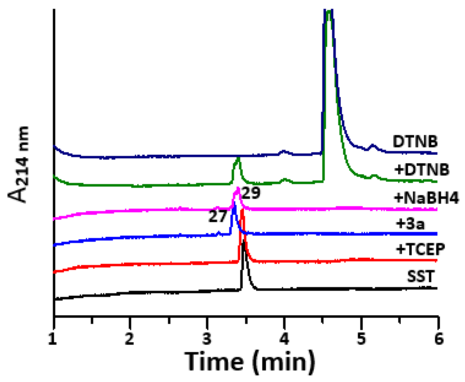

(B) UPLC analyses of reactions between SST and **3e**

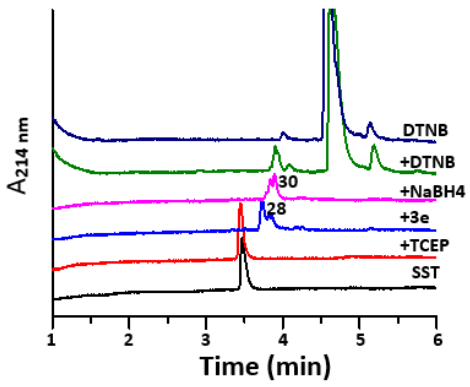

(C) ESI MS of conjugate **27**

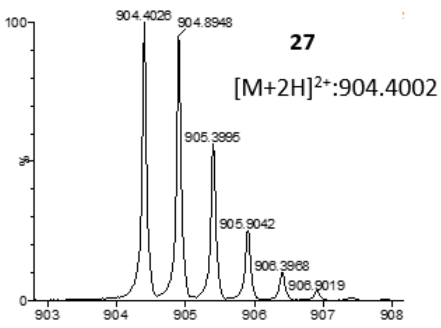

(D) ESI MS of conjugate **28**

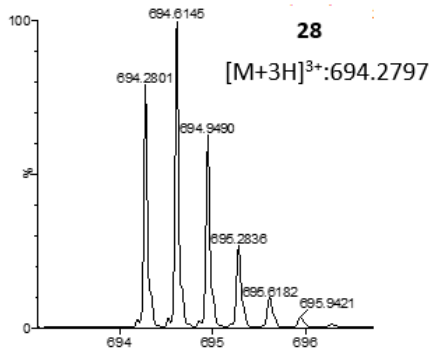

(E) ESI MS of conjugate **29**

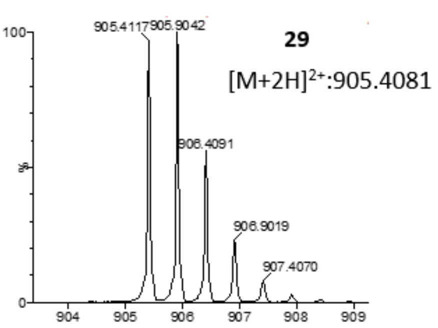

(F) ESI MS of conjugate **30**

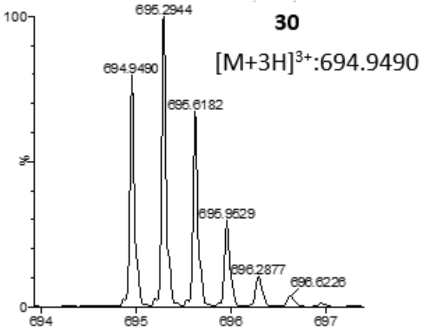

Supplementary Figure 38. UPLC-MS analyses of disulfide bridging bioconjugation of SST using **3a** and **3e**.

Reaction condition: A mixture of TCEP (0.075 mM, 1.5 equiv) and hormone somatostain (SST) (0.05 mM) in HEPES buffer (20 mM, pH 7.5, 100 mM NaCl) was incubated at 37 ºC for 2 h. Without purification, (A) **3a** or (B) **3e** (0.065 mM, 1.3 equiv) were added into the reaction mixture followed by incubation at same temperature for 1 h. Then freshly prepared $NaBH_4$ (final 2.5 mM, 50 equiv) was added followed by incubation for 40 min. The reactions were monitored by UPLC-MS. UPLC condition (column I): 0-6 min, B: 5-60%, C: 10% keep constant.

Products **29** and **30** were purified by HPLC. Then Ellman's reagent was used to test the existence of free cysteine: The solution of **29** and **30** (0.06 mM) in water was treated with Ellman's reagent (1.2 mM in H_2O/DMSO (1/1, v/v), 100 equiv) at 37 ºC for 3 h. After which the mixture was analyzed by UPLC-MS. UPLC condition (column I): 0-6 min, B: 5-60%, C: 10% keep constant. MS spectra are show in (C) for **27**, (D) for **28**, (E) for **29** and (F) for **30**.

Ion	cal (Da)	found (Da)	Ion	cal (Da)	found (Da)
b2	129.0686	129.0664	y2*	377.1231	377.1131
b3*	400.1319	4.1291	y7*	1040.4456	1040.4512
b5*	642.2671	642.2670	y8*	1187.5186	1187.5195
b6*	789.3331	789.3354	y10*	1448.6428	1448.6309
b9*	1250.5720	1250.5780	y13*	1904.8101	1904.7893
b10*	1351.6216	1351.6257	y14*	1975.8420	1975.8472
b11*	1498.9844	1498.6941			
b12*	1599.7382	1599.7418			
b13*	1686.7726	1686.7738			

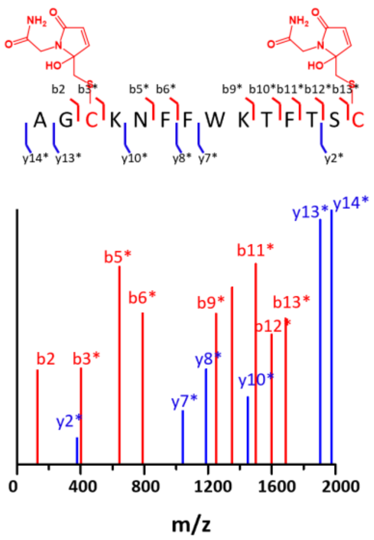

Supplementary Figure 39. MS/MS analysis of modified peptide **31.**

(A)

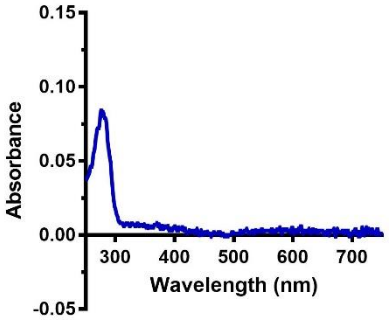

(B)

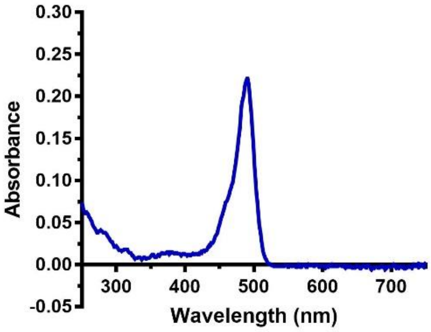

(C)

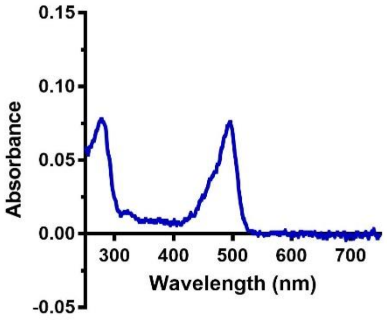

Supplementary Figure 40. UV-Vis spectra of Fab of goat antibody anti-human IgG, fluorescein, and Fab-fluorescein conjugate **33**. (A) UV-Vis spectrum of Fab of goat antibody anti-human IgG. (B) UV-Vis spectrum of fluorescein. (C) UV-Vis spectrum of Fab-fluorescein conjugate **33**.

The degree of labeling (DOL), the average number of fluorescein molecules per antibody, was determined from the quotient of fluorescein concentration to antibody concentration:

$$\text{DOL} = (A_{492}\ \varepsilon_{\text{fluor}}^{-1})/[(A_{280}\text{-CF}\times A_{492})\ \varepsilon_{\text{Fab}}^{-1}] \approx 1.1$$

In the formula, ε_{280} = 76,000 M^{-1} cm^{-1} for goat anti-human Fab, ε_{492} = 80,000 M^{-1} cm^{-1} for fluorescein and 0.18 as a correction factor (CF) for fluorescein for the absorbance at 280 nm.

Supplementary Table 1. Crystal data and structure refinement for shelxl of compound 8.

Identification code	shelxl
Empirical formula	C13 H14 Br N O2 S
Formula weight	328.22
Temperature	113(2) K
Wavelength	0.71073 A
Crystal system, space group	Monoclinic, P2(1) n^-1
Unit cell dimensions	a = 6.7236(13) A alpha = 90 deg.
	b = 8.5880(17) A beta = 96.02(3) deg.
	c = 23.936(5) A gamma = 90 deg.
Volume	1374.5(5) A^3
Z, Calculated density	4, 1.586 Mg m^-3
Absorption coefficient	3.136 mm^-1
F(000)	664
Crystal size	0.200 x 0.180 x 0.120 mm
Theta range for data collection	2.521 to 27.907 deg.
Limiting indices	-8<=h<=8, -11<=k<=11, -31<=l<=30
Reflections collected / unique	16025 / 3263 [R(int) = 0.0633]
Completeness to theta = 25.242	99.6 %
Absorption correction	Semi-empirical from equivalents
Max. and min. transmission	1 and 0.7469
Refinement method	Full-matrix least-squares on F^2
Data / restraints / parameters	3263 / 0 / 166
Goodness-of-fit on F^2	1.000
Final R indices [I>2sigma(I)]	R1 = 0.0452, wR2 = 0.1063
R indices (all data)	R1 = 0.0632, wR2 = 0.1154
Extinction coefficient	0.0253(16)
Largest diff. peak and hole	1.072 and -0.812 e.A^-3

Supplementary References

1 Li, G. & Widom, J. Nucleosomes facilitate their own invasion. *Nat. Struct. Mol. Biol.* **11**, 763-769 (2004).

2 Zhou, C. Z. & Greenberg, M. M. DNA damage by histone radicals in nucleosome core particles. *J. Am. Chem. Soc.* **136**, 6562-6565 (2014).

3 Zhang, Y. *et al.* Thiol specific and tracelessly removable bioconjugation via michael addition to 5-methylene pyrrolones. *J. Am. Chem. Soc.* **139**, 6146-6151 (2017).

4 Ota, E. *et al.* Synthesis and biological evaluation of molecular probes based on the 9-methylstreptimidone derivative dtcm-glutarimide. *Bioorg. Med. Chem. Lett.* **22**, 164-167 (2012).

5 Smith, M. E. B. *et al.* Protein modification, bioconjugation, and disulfide bridging using bromomaleimides. *J. Am. Chem. Soc.* **132**, 1960-1965 (2010).
