# Supplementary material for: Cysteine-specific protein multi-functionalization and disulfide bridging using 3-bromo-5-methylene pyrrolones
Source: Nat Commun. 2020 Feb 21;11:1015. doi: 10.1038/s41467-020-14757-4 (PMC7035330; doi:10.1038/s41467-020-14757-4)
Supplement: Supplementary file 3 — Description of Additional Supplementary Files [file 41467_2020_14757_MOESM3_ESM.pdf]

**Description of Additional Supplementary Files**

File Name: Supplementary Data 1
Description: Crystal structure of compound **8**.
