# Supplementary material for: Cysteine-specific protein multi-functionalization and disulfide bridging using 3-bromo-5-methylene pyrrolones
Source: Nat Commun. 2020 Feb 21;11:1015. doi: 10.1038/s41467-020-14757-4 (PMC7035330; doi:10.1038/s41467-020-14757-4)

checkCIF/PLATON report

Structure factors have been supplied for datablock(s) shelxl

THIS REPORT IS FOR GUIDANCE ONLY. IF USED AS PART OF A REVIEW PROCEDURE FOR PUBLICATION, IT SHOULD NOT REPLACE THE EXPERTISE OF AN EXPERIENCED CRYSTALLOGRAPHIC REFEREE.

No syntax errors found.   CIF dictionary   Interpreting this report

Datablock: shelxl

```
Bond precision:  C-C = 0.0041 A          Wavelength=0.71073

Cell:            a=6.7236(13)     b=8.5880(17)      c=23.936(5)
                 alpha=90         beta=96.02(3)     gamma=90
Temperature:     113 K

                Calculated                Reported
Volume          1374.5(5)                 1374.5(5)
Space group     P 21/n                    P 21/n
Hall group      -P 2yn                    -P 2yn
Moiety formula  C13 H14 Br N O2 S         ?
Sum formula     C13 H14 Br N O2 S         C13 H14 Br N O2 S
Mr              328.21                    328.22
Dx,g cm-3       1.586                     1.586
Z               4                         4
Mu (mm-1)       3.136                     3.136
F000            664.0                     664.0
F000'           663.60
h,k,lmax        8,11,31                   8,11,31
Nref            3286                      3263
Tmin,Tmax       0.539,0.686               0.747,1.000
Tmin'           0.529

Correction method= # Reported T Limits: Tmin=0.747 Tmax=1.000
AbsCorr = MULTI-SCAN

Data completeness= 0.993          Theta(max)= 27.907

R(reflections)= 0.0452( 2410)     wR2(reflections)= 0.1154( 3263)

S = 1.000                  Npar= 166
```

The following ALERTS were generated. Each ALERT has the format
test-name_ALERT_alert-type_alert-level.
Click on the hyperlinks for more details of the test.

Alert level C

```
PLAT480_ALERT_4_C Long H...A H-Bond Reported H11A     ..BR1      .       3.07 Ang.
PLAT906_ALERT_3_C Large K Value in the Analysis of Variance ......      3.187 Check
PLAT911_ALERT_3_C Missing FCF Refl Between Thmin & STh/L=    0.600          9 Report
PLAT918_ALERT_3_C Reflection(s) with I(obs) much Smaller I(calc) .          1 Check
PLAT934_ALERT_3_C Number of (Iobs-Icalc)/SigmaW > 10 Outliers ....          1 Check
PLAT977_ALERT_2_C Check Negative Difference Density on H12A             -0.46 eA-3
PLAT977_ALERT_2_C Check Negative Difference Density on H13A             -0.60 eA-3
PLAT978_ALERT_2_C Number C-C Bonds with Positive Residual Density.          0 Info
```

Alert level G

```
PLAT007_ALERT_5_G Number of Unrefined Donor-H Atoms .............           1 Report
PLAT793_ALERT_4_G Model has Chirality at C10         (Centro SPGR)          S Verify
PLAT883_ALERT_1_G No Info/Value for _atom_sites_solution_primary .     Please Do !
PLAT910_ALERT_3_G Missing # of FCF Reflection(s) Below Theta(Min).          1 Note
PLAT912_ALERT_4_G Missing # of FCF Reflections Above STh/L=  0.600         14 Note
```

```
   0 ALERT level A = Most likely a serious problem - resolve or explain
   0 ALERT level B = A potentially serious problem, consider carefully
   8 ALERT level C = Check. Ensure it is not caused by an omission or oversight
   5 ALERT level G = General information/check it is not something unexpected

   1 ALERT type 1 CIF construction/syntax error, inconsistent or missing data
   3 ALERT type 2 Indicator that the structure model may be wrong or deficient
   5 ALERT type 3 Indicator that the structure quality may be low
   3 ALERT type 4 Improvement, methodology, query or suggestion
   1 ALERT type 5 Informative message, check
```

It is advisable to attempt to resolve as many as possible of the alerts in all categories. Often the minor alerts point to easily fixed oversights, errors and omissions in your CIF or refinement strategy, so attention to these fine details can be worthwhile. In order to resolve some of the more serious problems it may be necessary to carry out additional measurements or structure refinements. However, the purpose of your study may justify the reported deviations and the more serious of these should normally be commented upon in the discussion or experimental section of a paper or in the "special_details" fields of the CIF. checkCIF was carefully designed to identify outliers and unusual parameters, but every test has its limitations and alerts that are not important in a particular case may appear. Conversely, the absence of alerts does not guarantee there are no aspects of the results needing attention. It is up to the individual to critically assess their own results and, if necessary, seek expert advice.

Publication of your CIF in IUCr journals

A basic structural check has been run on your CIF. These basic checks will be run on all CIFs submitted for publication in IUCr journals (*Acta Crystallographica*, *Journal of Applied Crystallography*, *Journal of Synchrotron Radiation*); however, if you intend to submit to *Acta Crystallographica Section C* or *E* or *IUCrData*, you should make sure that full publication checks are run on the final version of your CIF prior to submission.

Publication of your CIF in other journals

Please refer to the *Notes for Authors* of the relevant journal for any special instructions relating to CIF submission.

PLATON version of 03/05/2019; check.def file version of 29/04/2019

Datablock shelxl - ellipsoid plot

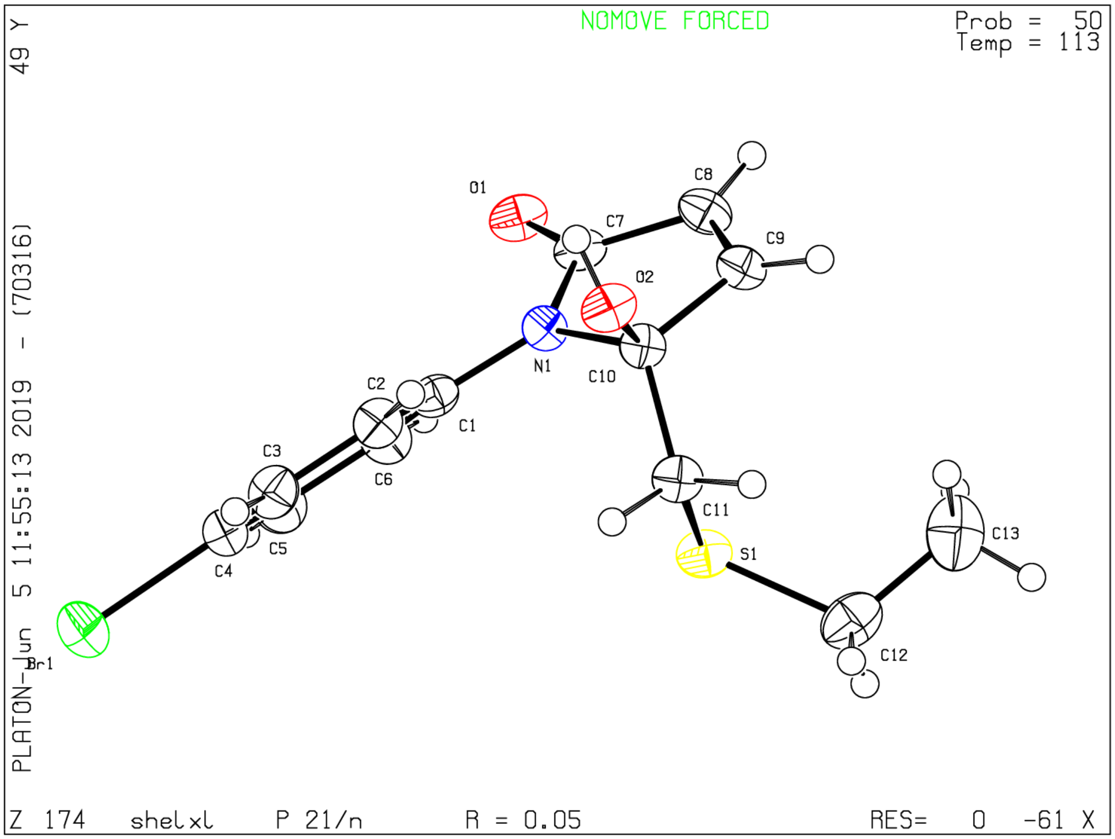

Supplement: Supplementary file 4 — Supplementary Data 1 [file 41467_2020_14757_MOESM4_ESM.zip › checkcif.pdf]
