# Supplementary figures and images for: Cysteine-specific protein multi-functionalization and disulfide bridging using 3-bromo-5-methylene pyrrolones
Source: Nat Commun. 2020 Feb 21;11:1015. doi: 10.1038/s41467-020-14757-4 (PMC7035330; doi:10.1038/s41467-020-14757-4)

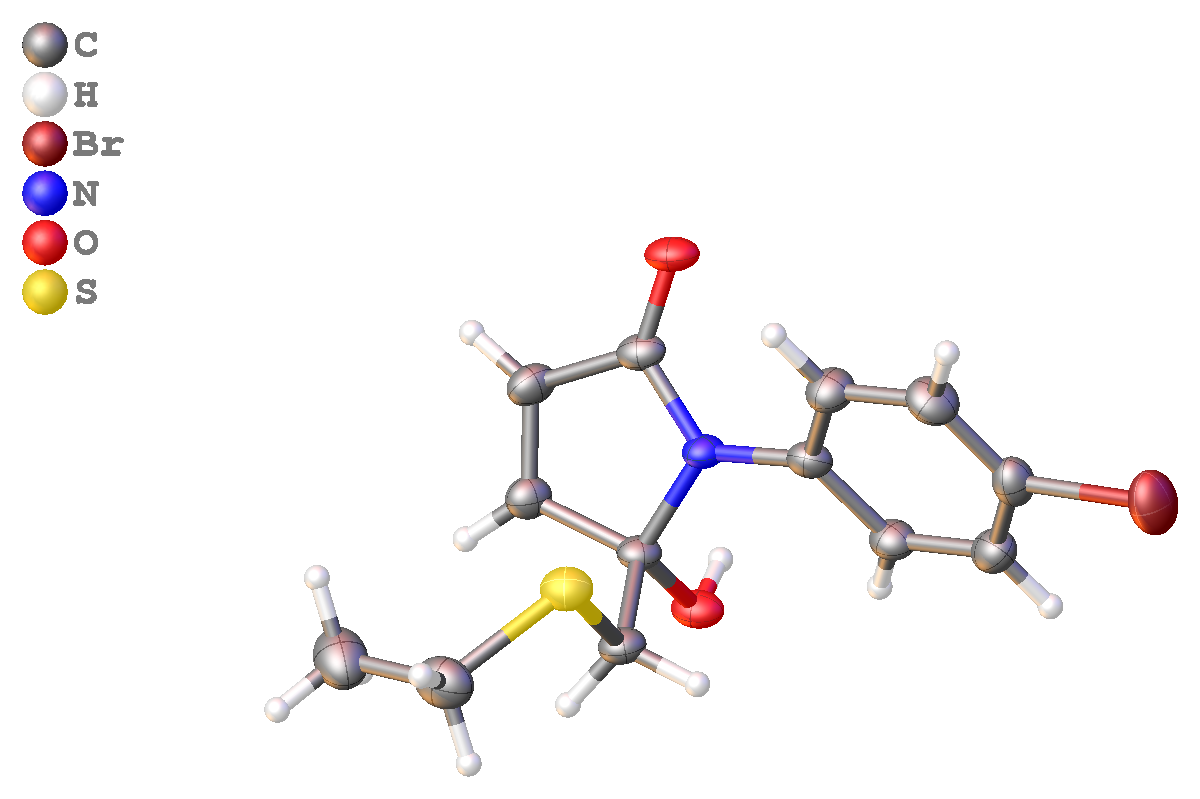

Supplement: Supplementary file 4 — Supplementary Data 1 [file 41467_2020_14757_MOESM4_ESM.zip › shelxl.png]
